# Conservation priorities for terrestrial mammals in Dobrogea Region, Romania

**DOI:** 10.3897/zookeys.792.25314

**Published:** 2018-10-23

**Authors:** Iulia V. Miu, Gabriel B. Chisamera, Viorel D. Popescu, Ruben Iosif, Andreea Nita, Steluta Manolache, Viorel D. Gavril, Ioana Cobzaru, Laurentiu Rozylowicz

**Affiliations:** 1 University of Bucharest, Center for Environmental Research and Impact Studies, 1 N. Balcescu, 010041, Bucharest, Romania; 2 National Museum of Natural History Grigore Antipa, 1 Kiseleff Blvd., 011341, Bucharest, Romania; 3 Department of Biological Sciences, Ohio University, Athens, OH, USA; 4 University Ovidius Constanţa, Faculty of Natural Sciences and Agricultural Sciences, 1 Al. Universităţii, corp B, 900470, Constanţa, Romania; 5 University of Bucharest, Faculty of Biology, 91–95 Splaiul Independenței, 050095, Bucharest, Romania; 6 Romanian Academy, Institute of Biology, 296 Splaiul Independentei, 060031 Bucharest, Romania

**Keywords:** Dobrogea, Natura 2000, species distribution, species richness, systematic conservation planning, terrestrial mammals

## Abstract

Based on species occurrence records of museum collections, published literature, and unpublished records shared by mammalian experts, we compiled a distribution database for 59 terrestrial mammals populating the extensively protected Dobrogea Region of Romania. The spatial patterns of mammal distribution and diversity was evaluated and systematic conservation planning applied to identify priority areas for their conservation. The spatial analyses revealed that intensive sampling was not directly correlated to mammal diversity but rather to accessibility for inventory. The spatial prioritisation analysis indicated a relatively aggregated pattern of areas with a high or low conservation value with virtually no connecting corridors between them. The significant overlap between Natura 2000 sites and national protected areas induced an over-optimistic vision of the effectiveness and representativeness of existing Natura 2000 network for species found in Annexes II and IV of the Habitats Directive. These results represent a key step in identifying core areas for the protection of mammal diversity and dispersal corridors for improved connectivity, and to guide future conservation efforts in increasing the effectiveness of the existing protected areas in the context of environmental changes.

## Introduction

Terrestrial mammals are well-studied taxa, yet their distribution and conservation status are not fully understood (Crooks et al. 2011). Mammalian population decline accelerates the loss of ecosystem services and poses a substantial threat to species diversity at the community level (Ceballos 2002, Rodrigues et al. 2004). Since mammals display diverse traits and can exploit a wide range of ecological niches, they are also effective focal species for conservation, and their population status might be a proxy for both fragmentation and connectivity across landscapes (Crooks et al. 2011).

A common conservation strategy to prevent the loss of biodiversity is the creation of protected areas (Margules and Pressey 2000, Williams et al. 2002). Protected areas must ensure the long-term persistence and viability of species and should ideally support many rare, threatened, or endemic taxa, particularly those with low mobility and high sensitivity to environmental alterations (Rodrigues et al. 2004, Possingham et al. 2006). However, typically, the effectiveness of protected areas is undermined by poor governance (Eklund et al. 2011, Manolache et al. 2018, Nita et al. 2018) and lack of funding and relevant resources (Sánchez-Fernández et al. 2017).

The Natura 2000 network of protected areas of European importance represents one of the most extensive networks of conservation areas worldwide (Nita et al. 2017). Scientists and policymakers often question the effectiveness of this network due to the Member States allocating fewer funds than needed to implement conservation programs (Nita et al. 2017, Sánchez-Fernández et al. 2017). Natura 2000 is more effective in protecting species listed in Birds Directive because of a better overlap between ancillary conservation investments such as Common Agricultural Policy and biodiversity value (Lung et al. 2014, Maiorano et al. 2015), and because birds are more intensely studied than other vertebrate groups. To be more effective, Natura 2000 network must incorporate potential changes in species distributions (Popescu et al. 2013, Kukkala et al. 2016). Failure to acknowledge changes in species ranges may lead to gaps in protecting species that are sensitive to climate change and other anthropogenic pressures (Araújo et al. 2011).

One of Romania’s legal obligations since joining the European Union in 2007 was to designate Natura 2000 sites in a short time (Ioja et al. 2010). Due to the lack of adequate species and habitat distribution data, regions that already benefited from protection under national laws were preferred for the first phase of the designation process. Consequently, the EU conservation goals were not met, which resulted in the designation of additional protected areas (Ioja et al. 2010, Popescu et al. 2013) and a disproportionate increase of land protected in some regions such as it is the case of Dobrogea (now 63% under protection, 9700 km^2^). The Natura 2000 network from Dobrogea includes 67 sites (35 Sites of Community Importance - SCI and 32 Special Protection Areas – SPA, most of the SCIs and SPAs spatially overlap). Within Dobrogea, highlands and floodplains gained extensive protection while lowlands occupied by arable lands remained largely unprotected. However, the latter areas are inhabited by endangered species such as the marbled polecat (*Vormelaperegusna*) and the steppe polecat (*Mustelaeversmanii*) (Murariu et al. 2009, 2010).

Due to the diverse landforms, climatic influences, and habitats, Dobrogea harbours a large number of mammal species (Murariu 1996, Murariu et al. 2010). To date, 59 mammal species have been documented in this region, three of which reach the outer limit of their geographic range (the marbled polecat *Vormelaperegusna*, the stoat *Mustelaerminea*, and the common hamster *Cricetuscricetus*), and two other species have their core range in Dobrogea (the Romanian hamster *Mesocricetusnewtoni* and the Southern birch mouse *Sicistanordmanni*) (Bunescu 1959, 1961, Popescu and Murariu 2001, Murariu and Munteanu 2005). Of the 59 mammal species, 14 are protected by Habitats Directive. Despite the focus of many Natura 2000 sites within Dobrogea on protecting mammal species, limited and outdated distributional databases are available for individual species, e.g., the Romanian hamster *Mesocricetusnewtoni* (Hamar and Schutowa 1966), the Eurasian beaver *Castorfiber* (Kiss et al. 2012, Kiss et al. 2014), the European mink *Mustelalutreola* (Cuzic and Marinov 2004), and the Southern birch mouse *Sicistanordmanni* (Ausländer and Hellwing 1957). Moreover, with few exceptions, (e.g., Murariu 1996, 2006, Murariu et al. 2009, Murariu et al. 2010) the Dobrogea Region lacks actual regional species distribution data.

One tool supporting management decisions and for investigating species population coverage within protected areas is spatial conservation prioritisation (Pouzols et al. 2014). As part of systematic conservation planning (Margules and Pressey 2000) and accounting for complementarity, spatial prioritisation can be an efficient instrument in identifying spatial priorities and in achieving conservation goals (Pressey et al. 2007) even in broadly protected and underfunded regions such as Dobrogea (Rozylowicz et al. 2017). In this study, we evaluate priority areas for mammal conservation in Dobrogea, Romania and assess the spatial patterns of distribution and diversity of terrestrial mammals by: (1) compiling mammal distribution records from published papers, museum records, and unpublished data, (2) analyzing spatial patterns of distribution data, and (3) using systematic conservation planning in identifying high priority areas for conservation of terrestrial mammal listed in Annexes II and IV of Habitats Directive within the regional Natura 2000 network.

## Materials and methods

### Mammal species occurrences

To map the distribution of mammals in Dobrogea, we extracted species occurrence records from three primary sources: museum collections, published data, and unpublished field data. Occurrences that could not be georeferenced to a location (e.g., assigned to a large watershed or geographical province), or associated with unspecified taxa within genera, were not included in this geodatabase. The species taxonomy considered in this paper is based on Wilson and Reeder (2005) and Arslan et al. (2016). Sibling species which are difficult to discriminate in the field, such as the yellow-necked mouse *Apodemusflavicollis*, the wood mouse *Apodemussylvaticus* (Bartolommei et al. 2016), the common vole *Microtusarvalis*, and the East European vole *Microtuslevis* (Jaarola et al. 2004), were included as individual species, as their occurrences were acquired through museum collections and published data. Red list status was based on Temple and Terry (2009).

**Figure 1.**
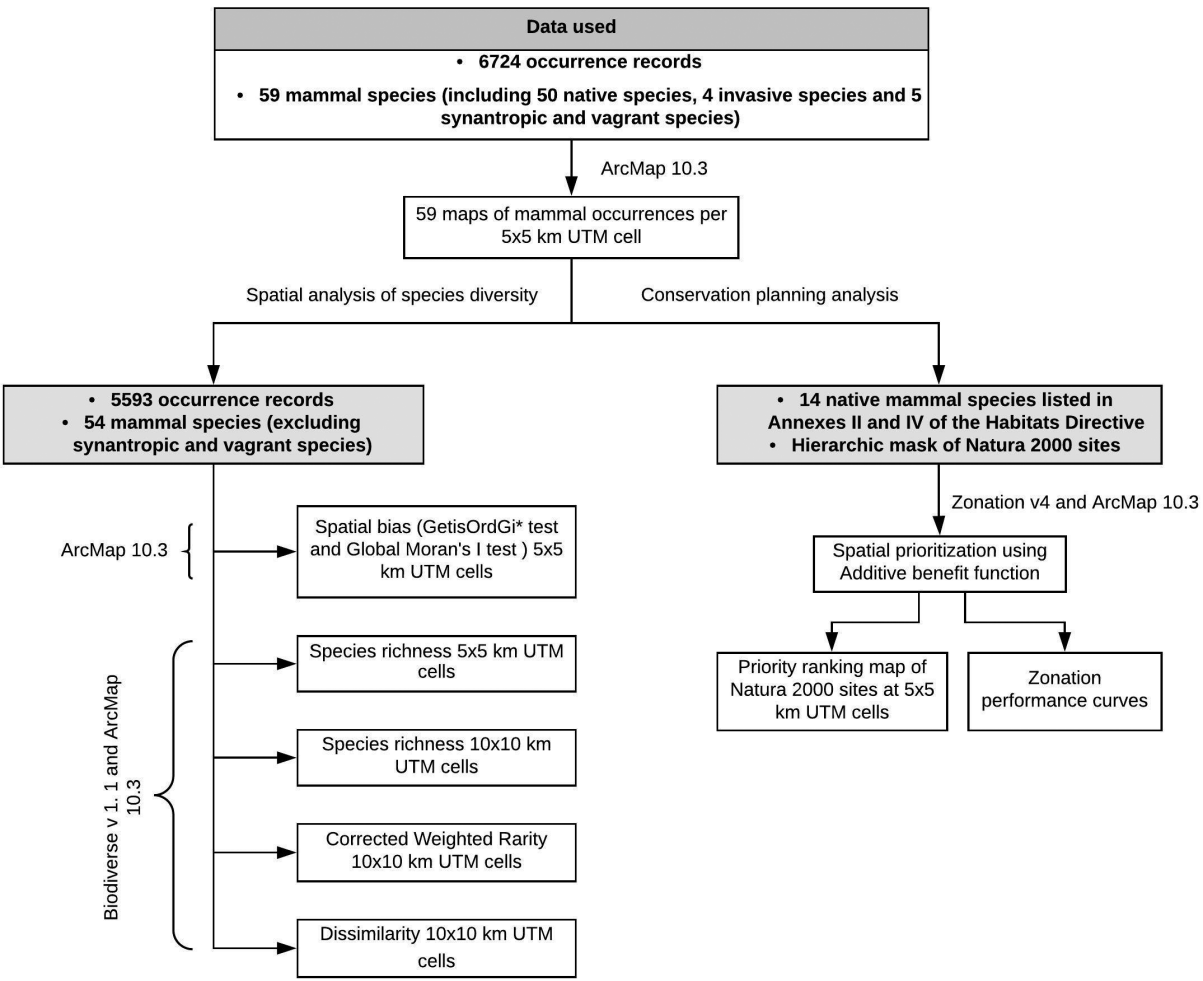
Flowchart of diversity analyses and spatial prioritisation of conservation of terrestrial mammals within Dobrogea Region, Romania.

The dataset used to map the species distribution includes 6724 occurrence records for 59 mammal species. For spatial pattern analyses, we excluded species found exclusively in fenced areas (the European mouflon *Ovisariesmusimon*), the vagrant species (elk *Alcesalces*), and synanthropic species (the rats *Rattusrattus*, *Rattusnorvegicus* and the house mouse *Musmusculus* (Table 2)), resulting in 5593 occurrence records for 54 species. For creating species distribution maps, we aggregated the occurrence records at a Universal Traverse Mercator spatial resolution of 25 km^2^ (UTM 5 × 5 km). Following Cogalniceanu et al. (2013), the occurrence records were classified based on the year of observation into *old records*, if recorded before 1990, and *recent records*, if recorded after 1990. For spatial pattern analyses, we increased the cell size to UTM 10 × 10 km, allowing us to highlight regional patterns in richness, rarity, and dissimilarity, and to reduce the potential bias in sampling (Graham and Hijmans 2006). For spatial prioritisation of mammal conservation within Natura 2000 sites, we used the UTM 5 × 5 km occurrences maps of 14 native species listed in Annexes II and IV of Habitats Directive (Figure 1).

### Spatial bias in species occurrence

Potential bias at the scale of the study area was assessed using the overall spatial autocorrelation in mammal records per 5 × 5 km grid cell. We used Global Moran’s I test (Fortin and Dale 2005) to evaluate spatial pattern of sampling per grid cell being significantly clustered (Z > 0) or dispersed (Z < 0) across Dobrogea. To assess the local patterns of sampling bias we used the Getis Ord Gi* spatial statistic. This analysis identifies clusters of records with values numerically higher than expected by random chance within a specified searching distance (Ord and Getis 1995). The distance threshold for the aggregation patterns was set up to 7100 m to include the neighbouring eight grid cells for each UTM grid of interest. The Getis Ord Gi* test returns a Z-score for every cell, which, depending on the level of aggregation describes spatial clusters of high or low sampling effort. We identified clusters of UTM 5 × 5 km cells where the sampling effort was significantly higher (hotspots of occurrence, GiZScore > 1.87) or lower (cold spots of occurrence, GiZScore < 1.87). All spatial analyses were performed using ARCMAP 10.3 (ESRI, CA) (Figure 1).

### Estimating species richness, rarity, and dissimilarity

To emphasise regional patterns of richness, rarity, and dissimilarity of mammals of Dobrogea, we aggregated the occurrence records at 5 × 5 km and 10 × 10 km and imported them into BIODIVERSE software (v. 1.1) (Laffan et al. 2010), a tool for spatial analysis of biodiversity (Figure 1).

Richness index was measured as the number of species in each grid cell. Species rarity was assessed by dividing the corrected weighted rarity (CWE) by the total number of species in the respective cell, where CWE is (Equation 1).

*CWE = WE / Richness* (1)

Weighted rarity (WE) of a species represents the occurrence records of sample counts of the respective species divided by the number of occurrence records of all species in the dataset (Equation 2).


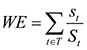
 (2)

where *t* is a taxon in the set of taxa *T* across neighbourhood set 1, *s_t_* is the sum of the sample counts for *t* across the elements in neighbouring sets 1 and 2, and *S_t_* represents the total number of samples across the data set for *t* (Laffan et al. 2010). In our case, only one neighbouring set is specified.

To calculate the differences in species composition across Dobrogea, we used the turnover index (*S_2_*), which refers to changes in species composition from one community to another along a gradient and across different sites (Whittaker 1972). *S_2_* calculates the dissimilarity between two sets of species. We compared a focal quadrat with one of its eight neighbours (Equation 3).

where *a* is the total number of species found in both neighbour sets, *b* is the number of species unique to the neighbour set 1, and *c* is the number of species unique to the neighbour set 2 (Laffan et al. 2010).


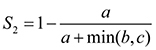
 (3)

Selecting the smallest values of *b* or *c* in the *S_2_* equation denominator reduces the impact of imbalances of species richness on neighbour dissimilarity. The highest value that *S_2_* can result is the value of one (1), which indicates the focal quadrat has no species in common with any neighbour and the lowest possible value is zero (0), indicating that all quadrats have an identical set of species (Lennon et al. 2001).

### Identifying high-priority areas for Natura 2000 mammal species conservation

To identify high-priority areas for mammal species conservation across Natura 2000 sites within Dobrogea Region, we used systematic conservation planning software ZONATION v4 (Lehtomäki and Moilanen 2013, Moilanen et al. 2014). This software uses a complementarity-based algorithm including connectivity, with the result that landscapes can be zoned according to their conservation potential. Using a deterministic iterative process, ZONATION creates a hierarchical ranking of the landscape from the highest to the lowest conservation value (Moilanen et al. 2014).

For priority analysis, we used 5 × 5 km raster layers for presence/absence data for 14 mammal species listed in Annexes II and IV of the Habitats Directive and a hierarchic mask of the Natura 2000 Sites of Community Importance within Dobrogea Region (Figure 1). A hierarchic mask represents a mask layer specifying priority land uses, in our case the Natura 2000 network. This planning design forces the prioritisation algorithm to undertake ranking cells outside the Natura 2000 network, followed by ranking those in the Natura 2000 network, allowing the application to analyse an optimal conservation area network. We sequenced the prioritisation model using an additive benefit function with exponent *z* = 0.25, which is a default value representing the exponent of the species-area curve (Moilanen et al. 2014). In this prioritisation model, the function sums the loss across features, converted via feature-specific benefit functions, giving high importance to the cells containing many species (Arponen et al. 2005).

The outputs of the analysis are conservation priority ranking of the landscape, derived from the order of iterative cell ranking whereby each grid cell has a value between 0 and 1, indicating that ranking close to 0 are removed first (low priority), while ranking close to 1 are retained until the end of the iteration. The outputs show the most important areas for mammal species conservation across Natura 2000 sites and a set of curves describing the absolute performance levels of species conservation. We considered as high-priority areas for conservation, all grid cells falling in the top 20% of the predicted priority ranks, a proportion that maximises mammal species representation at the regional level (Arponen et al. 2005). Suppl. material 3 presents the methodology used to identify high-priority Natura 2000 sites with Zonation v4.

The data underpinning the analysis reported in this paper are deposited at GBIF, the Global Biodiversity Information Facility, http://ipt.pensoft.net/resource?r=mammalsdobrogea.

## Results

### Mammal species occurrences in Dobrogea

We collected 4451 records from published museum collections data (66%), 1326 personal records shared by experts (20%), and 947 records from other papers reporting the results of fauna inventories (14%). Of all the accessible papers (published museum collections and fauna inventories) 67% were published before the year 1990 and 33% after 1990 (Suppl. material 1). Over 54% of all the records were reported before 1990, and 46% are records collected after 1990. Occurrences maps for 59 mammal species aggregated at 5 × 5 km resolution are presented in Suppl. material 2.

**Figure 2.**
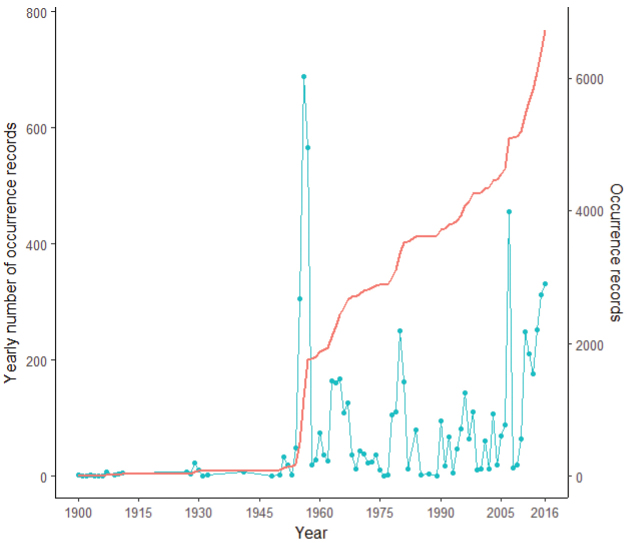
Accumulation of mammals’ occurrence records (blue) and the number of records per year (red) within Dobrogea Region, Romania.

The rate of accumulation of mammal occurrences increased in 1956 by 688 records, due to the rediscovery of the Southern birch mouse (*Sicistanordmanni*) at Valu lui Traian in 1955. That report attracted additional fieldwork by mammologists the following year, consequently, an increase in the number of records for other rodent species. After 1990, and up to 2017, the peak number of records per year took place in 2007 with 456 new records (Figure 2, Table 1).

**Table 1.** Checklist of mammals of Dobrogea Region, Romania.

Order	Family	Species	Total number of records	New records (after 1990)	Total number of UTM 5 × 5 occupied cells	Habitats Directive Annexes	European Red List status
Rodentia	Sciuridae	*Sciurusvulgaris* (Linnaeus, 1758)	6	3	4	–	Least concern
		*Spermophiluscitellus* (Linnaeus, 1766)	214	92	95	II/IV	Vulnerable
	Gliridae	*Dryomysnitedula* (Pallas, 1778)	35	22	20	IV	Least concern
		*Muscardinusavellanarius* (Linnaeus, 1758)	1	1	1	–	Least concern
		*Glisglis* (Linnaeus, 1766)	3	3	3	–	Least concern
	Castoridae	*Castorfiber* (Linnaeus, 1758)	12	12	5	II/IV	Least concern
	Dipodidae	*Sicistanordmanni* (Keyserling & Blasius, 1840)	76	0	2	II/IV	Vulnerable
	Spalacidae	*Nannospalaxleucodon* (Nordmann, 1840)	163	82	57	–	Least concern
	Cricetidae	*Cricetuscricetus* (Linnaeus, 1758)	2	0	1	IV	Least concern
		*Mesocricetusnewtoni* (Nehring, 1898)	98	13	31	II/IV	Near threatened
		*Ondatrazibethicus* (Linnaeus, 1766)	87	37	57	–	Invasive
		*Arvicolaamphibius* (Linnaeus, 1758)	29	12	22	–	Least concern
		*Microtusagrestis* (Linnaeus, 1761)	28	11	18	–	Least concern
		*Microtusarvalis* (Pallas, 1779)	187	40	44	–	Least concern
		*Microtuslevis* (Miller, 1908)	29	9	13	–	Least concern
		*Microtussubterraneus* (Selys-Longchamps, 1836)	16	7	9	–	Least concern
		*Myodesglareolus* (Schreber, 1780)	1	0	1	–	Least concern
	Muridae	*Micromysminutus* (Pallas, 1771)	36	15	24	–	Least concern
		*Apodemusagrarius* (Pallas, 1771)	451	96	50	–	Least concern
		*Apodemusflavicollis* (Melchior, 1834)	134	80	34	–	Least concern
		*Apodemussylvaticus* (Linnaeus, 1758)	1327	330	65	–	Least concern
		*Apodemusuralensis* (Pallas, 1811)	16	6	8	–	Least concern
		*Musspicilegus* (Petényi, 1882)	20	20	19	–	Least concern
	Myocastoridae	*Myocastorcoypus* (Molina, 1782)	5	2	5	–	Invasive
Lagomorpha	Leporidae	*Lepuseuropaeus* (Pallas, 1778)	262	255	102	–	Least concern
Erinaceomorpha	Erinaceidae	*Erinaceusroumanicus* (Barrett-Hamilton, 1900)	52	40	39	–	Least concern
Soricomorpha	Soricidae	*Crociduraleucodon* (Hermann, 1780)	85	14	18	–	Least concern
		*Crocidurasuaveolens* (Pallas, 1811)	131	40	36	–	Least concern
		*Neomysanomalus* (Cabrera, 1907)	12	4	8	–	Least concern
		*Neomysfodiens* (Pennant, 1771)	5	1	4	–	Least concern
		*Sorexaraneus* (Linnaeus, 1758)	63	14	25	–	Least concern
		*Sorexminutus* (Linnaeus, 1766)	15	9	10	–	Least concern
	Talpidae	*Talpaeuropaea* (Linnaeus, 1758)	65	53	55	–	Least concern
Carnivora	Felidae	*Felissilvestris* (Schreber, 1777)	101	94	52	IV	Least concern
	Felidae	*Lynxlynx* (Linnaeus, 1758)	2	1	2	II/IV	Least concern
	Canidae	*Canisaureus* (Linnaeus, 1758)	214	198	94	–	Least concern
		*Canislupus* (Linnaeus, 1758)	27	22	14	II/IV	Least concern
		*Nyctereutesprocyonoides* (Gray, 1834)	87	35	41	–	Invasive
		*Vulpesvulpes* (Linnaeus, 1758)	230	223	122	–	Least concern
	Mustelidae	*Mustelaerminea* (Linnaeus, 1758)	25	7	23	IV	Vulnerable
		*Mustelaeversmanii* (Lesson, 1827)	31	24	25	II/IV	Vulnerable
		*Mustelalutreola* (Linnaeus, 1761)	119	109	50	II/IV	Endangered
		*Mustelanivalis* (Linnaeus, 1766)	67	54	50	–	Least concern
		*Mustelaputorius* (Linnaeus, 1758)	89	74	61	–	Least concern
		*Vormelaperegusna* (Güldenstädt, 1770)	70	16	39	II/IV	Vulnerable
		*Martesfoina* (Erxleben, 1777)	98	97	58	–	Least concern
		*Martesmartes* (Linnaeus, 1758)	36	36	20	–	Least concern
		*Melesmeles* (Linnaeus, 1758)	102	92	60	–	Least concern
		*Neovisonvison* (Schreber, 1777)	2	2	1	–	Invasive
		*Lutralutra* (Linnaeus, 1758)	55	49	35	II/IV	Near threatened
Artiodactyla	Suidae	*Susscrofa* (Linnaeus, 1758)	221	204	105	–	Least concern
	Cervidae	*Damadama* (Linnaeus, 1758)	46	29	21	–	Least concern
		*Cervuselaphus* (Linnaeus, 1758)	38	32	25	–	Least concern
		*Capreoluscapreolus* (Linnaeus, 1758)	262	190	119	–	Least concern

### Spatial patterns in mammal species occurrences in Dobrogea

**Figure 3.**
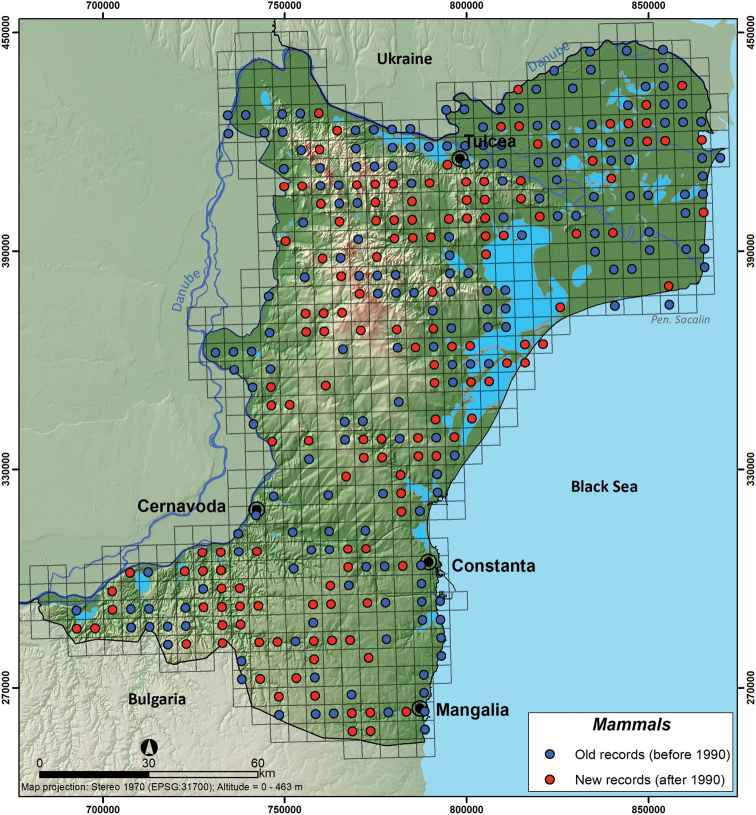
Mammals reported occurrences in Dobrogea Region, Romania at 5 × 5 km resolution. Grids with reported occurrences before 1990 were plotted as old records whereas those with reported occurrences after 1990 were considered new records (reports of synanthropic and vagrant mammals were excluded).

**Figure 4.**
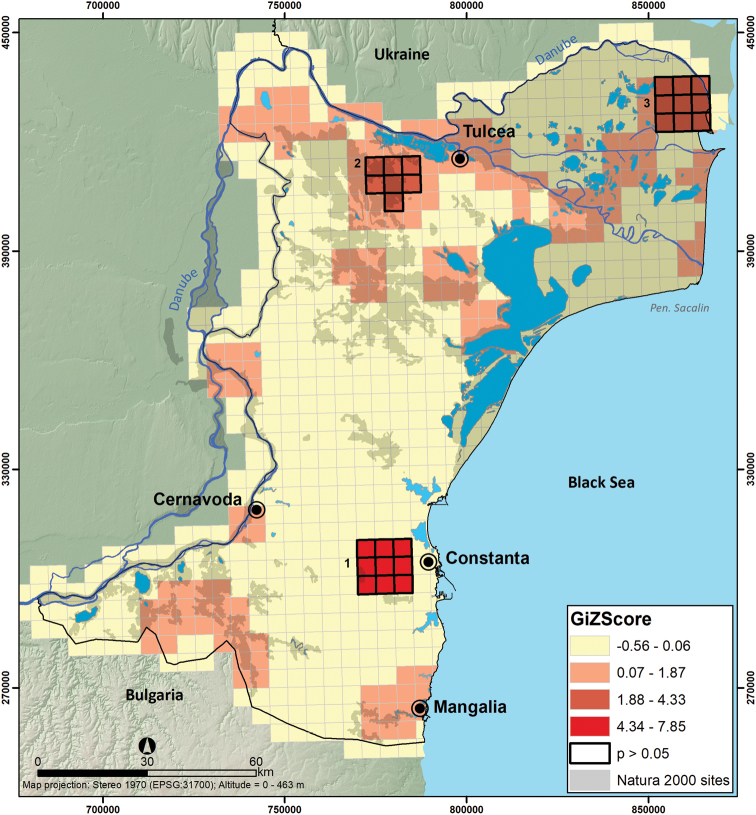
Hotspots of sampling efforts within Dobrogea. The numbered statistically significant hot-spots are **1** Valu lui Traian Biological Research Station and Fântânița-Murfatlar **2** North Dobrogea Plateau **3** Letea Forest Natural Reserve in the Danube Delta.

Of 757 UTM 5 × 5 km grid cells encompassing the Dobrogea Region, only 335 grid cells (i.e., 44%) include reported mammal sightings (Figure 3). At the regional scale, Global Moran’s I test indicated a random pattern in the number of mammal occurrences per UTM 5 × 5 grid cell (Z = 1.87, p = 0.06). However, the local Getis Ord Gi* spatial statistic indicates 3 hotspots for mammal sightings: Valu lui Traian Biological Research Station (mean Z = 7.73), North Dobrogea Plateau Natura 2000 site (mean Z = 3.26), and Letea Forest, a natural reserve within Danube Delta (mean Z = 2.75). Additionally, there are few moderately sampled regions such as Măcin Mountains National Park in the northwest, Dumbrăveni-Urluia Valley-Vederoasa Lake Natura 2000 site and Canaraua-Fetii Iortmac Natura 2000 site in the southwest, and Hagieni – Cotul Văii Forest Natura 2000 site in the southeast (Figure 4).

**Figure 5.**
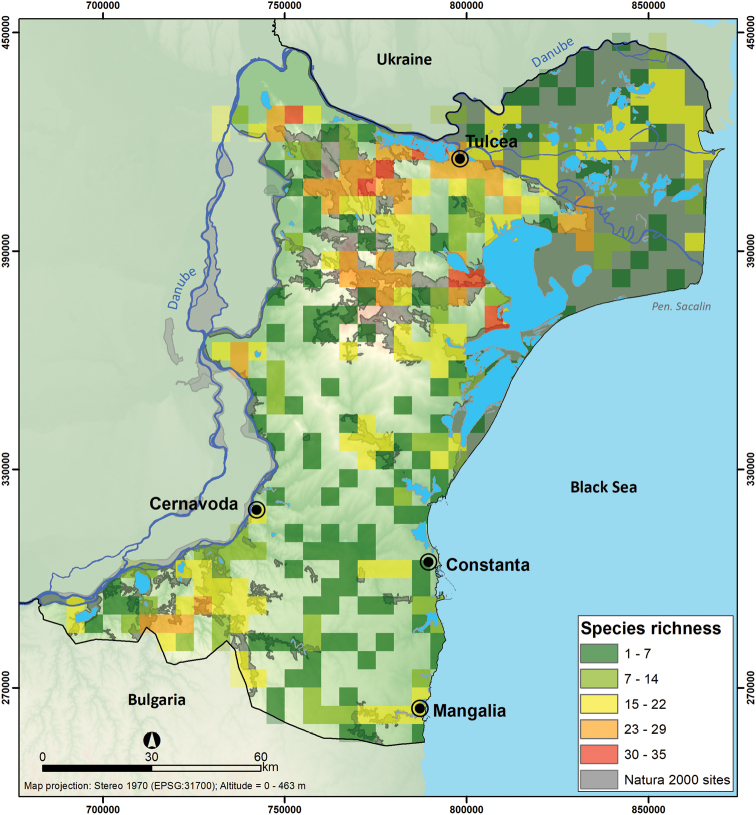
The mammal species richness at 5 × 5 km grid resolution within Dobrogea.

The mammal occurrences at 5 × 5 km resolution ranged between 1 and 35 reported species per quadrat (Figure 5). The map highlights a lower sampling effort in southern and central Dobrogea, areas with intensive agriculture, and the highest diversity in the northern and southwest parts of Dobrogea, comprising mostly forested habitat.

**Table 2.** Checklist of synanthropic and vagrant mammals of Dobrogea Region, Romania.

Order	Family	Species	Total number of records	New records (after 1990)	Total number of UTM 5 × 5 occupied cells
Rodentia	Muridae	*Rattusnorvegicus* (Berkenhout, 1769)	114	49	64
Rodentia	Muridae	*Rattusrattus* (Linnaeus, 1758)	3	2	3
Rodentia	Muridae	*Musmusculus* (Linnaeus, 1758)	1001	139	78
Artiodactyla	Cervidae	*Alcesalces* (Linnaeus, 1758)	4	0	3
Artiodactyla	Bovidae	*Ovisariesmusimon* (Pallas, 1881)	9	5	4

### Species richness, rarity, and dissimilarity

**Figure 6.**
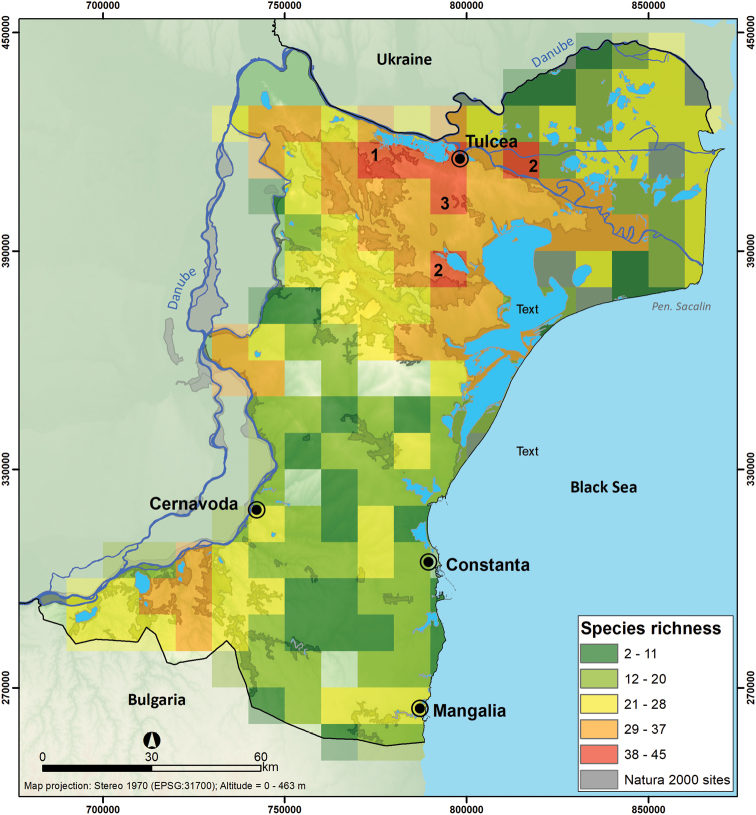
Mammal species richness of Dobrogea at 10 × 10 km. Grids with high richness partially overlap. **1** North Dobrogea Plateau **2** Danube Delta, and **3** Agighiolului Hills.

When aggregating species records at 10 × 10 km, the number of reported species ranged from 2 to 45 per cell grid, with the highest species diversity located in the northern part of Dobrogea Region overlapping the following Natura 2000 sites: North Dobrogea Plateau with a maximum richness of 45 species, western part of Danube Delta with 39 species and Agighiolului Hills with 38 species. Most of the grid cells with species richness are concentrated in the northern region reflecting an optimal sampling of mammal species (number of species from 29 to 37) (Figure 6), while grid cells with the lowest richness values are distributed in the southern and central part of Dobrogea Region.

**Figure 7.**
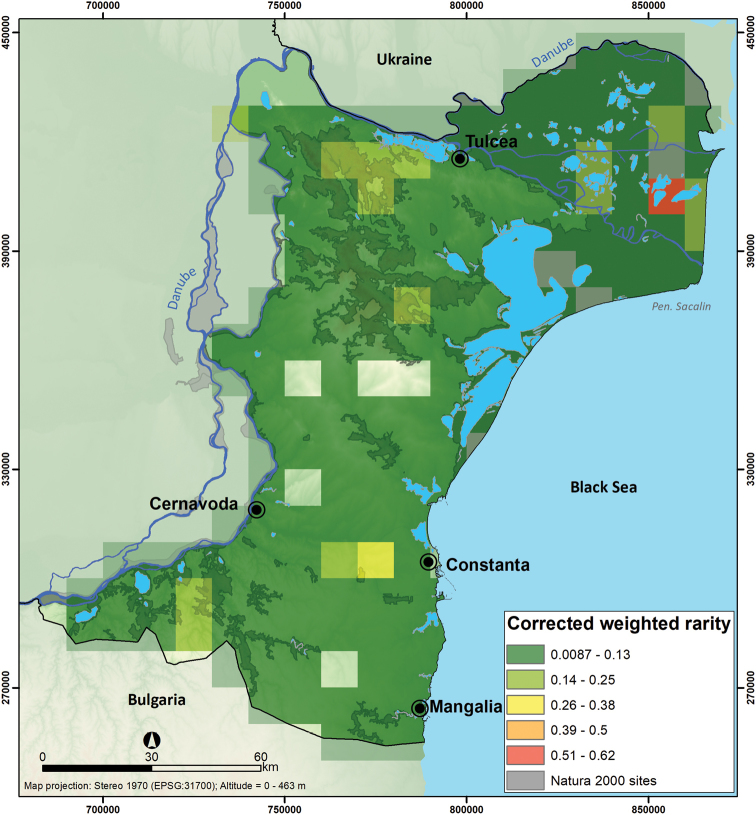
Corrected weighted rarity map of Dobrogea mammal species.

Corrected weighted rarity (CWE) varied across Dobrogea from 0.0087 for cell grids with widespread species to 0.62 grid cells with species of restricted distribution. The highest value of corrected weighted rarity can be found in the Danube Delta, specifically in the levee complex of Puiu – Roșu – Lumina, with a value up to 0.62 (Figure 7).

**Figure 8.**
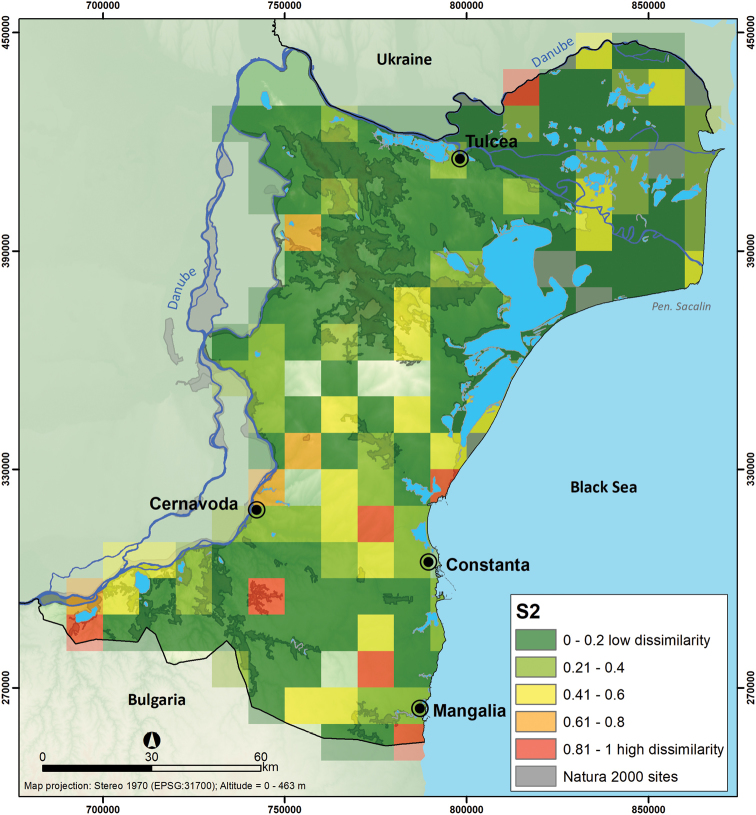
Dissimilarity map of Dobrogea mammal species, Romania (S2 index).

The values of dissimilarity index S2 ranged from 0 to 1 with the highest turnover quadrats in the southern area of Dobrogea where there are low richness zones. The value of 1 implies that the quadrat has no species in common with any neighbour (Figure 8). We found that areas with the higher richness of species have more species in common with their neighbours.

### High-priority areas for conservation within Natura 2000 sites

**Figure 9.**
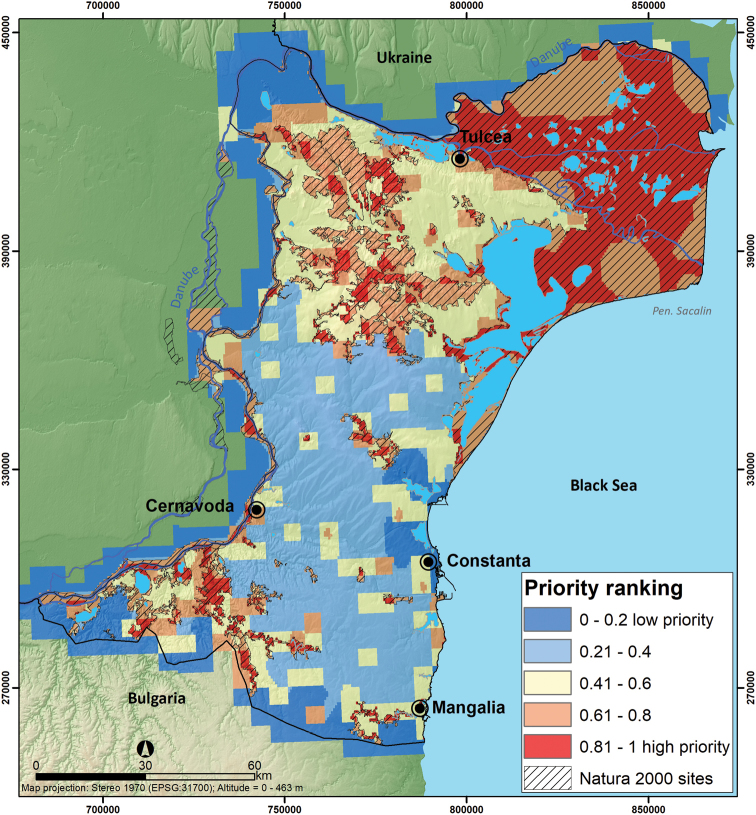
Priority conservation areas for mammal species listed in Annexes II and IV of the Habitats Directive within Natura 2000 sites of Dobrogea. Areas have been graded according to their priority rank, with highest priorities (top 20%) shown in red.

**Figure 10.**
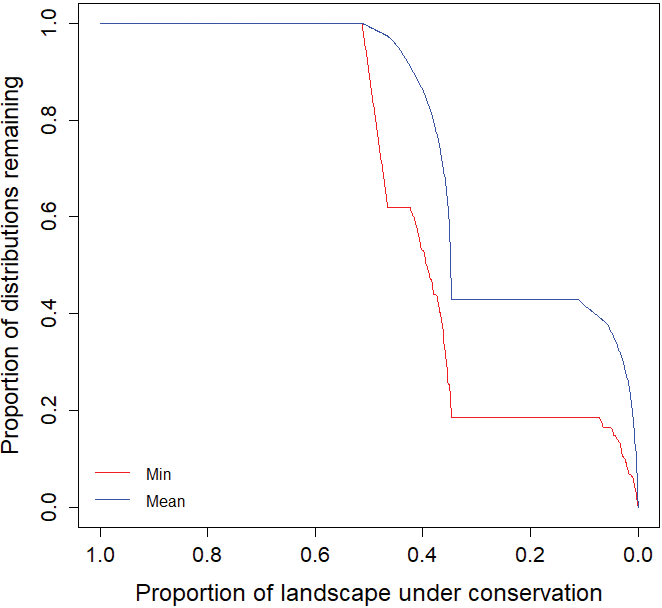
Zonation performance curves quantifying the proportion of remaining species occurrences covered by Natura 2000 sites in Dobrogea. When considering 20% of landscape within Natura 2000 sites as protected (e.g., conservation measures are enforced), 45% of Natura 2000 mammal occurrences are protected.

Based on the Zonation analysis results, the top spatial conservation priorities overlap Danube Delta, North Dobrogea Plateau, and the Măcin Mountains in the northern part of Dobrogea region, where a relatively aggregated pattern of top conservation value areas appear due to their extensive wetland area and forested habitats. Isolated hotspots are represented by Dumbrăveni-Urluia Valley-Vederoasa Lake in the southwest, Hagieni – Cotul Văii Forest in the southeast, and Cheia Jurassic Reefs in Central Dobrogea. Grid cells with the lowest ranking are located in the central and southern part of Dobrogea Region, where the majority of the regions’ agricultural lands are clustered (Figure 9). Nevertheless, the Natura 2000 network encompasses 45% of mammal species distribution listed in Annexes II and IV of the Habitats Directive when top 20% of the landscape is protected by Natura 2000 sites (Figure 10).

## Discussion

By using an updated distribution of terrestrial mammals, we identified high priority areas for protecting mammal diversity to guide future conservation efforts in an extensively protected Romanian region. In the broader context of systematic conservation planning, the prioritisation analysis is a useful tool to identify key areas for biodiversity conservation, e.g., where species are more likely to survive (Ferrier and Wintle 2009, Wilson et al. 2009, Kukkala and Moilanen 2013).

The number of reported occurrences in Dobrogea varied among species. The largest number of records (20%) are for the wood mouse (*Apodemussylvaticus*), mostly because they are widespread within the region, have a higher population abundance, and are evidently. The wood mouse may be easily misidentified as a yellow-necked mouse (*Apodemusflavicollis*) (Bartolommei et al. 2016), but it still retains the first rank because of their higher population in Dobrogea (Popescu and Murariu 2001).

The lowest number of records in Dobrogea is recorded for the hazel dormouse (*Muscardinusavellanarius*) and the bank vole (*Myodesglareolus*), with only one record per each species. Two other widespread species, but with an uncharacteristically low number of reported presences are the red fox (*Vulpesvulpes*) and the roe deer (*Capreoluscapreolus*), possibly because they are common species, with minor interest for biologists. The research effort for species sampling was focused on selected areas: Măcin Mountains National Park, Danube Delta Biosphere Reserve, and the North Dobrogea Plateau. Here, we recorded a higher than expected number of species occurrences per grid cell, mostly because the long-term protection status attracted faunistic inventory projects over time. Typically, the sampling bias is higher in protected areas because they attract more conservation funds leading to greater efforts for biodiversity research (e.g., Botts et al. 2011). This process describes most of the biodiversity spatial databases (Lobo et al. 2007). A higher than expected sampling effort also was evident near cities, major roads, and research facilities, which are easily accessible to researchers (e.g., Valu lui Traian Biological Research Station - see Figure 4). We noted a lack of research interest in central and southern Dobrogea, where most of the agricultural landscape is located, and only small patches of natural habitats remain as wildlife refuges (Rey et al. 2007). However, agricultural landscapes are essential for many species protected by Habitats Directive, such as *Vormelaperegusna*, *Mustelaeversmanii*, *Spermophiluscitellus*, *Mesocricetusnewtoni*, and *Cricetuscricetus* (Popescu and Murariu 2001, Murariu and Munteanu 2005, Murariu et al. 2009, 2010). Those identified species have restrictive ecological requirements, and hence, the researchers should focus on increasing sampling effort in these neglected areas to draft appropriate conservation plans.

We found that high species richness did not match all the hotspots of sampling efforts, such as in Valu lui Traian Biological Research Station and Fântânița-Murfatlar (location 1 in Figure 4) and Letea Forest Natural Reserve in the Danube Delta (location 3 in Figure 4). This validates the finding that intensive sampling was not directly correlated with mammal diversity, but rather ease of access to the regions (Santos et al. 2017). To better understand the patterns of species richness at the regional level, we expanded the resolution to 10 × 10 km, but the spatial pattern did not change between the two mapping resolutions. In both cases, the highest diversity (35 species at 5 × 5 km resolution, see Figure 5); 45 mammal species at 10 × 10 km resolution, see Figure 6), is found in the northern part of Dobrogea, overlapping North Dobrogea Plateau Natura 2000 site, where habitat heterogeneity is high (Rey et al. 2007).

The spatial turnover index (S_2_) suggests that areas with lower species richness are dissimilar compared to their neighbours. Notably, we observed some affinities of particular species towards low species richness areas (Lennon et al. 2001), e.g., species dependent on steppe or agricultural landscape (Popescu and Murariu 2001, Murariu and Munteanu 2005). Typically, the spatial turnover tends to be correlated with species richness (Gaston et al. 2007), but in our study, the variation in turnover is determined by the rarity of the species which then tend to have narrower habitat niches and drive turnover patterns more than widespread species. By analysing species richness and turnover index maps, we found low congruency between the Natura 2000 sites and areas with high species richness and areas occupied by species with a narrower range. Species with narrow ranges (e.g. *Lutralutra*, Memedemin et al. 2017) are often underrepresented in protected areas, potentially resulting in suboptimal effectiveness of the Natura 2000 network in protecting such species in Dobrogea, despite the large area protected under conservation.

Our results regarding the identification of high-priority areas in Dobrogea for mammal species listed in Annexes II and IV of the Habitats Directive highlight a relatively aggregated pattern of the grid cells with high conservation value in the northeastern and northern Dobrogea Region, where there are extensive wetlands and forests (i.e., Danube Delta, Măcin Mountains National Park, and North Dobrogea Plateau) (Rey et al. 2007). Additionally, we identified small isolated high-priority areas in agricultural landscapes of southern and central Dobrogea, where biodiversity-friendly agricultural practices should be considered as a conservation method. Distribution of top spatial conservation priorities demonstrated a lack of connectivity between Natura 2000 sites with high conservation values from the northern part of Dobrogea, isolated priority areas in the center of the region, as well as those in the southern part of the region. The distribution of high-priority areas for conservation suggests the necessity of addressing the lack of connectivity, as non-priority areas are essential for the dispersal of species (Christie and Knowles 2015).

The significant overlap between Natura 2000 sites and the other protected areas statutes leads to misunderstandings in law enforcement and an over-optimistic vision of their effectiveness (Ioja et al. 2010). As an example, species whose distributions are limited to the EU Steppic Biogeographic Region or reach the boundaries of their geographic range in Dobrogea tend to be under-represented (Popescu and Murariu 2001, Murariu and Munteanu 2005) as in the case of reptiles and amphibians (Popescu et al. 2013). In Dobrogea, isolation of protected areas leads to low connectivity between habitat patches, which then need to be addressed in future conservation planning and protected area management plans. The lack of research in agricultural landscapes may potentially lead to the populational decline of certain species by not being aware of their distribution and by using flawed species range data (Grant et al. 2007).

The absence of buffer zones and corridors between Natura 2000 sites and small isolated protected areas (the area of the smallest Natura 2000 site in Dobrogea is 0.11 km^2^), are not beneficial in maintaining viable populations, causing the isolation of species with low mobility and specific habitat requirements (Christie and Knowles 2015). Establishing corridors between Natura 2000 sites, especially in the central and southern part of Dobrogea increases connectivity and promotes species dispersal.

Our study is limited by the lack of viable and current distribution data. Most records do not identify geographical coordinates, but localities or toponymies. This makes the niche modelling at a fine scale a challenge. Furthermore, elusive species such as *Mesocricetusnewtoni*, *Sicistanordmanni*, and *Vormelaperegusna*, are data deficient, and the lack of records (false absences) may influence the results of the analysis. Similarly, misidentification of sibling species may lead to over- or under- estimation of their range. Notably, a study analysing the distribution of amphibians in Dobrogea (Székely et al. 2009) indicates similar issues regarding biased and incomplete distribution data due to the lack of comprehensive surveys of areas with difficult accesses. Another similarity is that some amphibians (e.g., *Bombinabombina*, *Bufoviridis*, *Hylaarborea*), as well as some mammals (e.g., *Capreoluscapreolus*, *Apodemussylvaticus*) are considered widespread and highly detectable, while amphibians such as *Pelobatesfuscus* and *Pelobatessyriacus*, are cryptic and elusive species and therefore, have low detectability and incomplete distributions (Székely et al. 2013), and that includes species such as *Vormelaperegusna*, *Sicistanordmanni* or *Mesocricetusnewtoni*. However, biased data lead to more priority areas to protect fewer species (Grant et al. 2007), which is not a shortcoming. Furthermore, Rodrigues et al. (2011) concluded that decision-based on incomplete taxonomic and/or phylogenetic data (such as misidentified sibling species) are robust, and the researcher can safely make use of the best available systematic data.

Future research may focus on identifying buffer zones around Natura 2000 sites to minimise potential negative impacts, particularly in Natura 2000 sites that are adjacent to agricultural areas. From this assessment, we envisage further mapping of corridor networks between small isolated protected areas in southern and central Dobrogea. New research should focus on systematic surveys of agricultural landscapes in central and southern Dobrogea, where vegetation patches remain as refugees for some species listed in Annexes II and IV of the Habitats Directive (*Vormelaperegusna*, *Mustelaeversmanii*, *Spermophiluscitellus*, *Mesocricetusnewtoni*, *Cricetuscricetus*, and *Sicistanordmanni*).

## Supplementary materials

Supplementary material 1 Publications used to compile distribution of terrestrial mammal species from Dobrogea Region, Romania (field reports and data from museum collections) Data type: reference This is an open access article distributed under the terms of the Creative Commons Attribution License (CC BY 4.0), which permits unrestricted use, distribution, and reproduction in any medium, provided the original author and source are credited.Iulia V. Miu, Gabriel B. Chisamera, Viorel D. Popescu, Ruben Iosif, Andreea Nita, Steluta Manolache, Viorel D. Gavril, Ioana Cobzaru, Laurentiu Rozylowicz

Supplementary material 2 Occurrences maps for 59 mammal species Data type: occurence Explanation note: The mammal species old (before 1990) and new (after 1990) occurrence records at a 5 × 5 km grid resolution within Dobrogea, Romania. This is an open access article distributed under the terms of the Creative Commons Attribution License (CC BY 4.0), which permits unrestricted use, distribution, and reproduction in any medium, provided the original author and source are credited.Iulia V. Miu, Gabriel B. Chisamera, Viorel D. Popescu, Ruben Iosif, Andreea Nita, Steluta Manolache, Viorel D. Gavril, Ioana Cobzaru, Laurentiu Rozylowicz

Supplementary material 3 Methodology used to identify high-priority Natura 2000 sites with Zonation v4 Data type: methodology This is an open access article distributed under the terms of the Creative Commons Attribution License (CC BY 4.0), which permits unrestricted use, distribution, and reproduction in any medium, provided the original author and source are credited.Iulia V. Miu, Gabriel B. Chisamera, Viorel D. Popescu, Ruben Iosif, Andreea Nita, Steluta Manolache, Viorel D. Gavril, Ioana Cobzaru, Laurentiu Rozylowicz
