# Supplementary material for: Conservation priorities for terrestrial mammals in Dobrogea Region, Romania
Source: Zookeys. 2018 Oct 23;(792):133–58. doi: 10.3897/zookeys.792.25314 (PMC6215976; doi:10.3897/zookeys.792.25314)
Supplement: Supplementary material 1 — Publications used to compile distribution of terrestrial mammal species from Dobrogea Region, Romania (field reports and data from museum collections) [file zookeys-792-133-s001.pdf]

Iulia V. Miu, Gabriel B. Chișamera, Viorel D. Popescu, Ruben Iosif, Andreea Nita, Steluta Manolache, Viorel D. Gavril, Ioana Cobzaru, Laurentiu Rozylowicz (2018) Conservation priorities for terrestrial mammals in Dobrogea Region, Romania. Zookeys

Appendix 1

Publications used to compile distribution of terrestrial mammal species from Dobrogea region, Romania (field reports and data from museum collections)

1. Almăşan H, Andone G, Nesterov V (1959) Nutria în Delta Dunării. Vânătorul şi Pescarul Sportiv 5: 10.
2. Andone G (1959) Bizamul (*Ondatra zibethica* L.), în Delta Dunării. Revista Pădurilor 2:100–104.
3. Angelescu A (2004) Șacalul Auriu. Origine. Morfoanatomie. Eco-etologie. Management. MMC. București.
4. Atanasov N (1966) *Vormela peregusna* (Güld, 1770) in Bulgarien und auf der Balkanhalbinzel. Zeitschrift für Säugetierkunde 6: 453–464.
5. Ausländer D, Hamar M, Hellwing S, Schnapp B (1958) Zur Systematik und Verbreitung der Streifenmaus (*Sicista subtilis nordmani* Keys et Blas, 1840). Zeitschrift für Säugetierkunde 24: 68–77.
6. Ausländer D, Hellwing S (1957) Observations écologiques sur les petits mammifères des ecrans forestiers de protéction de “Valul Traian”; références spéciales concernant leur dynamique. Travaux du Muséum d’Histoire Naturelle Grigore Antipa 1: 111–139.
7. Ausländer D, Hellwing S (1957) Beiträge zur Variabilität und Biologie der Streifenmaus (*Sicista subtilis* Nordmanni Keys et Blas., 1940). Travaux du Muséum d’Histoire Naturelle Grigore Antipa 1: 255-274.
8. Barbu I (1971) Dobrogea cinegetică. Vânătorul şi Pescarul Sportiv 1: 19–20.
9. Barbu P (1967) Aspecte din ecologia câinelui enot în Delta Dunării. Vânătorul şi Pescarul Sportiv 3: 10–11.
10. Barbu P (1968) La nourriture du nyctéréute *(Nyctereutes procyonoides* Gray) du Delta du Danube. Revue Roumaine de Biologie, Série Zoologie 5: 301–306.
11. Barbu P (1969) Noi contribuții la cunoaşterea ecologiei cîinelui enot *Nyctereutes procyonoides ussuriensis* Matschie, 1907 din Delta Dunării. Studii şi Cercetări de Biologie, Seria Zoologie 1: 103–115.
12. Barbu P (1970) Sur la reproduction du nyctéréute (*Nyctereutes procyonoides ussuriensis* Matschie, 1907) dans le Delta du Danube, Travaux du Muséum d’Histoire Naturelle Grigore Antipa 10: 331-345.
13. Barbu P (1971) Şorecarul încălţat (*Buteo lagopus lagopus* Pont.), pasăre folositoare. Ocrotirea Naturii 1: 61–66.

14. Barbu P, Popescu A (1965) Variaţia hranei la *Asio otus otus* (L.) din pădurea Comarova (reg. Dobrogea) stabilită cu ajutorul ingluviilor. Studii şi Cercetări de Biologie, Seria Zoologie 2: 187-195.

15. Bodea M (1957) Din viața Deltei Dunării. Asociația Generală a Vânătorilor și Pescarilor Sportivi din Republica Populară Română.

16. Bunescu A (1959) Contribution à l'étude de la répartition geographique de quelques mammifères méditerranéene en Roumanie. Säugerierkundliche Mitteilungen 1: 1–4.

17. Bunescu A (1961) Contribuţii la studiul răspândirii geografice a unor animale mediteraneene din Republica Populară Română, Nota II – Vertebrate. Probleme de Geografie 8: 123–144.

18. Capotă V (1964) Rozătoarele (Rodentia) din Nordul Dobrogei. Teză doctorat. București. România. Universitatea București.

19. Călinescu R (1929) *Mustela (Putorius) eversmani* Lesson în România. Întâiul Congres al Naturaliştilor din România. Dare de Seamă a Lucrărilor, Cluj: 156–158.

20. Călinescu R (1931) Contribuţiuni sistematice, zoogeografice la studiul Mustelidelor din România. Lucrările Institutului de Geografie, Universitatea Cluj 4: 69–71.

21. Călinescu R (1933) Dihorul de stepă şi distribuţia sa geografică în România. Buletinul Societăţii Regale Române de Geografie 51: 114.

22. Călinescu R (1934) Taxonomische, biologische und biogeographische Forschungen über die Gattung *Citellus* Oken in Rumänien. Zeitschrift für Säugetierkunde 9: 87–141.

23. Călinescu R (1934) Les mammifères de la Dobrogea et surtout celles du littoral de la Mer Noire. Annales Scientifiques de l'Université de Jassy 1–4: 373–377.

24. Călinescu R (1956) Sciuridele din Republica Populară Română. Editura Ştiinţifică, Bucureşti.

25. Călinescu R, Bunescu A (1958) Răspândirea geografică a bizamului (*Ondatra zibethica* L.), în Republica Populară Română. Buletinul Institutului de Cercetări Piscicole 2: 78–82.

26. Chiriac E, Șutova M (1977) Recherches sur la parasitofaune de sousliks (*Citellus citellus* L.) à la Valu lui Traian (Dobrogea). Analele Universităţii Bucureşti, Seria Biologie. 26:99–102.

27. Chiriac E, Barbu, P (1962) Contribuţii la cunoaşterea helmintofaunei micromamiferelor din împrejurimile Măcinului (Dobrogea de Nord). Studii şi Cercetări de Biologie, Seria Biologie Animală 3: 385–392.

28. Chiriacescu N (1958) A existat cerbul în Dobrogea? Vânătorul şi Pescarul Sportiv 5: 19.

29. Cuzic M (2004) Contribuții la studiul mamiferelor și reptilelor din zona limanelor fluviatile Bugeac, Oltina, Dunăreni și Vederoasa. Delta Dunării. Studii și cercetări de științele naturii și muzeologie II: 167–174.

30. Cuzic M, Cuzic, V (2008) Date faunistice privind mamiferele din zona Lacului Furtuna din Delta Dunării. Acta Musei Tutovensis 8:224–229.

31. Cuzic M, Geru M, Poștaru N (2013) Catalogul sistematic al colecţiei de schelete parţiale de mamifere a Centrului Muzeal Ecoturistic „Delta Dunării”. Acta Musei Tutovensis 8: 211–223.

32. Cuzic M, Marinov M, Cuzic V (2002) American mink (*Mustela lutreola* in the Danube Delta, Scientific Annals of the „Danube Delta” Institut 9: 52-54.

33. Cuzic M, Marinov M (2002) Martes foina (Erxleben, 1777) Mammalia, Carnivora, in Dobrudja. Scientific Annals of the Danube Delta Institute for Research and Development: 55–59.

34. Cuzic M, Marinov M (2004) Date privind situația populației de nurcă europeană (*Mustela lutreola* L, 1761) (Mammalia, Carnivora, Mustelidae) în câteva zone din Rezervația Biosferei Delta Dunării. Muzeul Brukenthal – Studii și Comunicări – Științele Naturii 29: 231–239.

35. Dănilă, I (1982) La structure et la dynamique des populations de spermophile (*Citellus cittellus* L. 1766, Rodentia) de Roumanie, Travaux du Muséum d'Histoire Naturelle Grigore Antipa XXIV: 251-266.

36. Dogaru E (1979) Mamifere mici – insentivore și rozătoare – din Județul Constanța. Teză doctorat. București. România. Universitatea București.

37. Dogaru E (1986) Mamifere mici din județul Constanța: sistematică, biologie, importanță practică. Lucrare metodico-științifică pentru obținerea gradului didactic I. București. România. Universitatea București.

38. Filipaşcu A (1976) Omul şi fauna dobrogeană de-a lungul secolelor. Ocrotirea naturii dobrogene 29–39.

39. Geacu S (2011) Dinamica populațiilor de cervide și bovide din fauna României. Editura Academiei Române. București.

40. Hamar M, Schutowa M (1966) Neue daten über die geographische veränderlichkeit und die entwicklung der gattung *Mesocricetus* Nehring, 1898 (Glires, Mammalia). Z. Säugetierkunde 31: 237–251.

41. Hamar M, Tuţă A, Perju T (1965) Contribuţii la cunoaşterea biologiei şi combaterii şobolanului de apă (*Arvicola terrestris* L.) în România. Analele Institutului de Cercetări Agricole, Secţia de Protecţia Plantelor 3: 337–351.

42. Hellwing S, Schnapp B (1960) Populations-ökologische Forschungen an Kleinsäugern zu Valul lui Traian in den Jahren 1955–1957. Travaux du Muséum d'Histoire Naturelle Grigore Antipa 2: 337–378.

43. Homei V, Popescu A (1969) Contribuţii la studiul hranei de iarnă a ciufilor (*Asio o. otus* L.) din zona inundabilă a Dunării. Ocrotirea Naturii 1: 63–67.

44. Iana S (1970) Noutăţi faunistice în ecosistemele Dobrogei de Sud. Studii şi Comunicări. Ocrotirea Naturii Suceava: 17–23.

45. Ivanovici P (1959) Cerbii în Dobrogea. Vânătorul şi Pescarul Sportiv 1: 27.

46. Kiss JB (1974) Mamiferele sălbatice de pe Insula Sacalin. Natura 1:78–80.

47. Kiss JB, Doroșensu A, Marinov M, Alexe V (2013) Brebul (*Castor fiber*) în Delta Dunării: Prezent și perspective de viitor. Revista de Silvicultură și Cinegetică, Faună Sălbatică XVIII: 32.

48. Kiss JB, Doroșensu A, Sandor DA, Marinov M, Alexe V (2012) Răspândirea teritorială a jderului de piatră (*Martes foina*) în Dobrogea și apariția lui și în Delta Dunării. Revista de Silvicultură și Cinegetică, Faună Sălbatică XVII: 31.

49. Kiss JB, Marinov M, Alexe V, Doroșenco A (2014) Eurasian Beaver (*Castor fiber* L. 1758), Pine Marten (*Martes martes* L. 1758) and Stone Marten (*Martes foina* / Erxleben, 1777) in the Danube Delta (Romania). Beitrage zur Jagd-und Wildforschung 39: 347–355.

50. Kiss JB, Marinov M, Alexe V, Doroșenco A, Bozagevici R (2012) Consideration regarding the occurence of the Eurasian Beaver (*Castor fiber* Linnaeus 1758) in the Danube Delta (Romania). Scientific Annals of the Danube Delta Institute 18: 49–56.

51. Kiss JB, Marinov M, Alexe V, Doroșenco A, Sandor A (2013) Pătrunderea jderului de copac (*Martes martes*, Linnaeus 1758) în Delta Dunării (România) şi considerații privind urmările ecologice scontate al acestui fenomen. Revista Pădurilor 128: 3.

52. Kiss JB, Marinov M, Alexe V, Sandor A (2012) First record of the Pine Marten (*Martes martes*) in the Danube Delta. North-Western Journal of Zoology 8 (1):195–197.

53. Kiss JB (2004) Situația actuală a lupului (*Canis lupus* L.) în Delta Dunării. Delta Dunării. Studii și cercetări de științele naturii și muzeologie II: 175–182.

54. Kiss JB, Alexe V (2013) Un caz de albinism la șacalul auriu (*Canis aureus* Linnaeus 1758). Revista Pădurilor 128: 6.

55. Marcheş G (1956) Despre cîteva rozătoare din ţara noastră. Ocrotirea Naturii 2: 65-91.

56. Marcheş G (1960) Problema bizamului (*Ondatra zibethica* L.) în ţara noastră. Ocrotirea Naturii 5: 12-52.

57. Marcheş G (1970) Date privind răspândirea şi importanţa ştiinţifică şi practică a unor mamifere din Dobrogea. Ocrotirea Naturii 14: 165–180.

58. Marcheş G, Ausländer D, Hellwing S, Marcoci G, Schnapp B, Vâlceanu V (1954) Date preliminare cu privire la dinamica mamiferelor din perdelele forestiere de protecţie de la Valul lui Traian – (Dobrogea) şi Mărculeşti – (Bărăgan). Natura 5: 69–77.

59. Miller GS (1912) Catalogue of the Mammals of Western Europe (Europe exclusive of Russia) in the Collection of the British Museum. Longmans, Green & Co. London.

60. Marinov M (2011) Manual de management al nurcii europene (*Mustela lutreola*) în Rezervația Biosferei Delta Dunării – România. Danube Delta Technology Information, Tulcea, 53 pp.

61. Murariu D (1981) Contribution à la connaissance de la distribution et de l’écologie des mammifères de la zone du Delta du Danube et du Lac Razelm (Roumanie). Travaux du Muséum d’Histoire Naturelle Grigore Antipa 23: 283-296.

62. Murariu D (1981) La présence de *Mus musculus spicilegus* Petenyi, 1822 dans le Delta du Danube acconpagné de son „parasite” *Apodemus agrarius* (Pall., 1771). Travaux du Muséum d’Histoire Naturelle Grigore Antipa 23: 297-304.

63. Murariu D (1996) Mammals of the Danube Delta (Romania). Travaux du Museum National d’Histoire Naturelle Grigore Antipa 36: 361–371.

64. Murariu D (2006) Mammal ecology and distribution from North Dobrogea (Romania). Travaux du Muséum National d’Histoire Naturelle Grigore Antipa 49: 387–399.

65. Murariu D, Atanasova I, Raykov I, Chişamera G (2009) Results on mammal (Mammalia) survey from Bulgarian and Romanian Dobrogea. Travaux du Museum National d’Histoire Naturelle Grigore Antipa 52: 371–386.

66. Murariu D, Chişamera G, Petrescu A, Atanasova I, Raykov I (2010) Terrestrial vertebrates of Dobrogea - Romania and Bulgaria. Travaux du Muséum National d’Histoire Naturelle Grigore Antipa 53: 357–375.

67. Murariu D, Munteanu D (2005) Fauna României. Mammalia. Carnivora. Editura Academiei Române, Bucharest, 224 pp.

68. Petrescu A (1993) Contributions a la connaissance de la nourriture du Faucon Crecerelle (*Falco tinnunculus*) (Aves: Falconiformes) pendant la croissance des poussins. Travaux du Muséum National d'Histoire Naturelle Grigore Antipa 33: 441–451.

69. Petrescu A (1994) Contributions a la connaissance de la nourriture de la Chouette Cheveche, *Athene noctua* (Aves: Strigiformes). Travaux du Muséum National d'Histoire Naturelle Grigore Antipa 34: 391–400.

70. Petrescu A (1997) Restes de proies de la nourriture *d'Asio otus L.* (Aves: Strigiformes) pendant l'ete dans la Reserve Naturelle Agigea (Roumanie). Travaux du Muséum National d'Histoire Naturelle Grigore Antipa 37: 305–317.

71. Petrescu M, Cuzic V, Panait V (2012) Cercetări privind patrimoniul natural al comunei Beidaud. Delta Dunării. Studii și cercetări de științele naturii și muzeologie IV: 31–36.

72. Petrescu M, Cuzic V, Panait V (2012) Cercetări privind patrimoniul natural al comunei Casimcea. Delta Dunării. Studii și cercetări de științele naturii și muzeologie IV: 44–52.

73. Petrescu M, Cuzic V, Panait V (2012) Cercetări privind patrimoniul natural al comunei Ceatalchioi. Delta Dunării.Studii și cercetări de științele naturii și muzeologie IV: 55–62.

74. Petrescu M, Cuzic V, Panait V (2012) Cercetări privind patrimoniul natural al comunei Dorobanțu. Delta Dunării. Studii și cercetări de științele naturii și muzeologie IV: 8–89.

75. Petrescu M, Cuzic V, Panait V (2012) Cercetări privind patrimoniul natural al comunei Hamcearca. Delta Dunării. Studii și cercetări de științele naturii și muzeologie IV: 95–101.

76. Petrescu M, Cuzic V, Panait V (2012) Cercetări privind patrimoniul natural al comunei Izvoarele. Delta Dunării. Studii și cercetări de științele naturii și muzeologie IV:101–109.

77. Petrescu M, Cuzic V, Panait V (2012) Cercetări privind patrimoniul natural al comunei Nalbant. Delta Dunării. Studii și cercetări de științele naturii și muzeologie IV: 130–136.

78. Petrescu M, Cuzic V, Panait V (2012) Cercetări privind patrimoniul natural al comunei Pardina. Delta Dunării. Studii și cercetări de științele naturii și muzeologie IV: 142–147.

79. Petrescu M, Cuzic V, Panait V (2012) Cercetări privind patrimoniul natural al comunei Sfântu Gheorghe. Delta Dunării. Studii și cercetări de științele naturii și muzeologie IV: 162–171.

80. Petrescu M, Cuzic V, Panait V, Cuzic M (2012) Cercetări privind patrimoniul natural al comunei Baia. Delta Dunării. Studii și cercetări de științele naturii și muzeologie IV: 23–31.

81. Petrescu M, Cuzic V, Panait V, Cuzic M (2012) Cercetări privind patrimoniul natural al comunei Ceamurlia de Jos. Delta Dunării. Studii și cercetări de științele naturii și muzeologie IV:52–56.

82. Petrescu M, Cuzic V, Panait V, Cuzic M (2012) Cercetări privind patrimoniul natural al comunei Crișan. Delta Dunării. Studii și cercetări de științele naturii și muzeologie IV: 70–78.

83. Petrescu M, Cuzic V, Panait V, Cuzic M (2012) Cercetări privind patrimoniul natural al comunei Frecăței. Delta Dunării. Studii și cercetări de științele naturii și muzeologie IV: 89–95.

84. Petrescu M, Cuzic V, Panait V, Cuzic M (2012) Cercetări privind patrimoniul natural al comunei Jurilovca. Delta Dunării. Studii și cercetări de științele naturii și muzeologie IV: 109–116.

85. Petrescu M, Cuzic V, Panait V, Cuzic M (2012) Cercetări privind patrimoniul natural al comunei Murighiol. Delta Dunării. Studii și cercetări de științele naturii și muzeologie IV: 116–130.

86. Petrescu M, Cuzic V, Panait V, Cuzic M (2012) Cercetări privind patrimoniul natural al comunei Niculițel. Delta Dunării. Studii și cercetări de științele naturii și muzeologie IV: 136–142.

87. Petrescu M, Cuzic V, Panait V, Cuzic M (2012) Cercetări privind patrimoniul natural al comunei Sarichioi. Delta Dunării. Studii și cercetări de științele naturii și muzeologie VI: 153–162.

88. Petrescu M, Cuzic V, Panait V, Cuzic M, Dinu C (2012) Cercetări privind patrimoniul natural al comunei Somova. Delta Dunării. Studii și cercetări de științele naturii și muzeologie IV: 182–189.

89. Petrescu M, Cuzic V, Panait V, Cuzic M, Dinu C (2012) Cercetări privind patrimoniul natural al comunei Valea Nucarilor. Delta Dunării IV. Studii și cercetări de științele naturii și muzeologie IV: 203–212.

90. Petrescu M, Cuzic V, Panait V, Cuzic M, Radu A (2012) Cercetări privind patrimoniul natural al comunei Ciucurova. Delta Dunării. Studii și cercetări de științele naturii și muzeologie IV: 62–70.

91. Petrescu M, Cuzic V, Panait V, Cuzic M, Radu A (2012) Cercetări privind patrimoniul natural al comunei Slava Cercheză. Delta Dunării. Studii și cercetări de științele naturii și muzeologie IV: 171–182.

92. Petrescu M, Cuzic V, Panait V, Dinu C (2012) Cercetări privind patrimoniul natural al comunei Stejaru. Delta Dunării. Studii și cercetări de științele naturii și muzeologie IV: 189–194.

93. Petrescu M, Cuzic V, Panait V, Lefterache AM (2012) Cercetări privind patrimoniul natural al comunei Peceneaga. Delta Dunării. Studii și cercetări de științele naturii și muzeologie IV:147–153.

94. Petrescu M, Cuzic V, Panait V, Lefterache AM (2012) Cercetări privind patrimoniul natural al comunei Topolog. Delta Dunării. Studii și cercetări de științele naturii și muzeologie IV: 194–203.

95. Petrescu M, Cuzic V, Panait V (2014) Research concerning the natural heritage of Maliuc commune. Delta Dunării. Studii și cercetări de științele naturii și muzeologie V: 95–99.

96. Petrescu M, Cuzic V, Panait V (2014) Research concerning the natural heritage of Nufăru commune. Delta Dunării. Studii și cercetări de științele naturii și muzeologie V: 114–121.

97. Petrescu M, Cuzic V, Panait V (2014) Research concerning the natural heritage of Tulcea town. Delta Dunării. Studii și cercetări de științele naturii și muzeologie V: 138–145.

98. Petrescu M, Cuzic V., Panait, V (2014) Research concerning the natural heritage of Văcăreni commune. Delta Dunării. Studii și cercetări de științele naturii și muzeologie V: 150–154.

99. Petrescu M, Cuzic V, Panait V, Cuzic M (2014) Research concerning the natural heritage of Beștepe commune. Delta Dunării. Studii și cercetări de științele naturii și muzeologie V: 15–23.

100. Petrescu M, Cuzic V, Panait V, Cuzic M (2014) Research concerning the natural heritage of Mahmudia commune. Delta Dunării. Studii și cercetări de științele naturii și muzeologie V: 87–95.

101. Popescu A (1967) Contribuţii la studiul sistematicii şi dinamicii helmintofaunei populaţiilor de rozătoare din Dobrogea de Nord. Studii şi Cercetări de Biologie, Seria Zoologie 6: 501–508.

102. Popescu A (1968) Observaţii asupra rozătoarelor din nord-vestul Dobrogii. Studii şi Cercetări de Biologie, Seria Zoologie 2: 153–163.

103. Popescu A (1969) Contribuţii la studiul sistematic, biologic şi ecologic al rozătoarelor (Rodentia) din Dobrogea de Nord. Teză doctorat, București, România: Universitatea Bucureşti.

104. Popescu A (1972) Nourriture du suslik d'Europe (*Citellus citellus* L.) dans les conditions de la steppe et sylvosteppe en Dobroudja. Analele Universităţii Bucureşti, Seria Biologie animală 21: 89–94.

105. Popescu A, Barbu P (1979) Date privind răspândirea şi frecvenţa soricidelor (Soricidae - Insectivora) în România. Ocrotirea Naturii şi a Mediului Înconjurător 2: 163–168.

106. Popescu A, Sin Gh (1966) Despre cuibăritul și hrana bufniței (*Bubo bubo bubo L.)* în Dobrogea de Nord. Ocrotirea Naturii 10 (2): 217–222.

107. Popescu A, Sin Gh (1967) Le terrier et la nourriture du blaireau (*Meles meles* L.) dans les conditions de la steppe de Dobroudja. Travaux du Muséum d'Histoire Naturelle Grigore Antipa VIII, 2: 1003-1012.

108. Popescu A, Murariu D (2001) Fauna României. Mammalia. Rodentia. Editura Academiei Române, Bucharest, 214 pp.

109. Popovici Z (1943) Das Verhalten Der Dornhaie Zu Den Delphinen Im Schwarzen Meere. Analele Institutului de Cercetări Piscicole al României Vol. II.

110. Radu G, Anton E, Nenciu M, Spînu AD (2013) Distribution and abundance of Cetaceans in the Romanian Marine Area. Cercetări Marine 43: 320–341.

111. Răduleţ N (2005) Contributions to the knowledge of the mammal fauna from Dobrogea (Roumania). Travaux du Muséum National d'Histoire Naturelle Grigore Antipa XLVIII: 417-425.

112. Răduleţ N, Stănescu M (1996) Contributions à la connaissance des mammifères du sud de la Dobrogea (Roumanie). Travaux du Muséum National d'Histoire Naturelle Grigore Antipa 36: 373–384.

113. Rudescu L, (1955) Bizamul în Delta Dunării. Vânătorul şi Pescarul Sportiv 5: 17.

114. Sandor DA, Bugariu S (2008) Food habits of the Eurasian Eagle Owl (*Bubo bubo*) in Cheile Dobrogei Gorge. Scientific Annals of the Danube Delta Institute 14: 69–74.

115. Sandor DA, Kiss JB (2004) The diet of wintering Long-eared Owls (*Asio otus)* in Tulcea, Romania. Analele Științifice ale Institutului Delta Dunării 10:49–54.

116. Schnapp B (1971) New data concerning the Valul lui Traian micromammal and bird fauna in the winters of 1957/1958 – 1961/1962 according to *Asio otus* (L.) pellets. Travaux du Muséum d'Histoire Naturelle Grigore Antipa 11: 493-510.

117. Simionescu V (1971) Studii privind sistematica și variabilitatea geografică a genului *Micromys* Dehne, 1841 (Ord. Rodentia). Studii şi Comununicări, Muzeul de Ştiinţele naturii Bacău 365-392.

118. Suciu M (1971) Date ecologice asupra sifonapterelor parazite pe mamifere mici (Insectivora, Rodentia) din Dobrogea de Nord şi Delta Dunării. Studii şi Cercetări de Biologie, Seria Zoologie 2: 173–184.

119. Timu G (1991) Şacalii în Dobrogea, Vânătorul şi Pescarul Român 6: 11.

120. Vasiliu GD (1964) Der Marderhund (*Nyctereutes procyonoides* Gray) in Rumänien. Zeitschrift für Jagdwissenschaft. 10 (2): 51–54.

121. Vasiliu GD, Almășan H (1969) Contribuţii la cunoaşterea taxonometriei unor mamifere (Carnivora) din România. Studii şi Comunicări, Muzeul Judeţean Bacău 283-296.

122. Weber P (2000) Aves Histriae. Avifauna zonei Histria. Rezervația Biosferei Deltei Dunării. Editura AVES.
