# Supplementary material for: Conservation priorities for terrestrial mammals in Dobrogea Region, Romania
Source: Zookeys. 2018 Oct 23;(792):133–58. doi: 10.3897/zookeys.792.25314 (PMC6215976; doi:10.3897/zookeys.792.25314)
Supplement: Supplementary material 2 — Occurrences maps for 59 mammal species [file zookeys-792-133-s002.pdf]

Iulia V. Miu, Gabriel B. Chişamera, Viorel D. Popescu, Ruben Iosif, Andreea Nita, Steluta Manolache, Viorel D. Gavril, Ioana Cobzaru, Laurentiu Rozylowicz (2018) Conservation priorities for terrestrial mammals in Dobrogea Region, Romania. Zookeys

Appendix 2

The mammal species old (before 1990) and new (after 1990) occurrence records at a 5 × 5 km grid resolution within Dobrogea, Romania

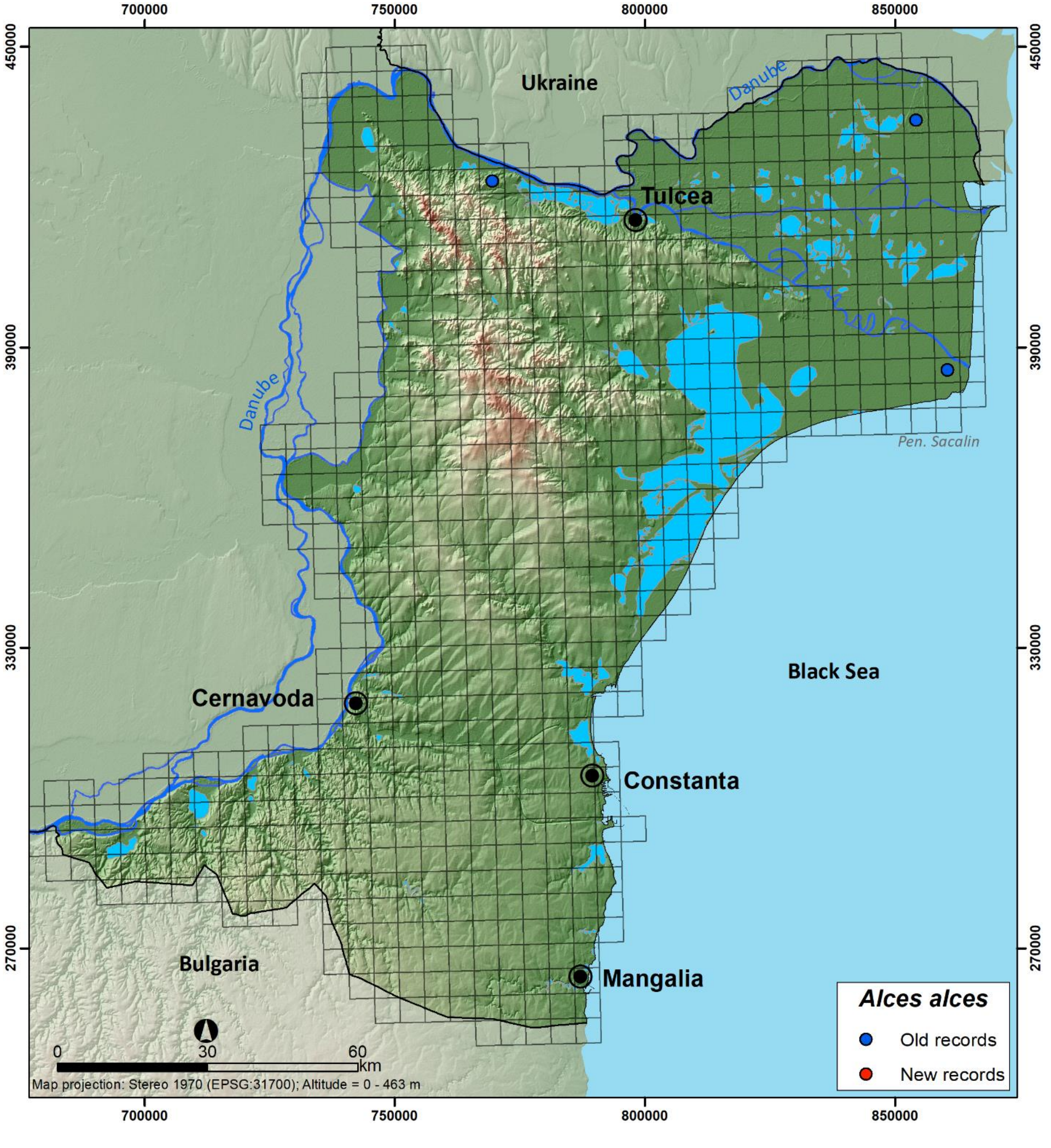

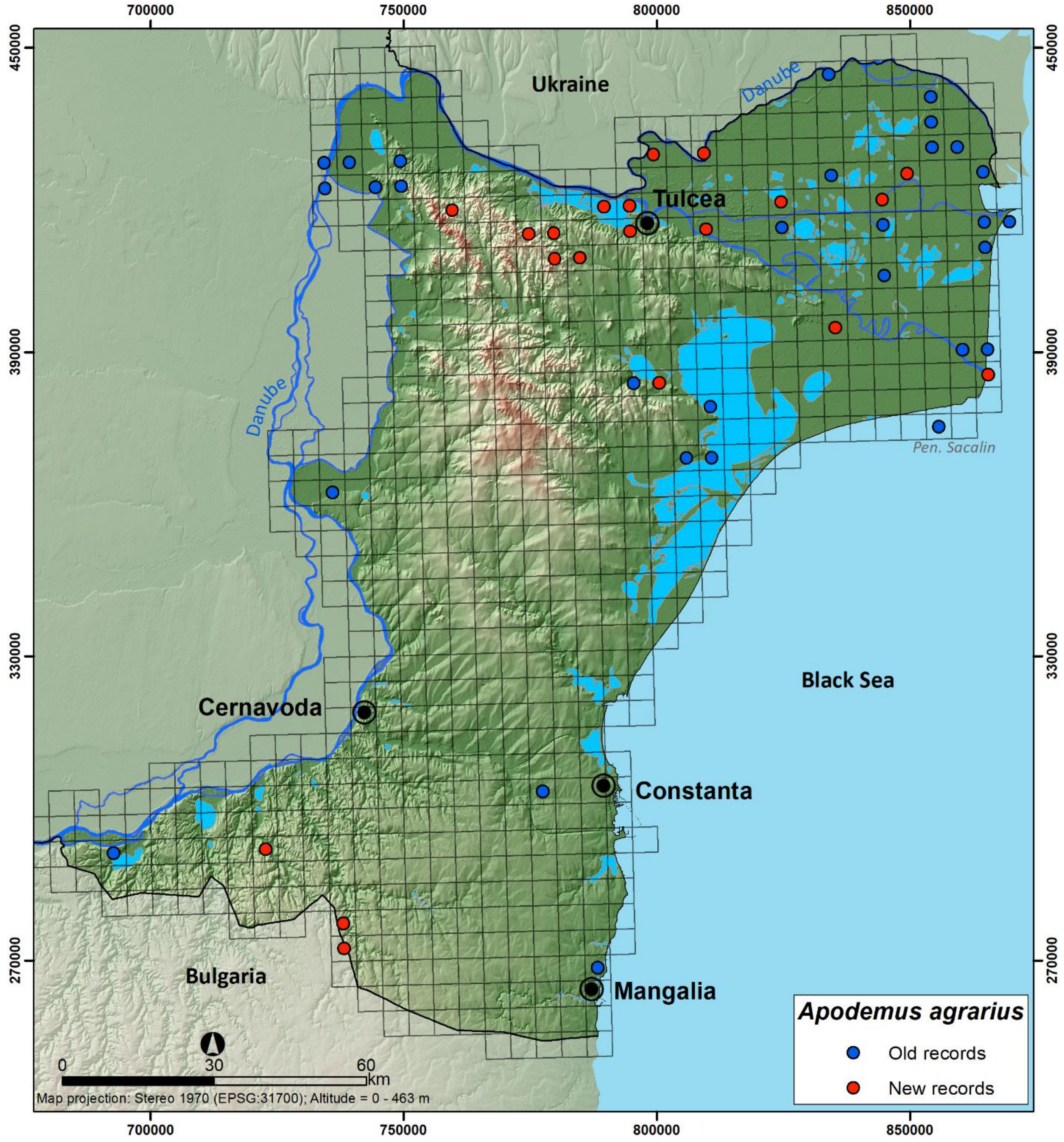

700000
750000
800000
850000
450000
390000
330000
270000
Ukraine
Danube
Tulcea
Pen. Sacalin
Black Sea
Cernavoda
Constanta
Mangalia
Bulgaria
0
30
60
km
Map projection: Stereo 1970 (EPSG:31700); Altitude = 0 - 463 m
Apodemus agrarius
Old records
New records

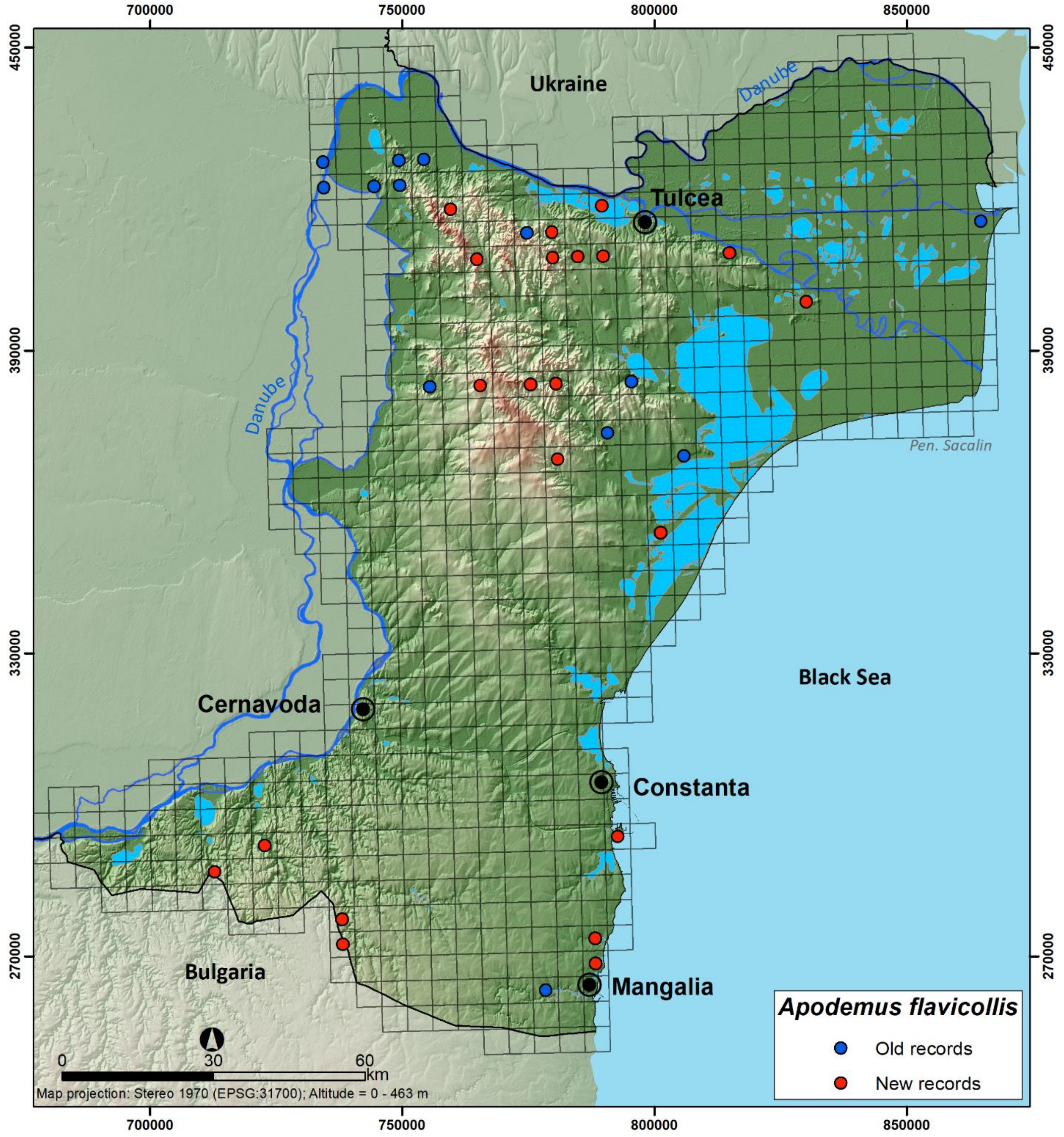
700000
750000
800000
850000
450000
390000
330000
270000
Ukraine
Danube
Tulcea
Pen. Sacalin
Black Sea
Cernavoda
Constanta
Mangalia
Bulgaria
0
30
60
km
Map projection: Stereo 1970 (EPSG:31700); Altitude = 0 - 463 m
Apodemus flavicollis
Old records
New records

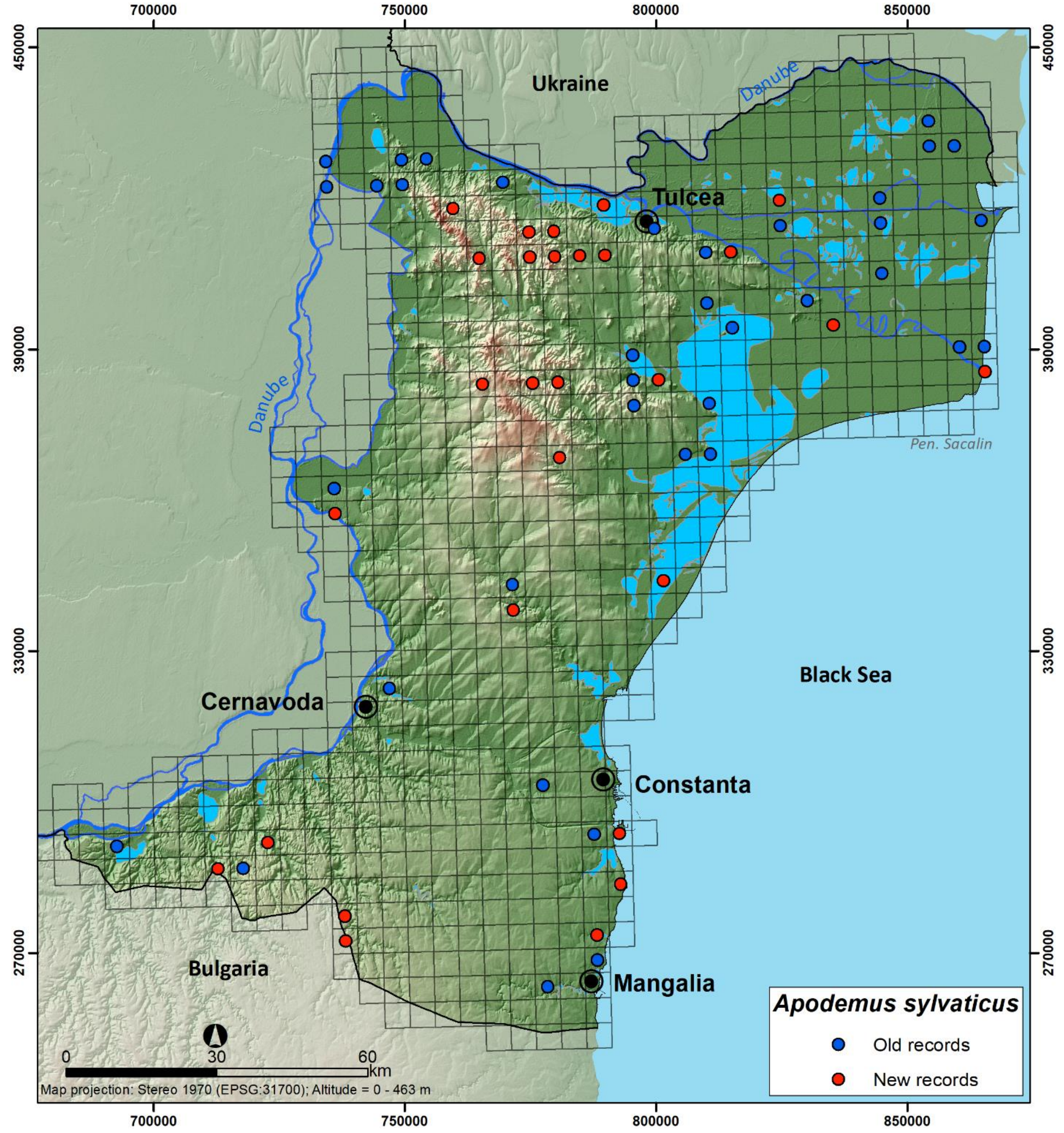

700000
750000
800000
850000
450000
390000
330000
270000
Ukraine
Danube
Tulcea
Pen. Sacalin
Black Sea
Cernavoda
Constanta
Mangalia
Bulgaria
0
30
60
km
Map projection: Stereo 1970 (EPSG:31700); Altitude = 0 - 463 m
Apodemus sylvaticus
Old records
New records

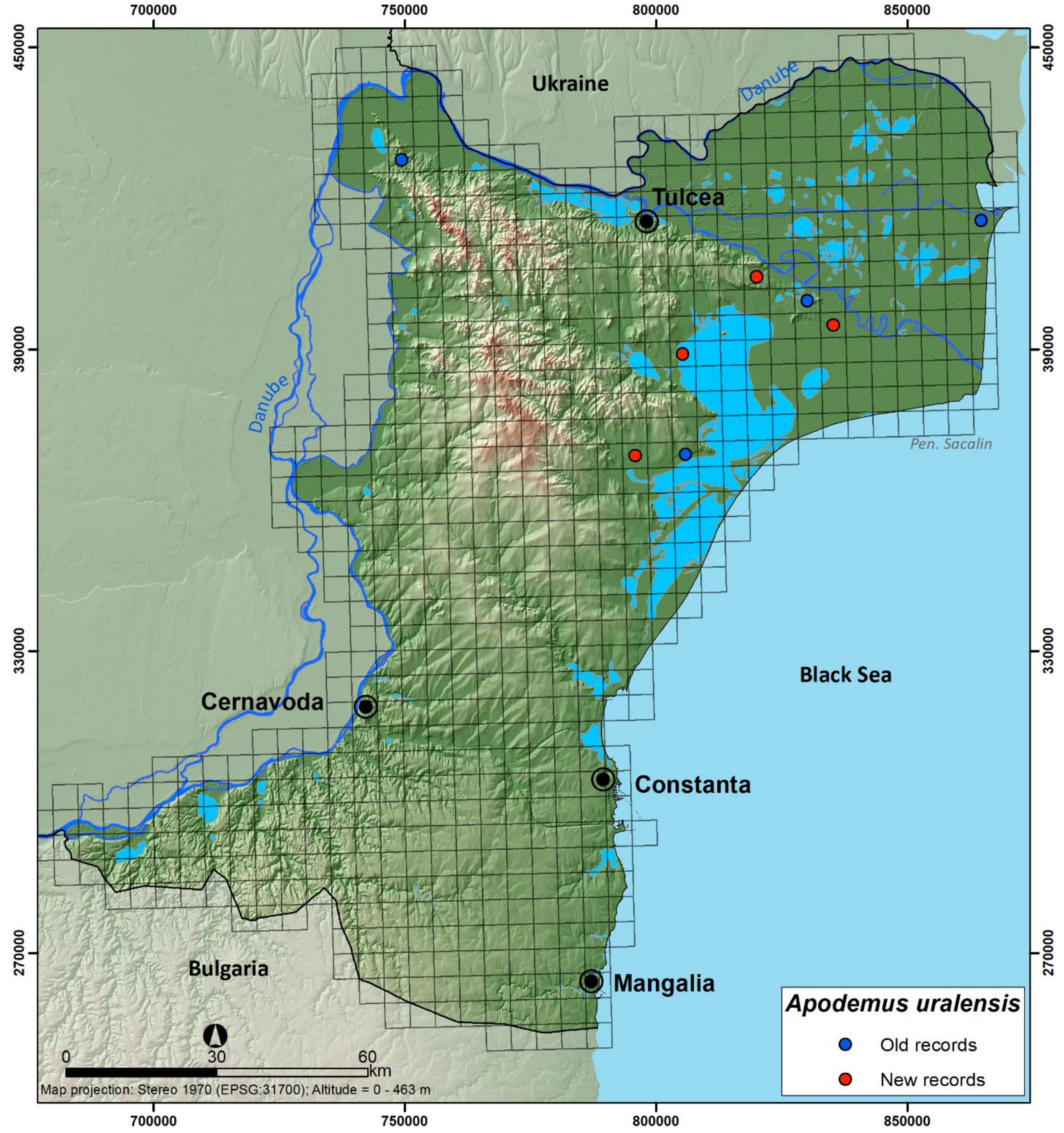
700000
750000
800000
850000
450000
390000
330000
270000
Ukraine
Danube
Tulcea
Danube
Pen. Sacalin
Black Sea
Cernavoda
Constanta
Bulgaria
Mangalia
Apodemus uralensis
Old records
New records
0
30
60
km
Map projection: Stereo 1970 (EPSG:31700); Altitude = 0 - 463 m

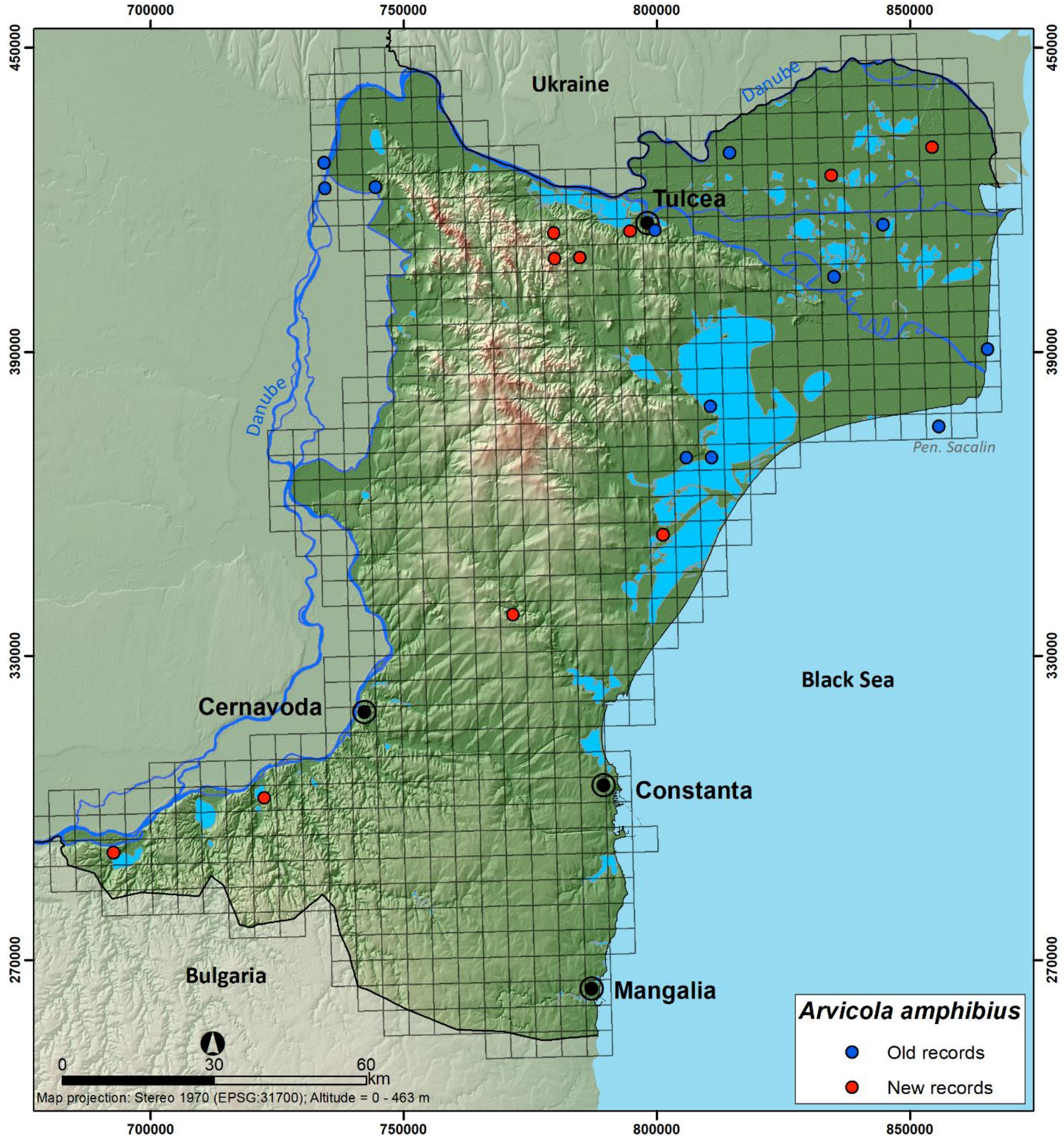
700000
750000
800000
850000
450000
390000
330000
270000
Ukraine
Danube
Tulcea
Danube
Pen. Sacalin
Cernavoda
Black Sea
Constanta
Bulgaria
Mangalia
Arvicola amphibius
Old records
New records
0
30
60
km
Map projection: Stereo 1970 (EPSG:31700); Altitude = 0 - 463 m

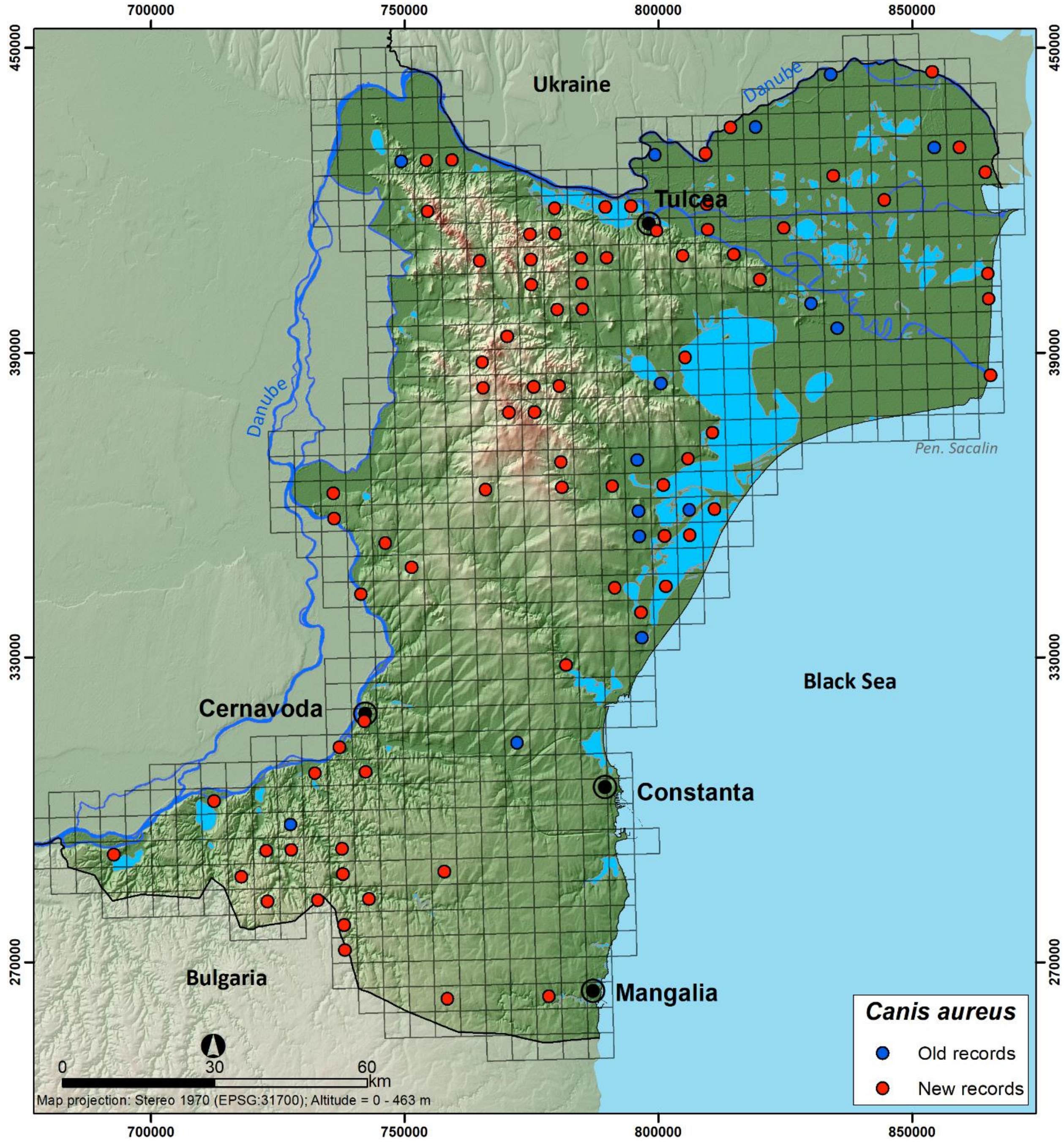
700000
750000
800000
850000
450000
390000
330000
270000
Ukraine
Danube
Tulcea
Pen. Sacalin
Black Sea
Cernavoda
Constanta
Bulgaria
Mangalia
Canis aureus
Old records
New records
0
30
60
km
Map projection: Stereo 1970 (EPSG:31700); Altitude = 0 - 463 m

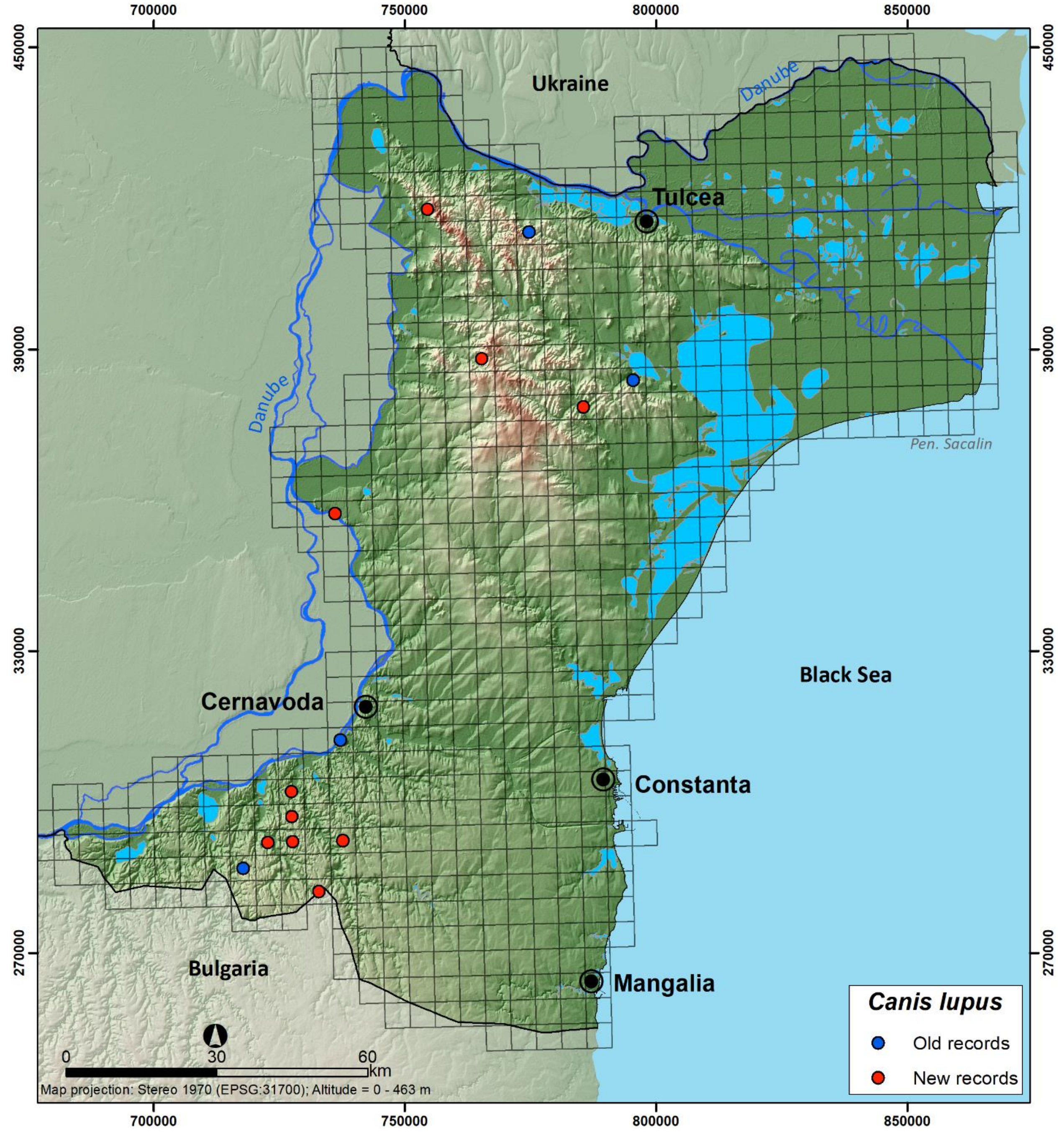

700000
750000
800000
850000
450000
390000
330000
270000
Ukraine
Danube
Tulcea
Pen. Sacalin
Cernavoda
Constanta
Black Sea
Bulgaria
Mangalia
0
30
60
km
Map projection: Stereo 1970 (EPSG:31700); Altitude = 0 - 463 m
Canis lupus
Old records
New records

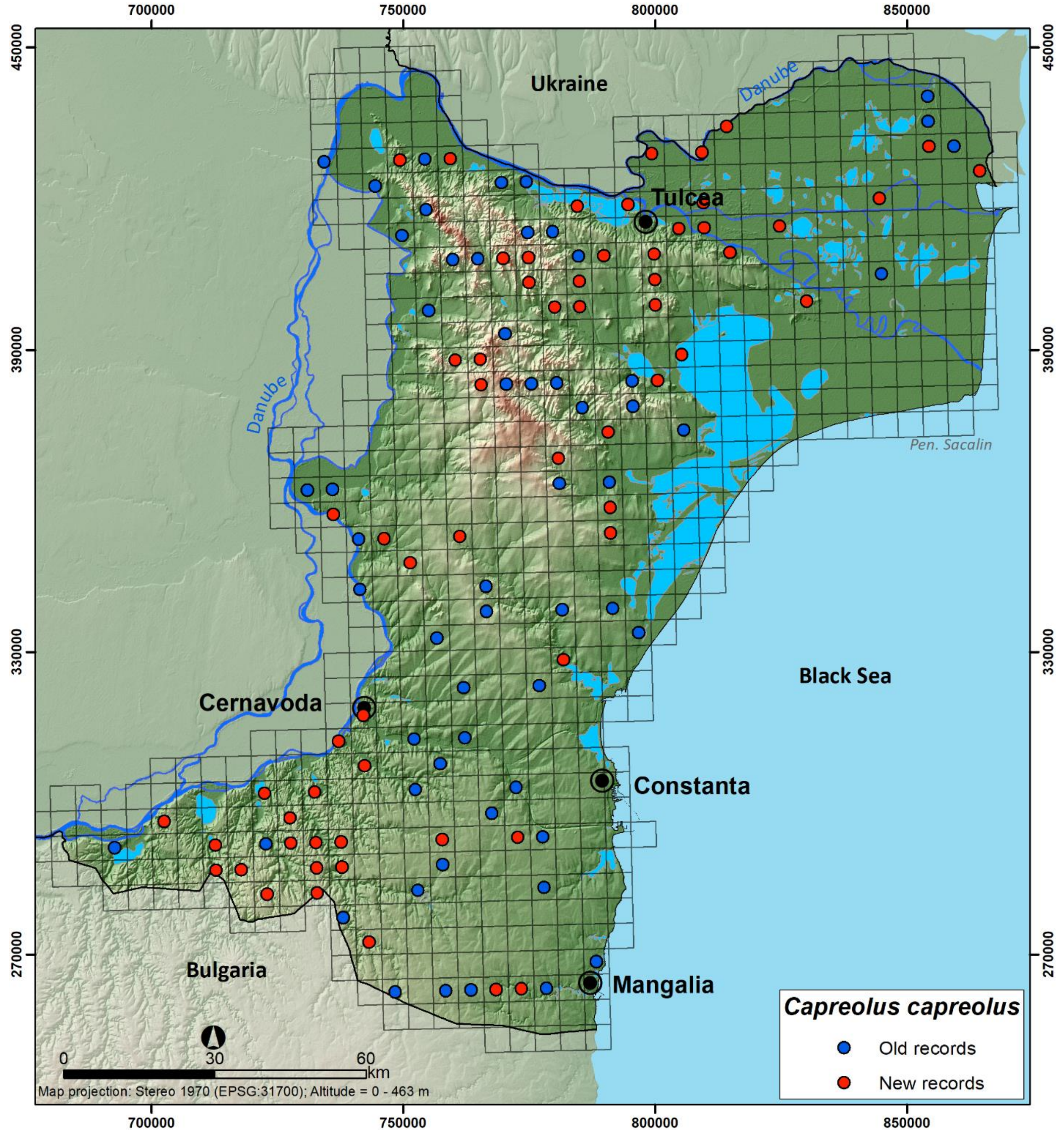

700000
750000
800000
850000
450000
390000
330000
270000
Ukraine
Danube
Tulcea
Danube
Pen. Sacalin
Black Sea
Cernavoda
Constanta
Bulgaria
Mangalia
Capreolus capreolus
Old records
New records
0
30
60
km
Map projection: Stereo 1970 (EPSG:31700); Altitude = 0 - 463 m

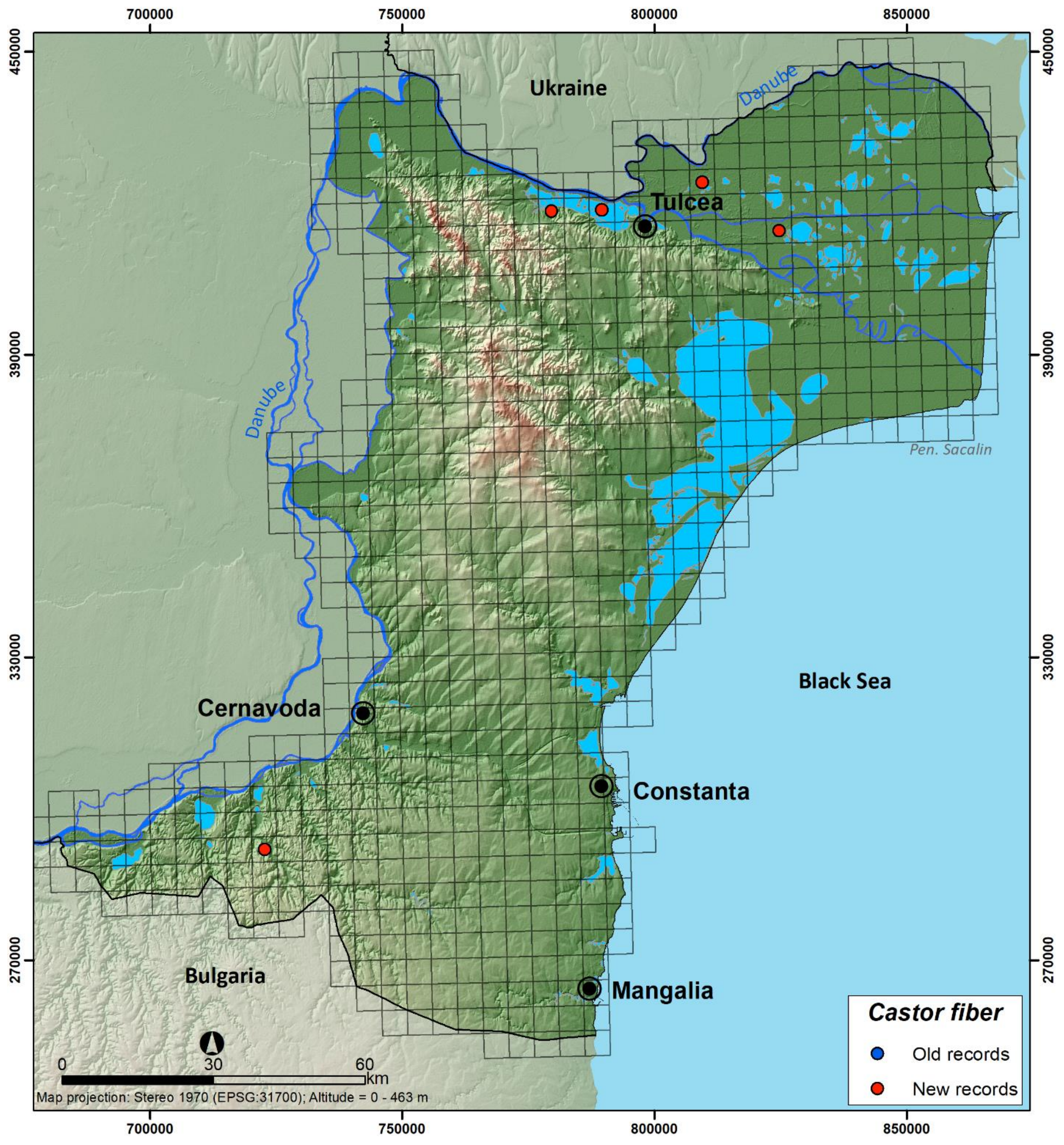

700000
750000
800000
850000
450000
390000
330000
270000
Ukraine
Danube
Tulcea
Pen. Sacalin
Cernavoda
Constanta
Black Sea
Bulgaria
Mangalia
Castor fiber
Old records
New records
0
30
60
km
Map projection: Stereo 1970 (EPSG:31700); Altitude = 0 - 463 m

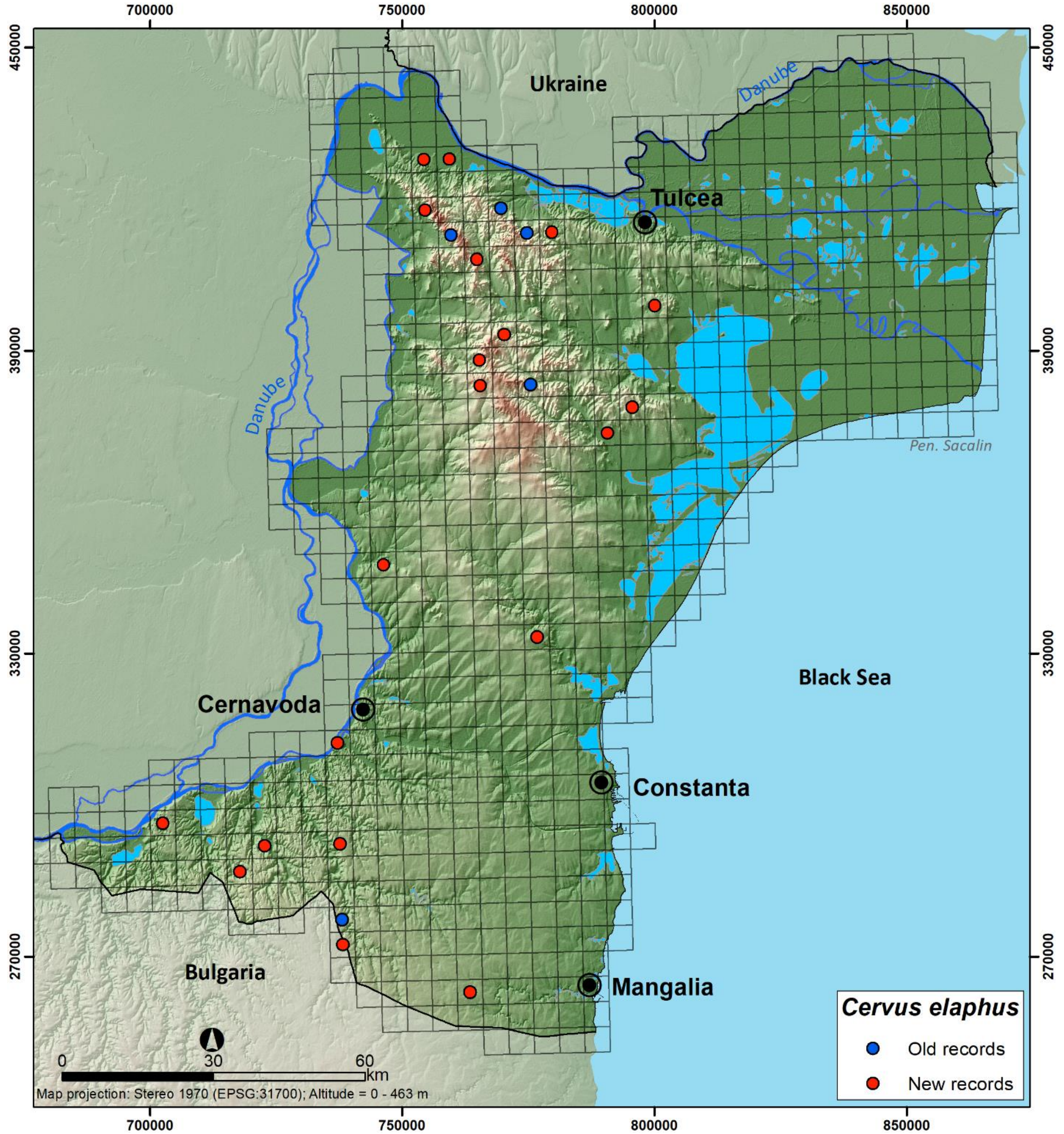
700000
750000
800000
850000
450000
390000
330000
270000
Ukraine
Danube
Tulcea
Pen. Sacalin
Cernavoda
Black Sea
Constanta
Bulgaria
Mangalia
Cervus elaphus
Old records
New records
0
30
60
km
Map projection: Stereo 1970 (EPSG:31700); Altitude = 0 - 463 m

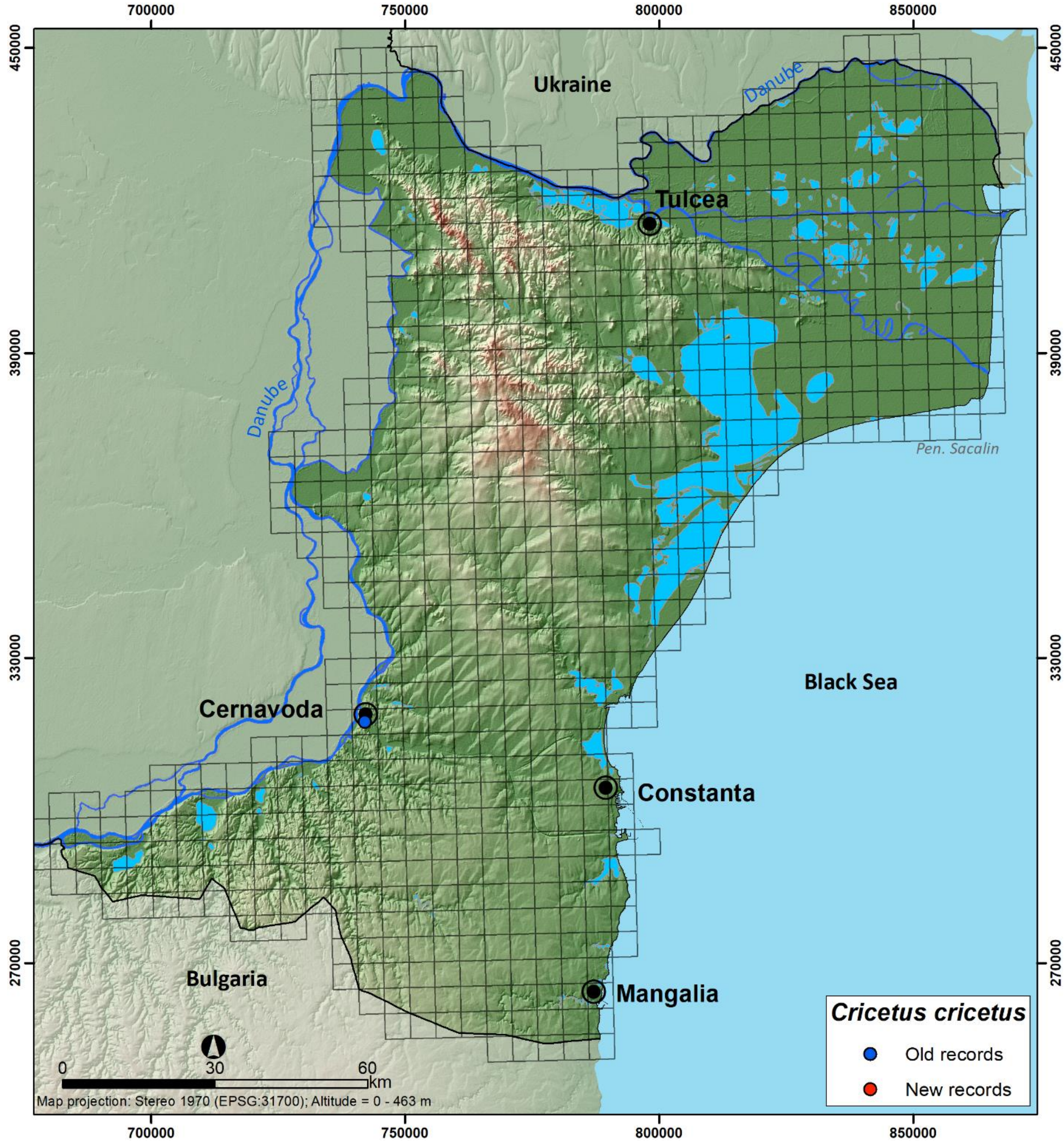

700000
750000
800000
850000
450000
390000
330000
270000
Ukraine
Danube
Tulcea
Danube
Pen. Sacalin
Black Sea
Cernavoda
Constanta
Bulgaria
Mangalia
Cricetus cricetus
Old records
New records
0
30
60
km
Map projection: Stereo 1970 (EPSG:31700); Altitude = 0 - 463 m

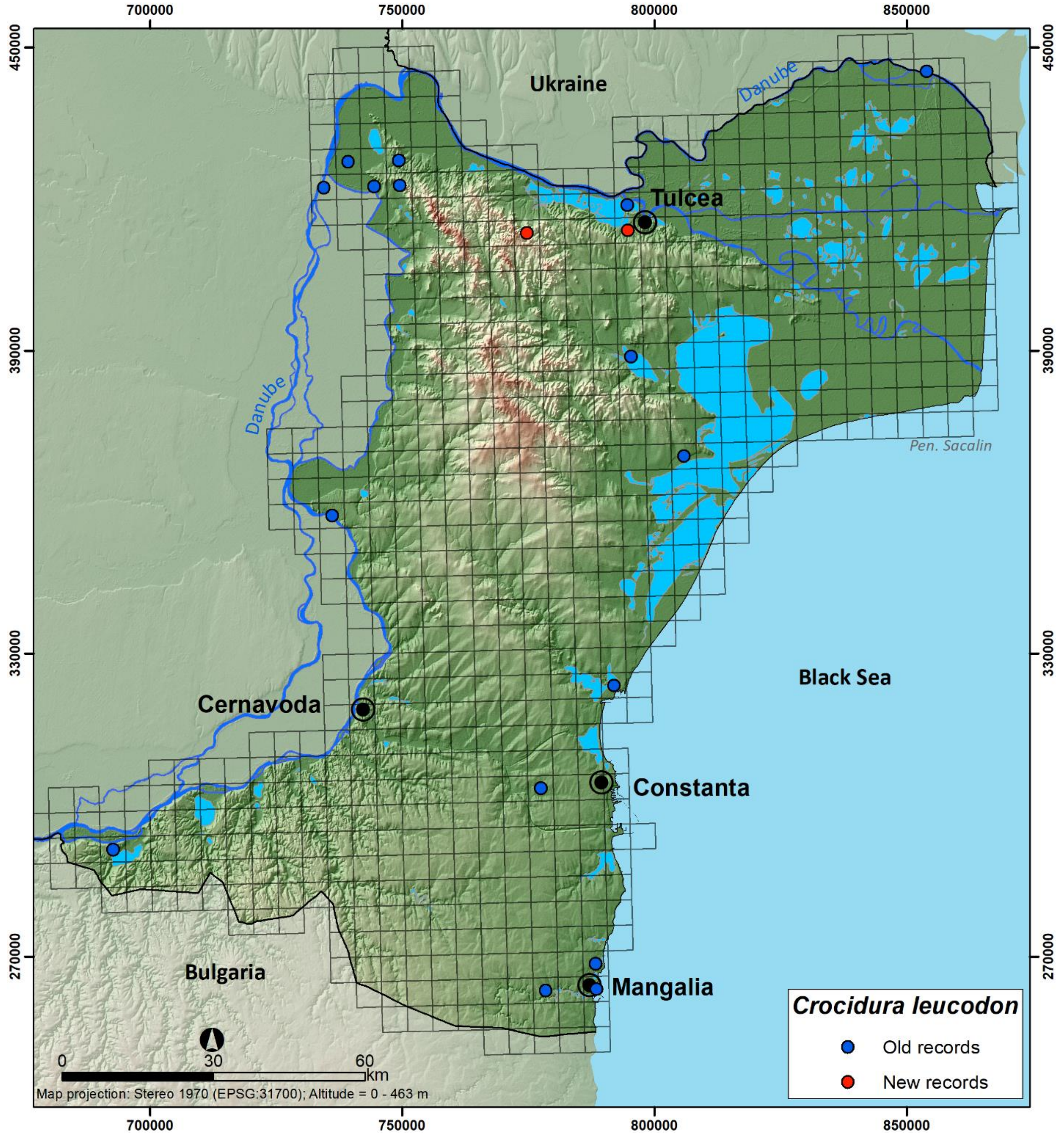
700000
750000
800000
850000
450000
390000
330000
270000
Ukraine
Danube
Tulcea
Pen. Sacalin
Black Sea
Cernavoda
Constanta
Bulgaria
Mangalia
Crocidura leucodon
Old records
New records
0
30
60
km
Map projection: Stereo 1970 (EPSG:31700); Altitude = 0 - 463 m

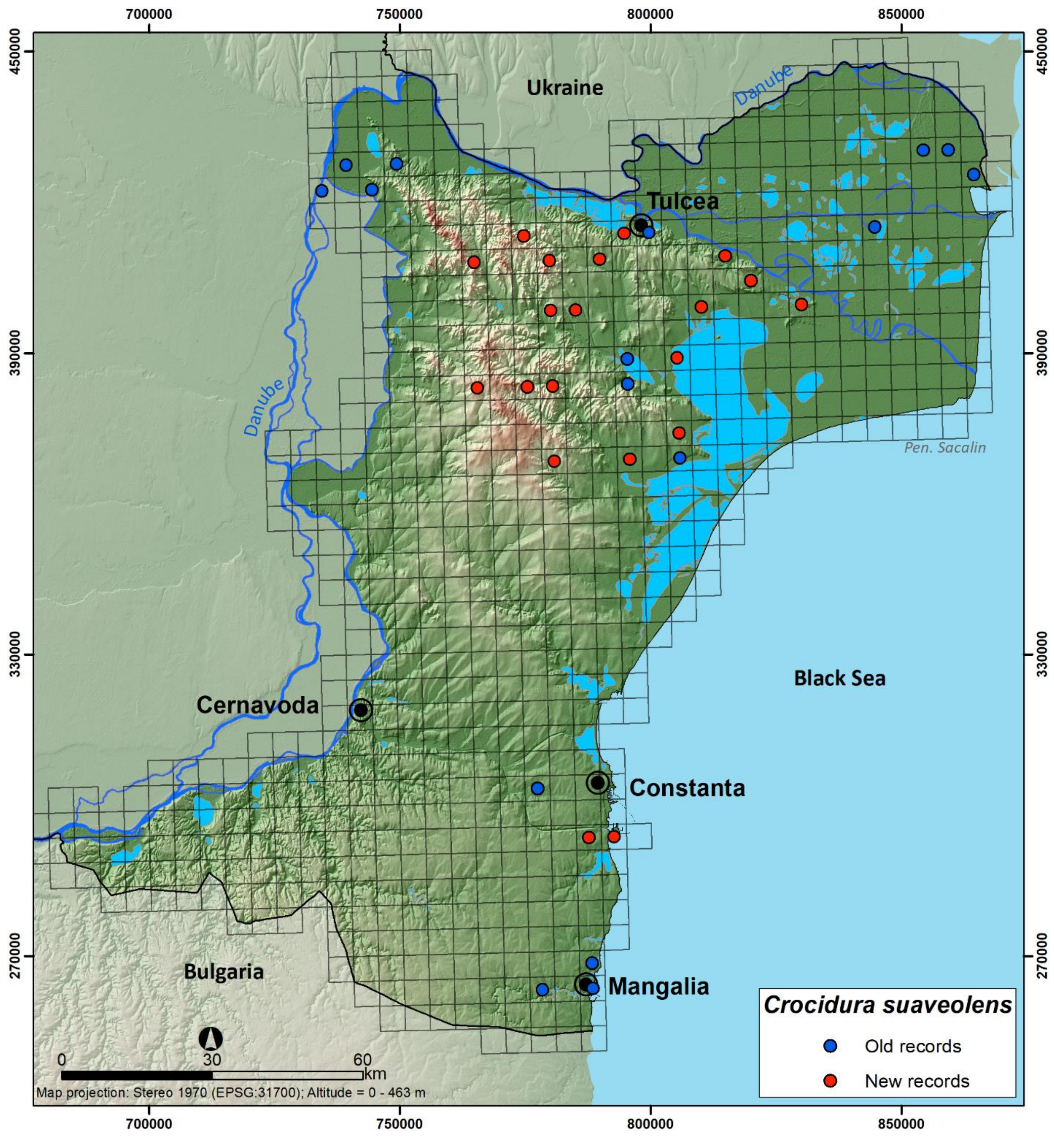
700000
750000
800000
850000
450000
390000
330000
270000
Ukraine
Danube
Tulcea
Pen. Sacalin
Cernavoda
Constanta
Black Sea
Mangalia
Bulgaria
Crocidura suaveolens
Old records
New records
0
30
60
km
Map projection: Stereo 1970 (EPSG:31700); Altitude = 0 - 463 m

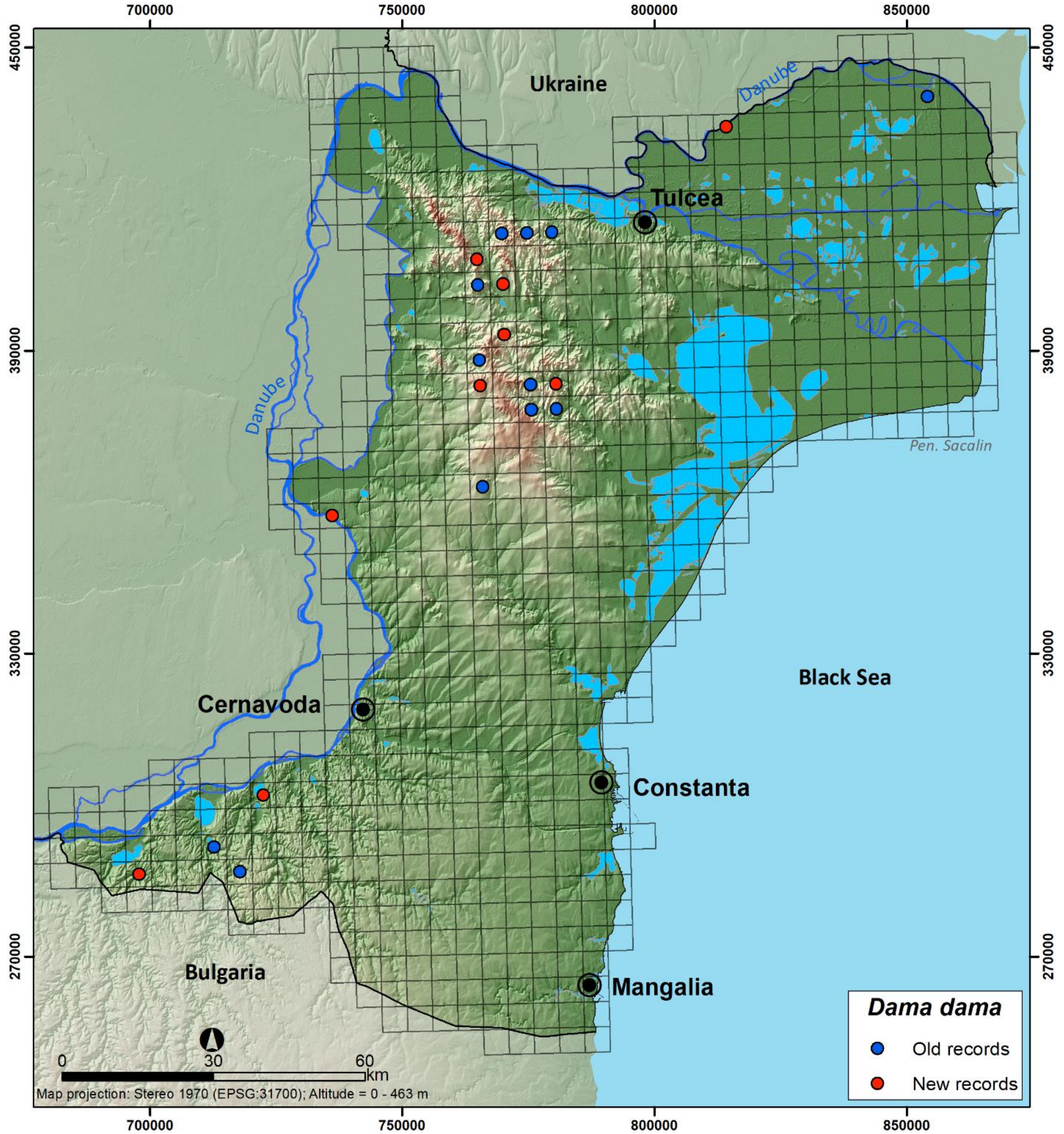
700000
750000
800000
850000
450000
390000
330000
270000
Ukraine
Danube
Tulcea
Pen. Sacalin
Black Sea
Cernavoda
Constanta
Bulgaria
Mangalia
0
30
60
km
Map projection: Stereo 1970 (EPSG:31700); Altitude = 0 - 463 m
Dama dama
Old records
New records

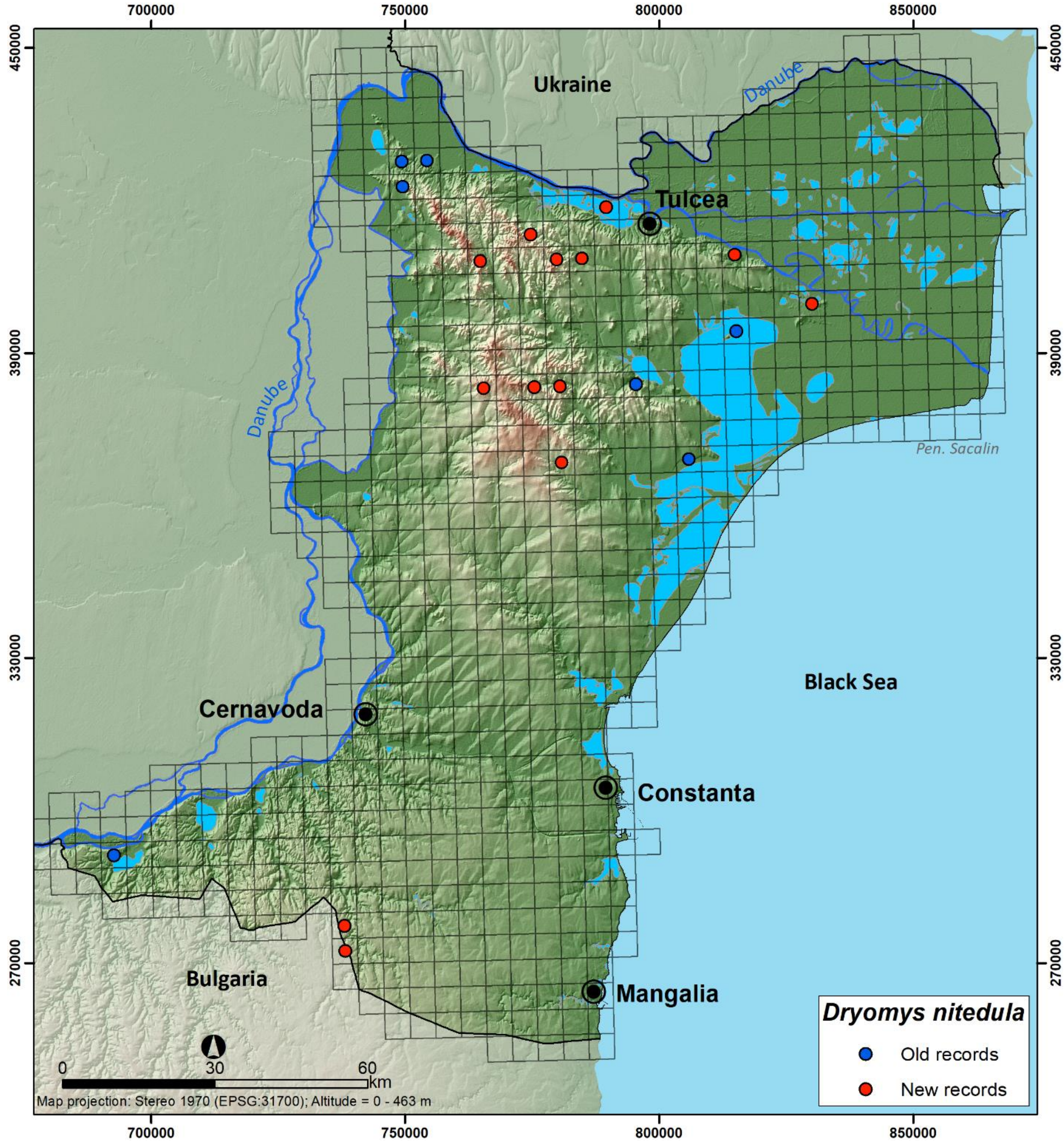

700000
750000
800000
850000
450000
390000
330000
270000
Ukraine
Danube
Tulcea
Pen. Sacalin
Black Sea
Cernavoda
Constanta
Bulgaria
Mangalia
Dryomys nitedula
Old records
New records
0
30
60
km
Map projection: Stereo 1970 (EPSG:31700); Altitude = 0 - 463 m

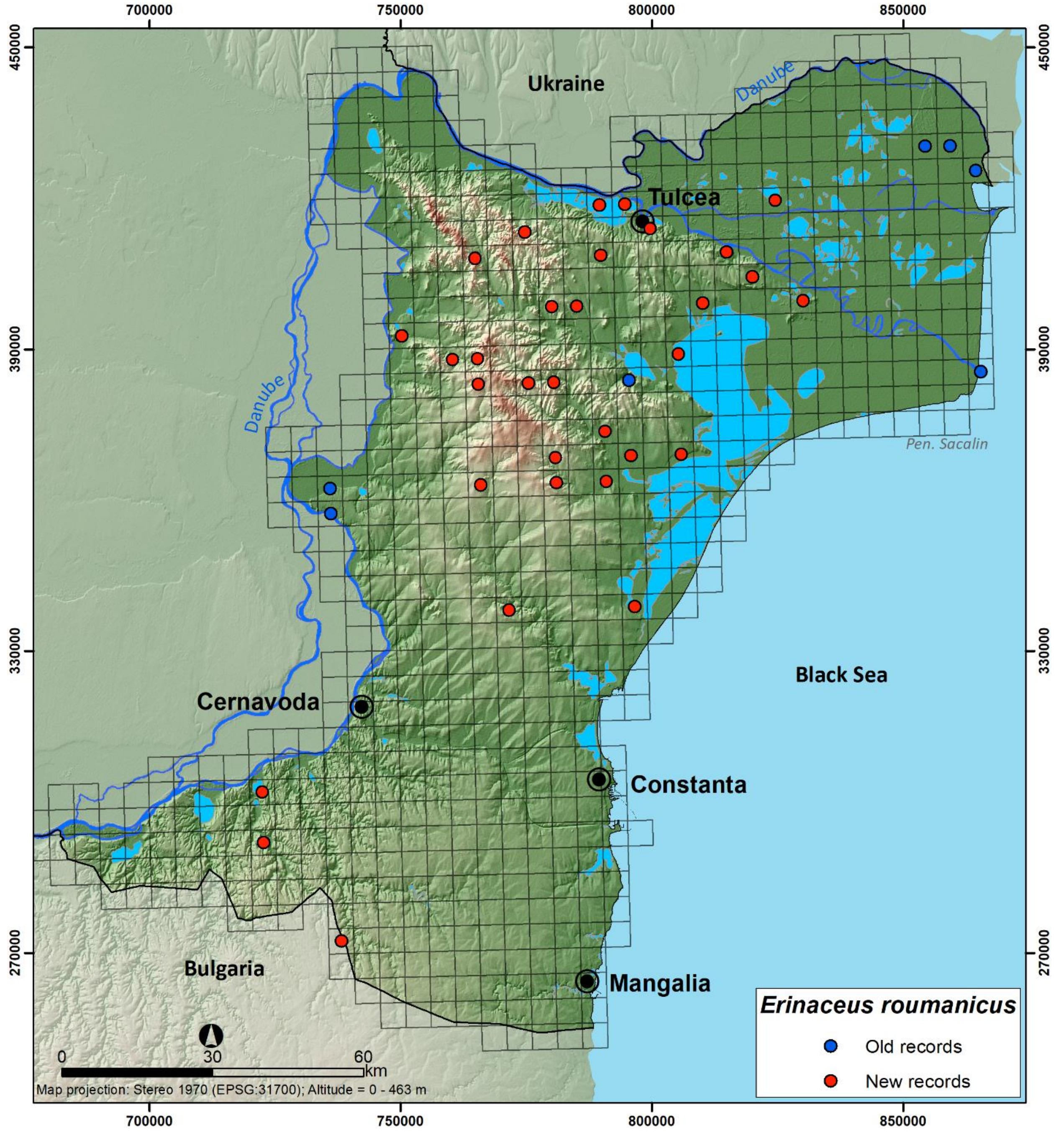
700000
750000
800000
850000
450000
390000
330000
270000
Ukraine
Danube
Tulcea
Danube
Pen. Sacalin
Black Sea
Cernavoda
Constanta
Bulgaria
Mangalia
0
30
60
km
Map projection: Stereo 1970 (EPSG:31700); Altitude = 0 - 463 m
Erinaceus roumanicus
Old records
New records

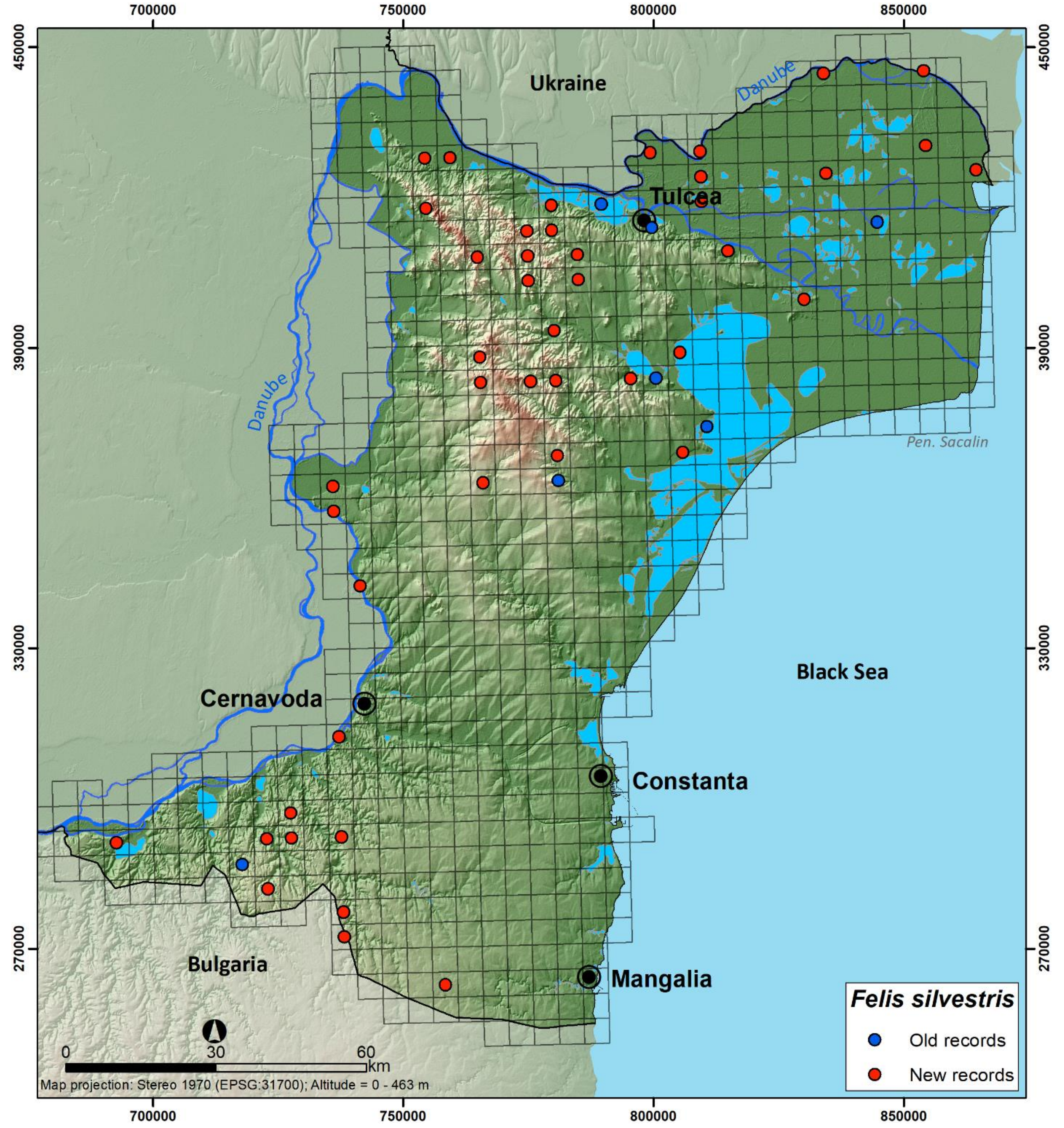
700000
750000
800000
850000
450000
390000
330000
270000
Ukraine
Danube
Tulcea
Pen. Sacalin
Black Sea
Cernavoda
Constanta
Mangalia
Bulgaria
0
30
60
km
Map projection: Stereo 1970 (EPSG:31700); Altitude = 0 - 463 m
Felis silvestris
Old records
New records

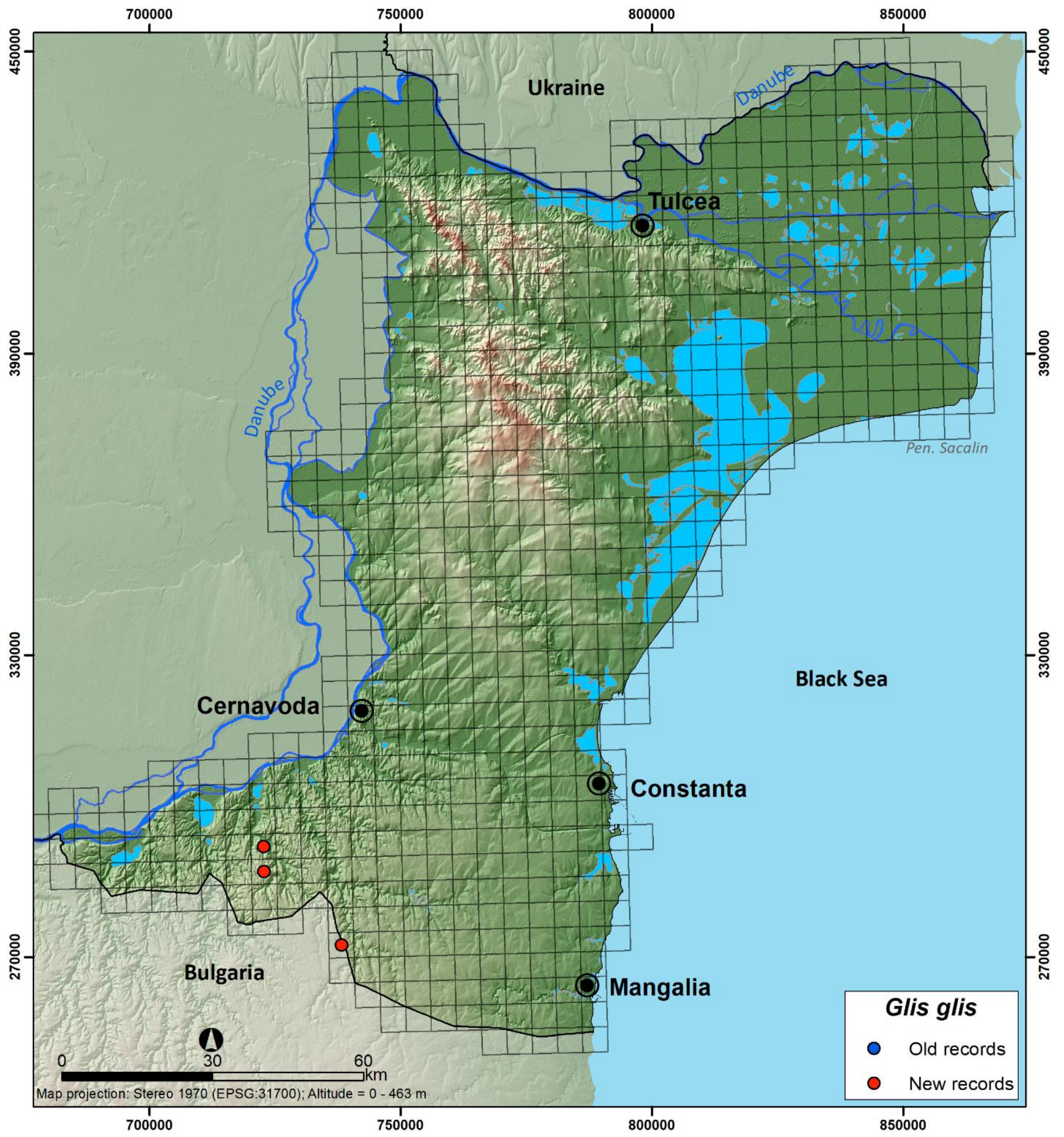

700000
750000
800000
850000
450000
390000
330000
270000
Ukraine
Danube
Tulcea
Pen. Sacalin
Cernavoda
Constanta
Black Sea
Bulgaria
Mangalia
Glis glis
Old records
New records
0
30
60
km
Map projection: Stereo 1970 (EPSG:31700); Altitude = 0 - 463 m

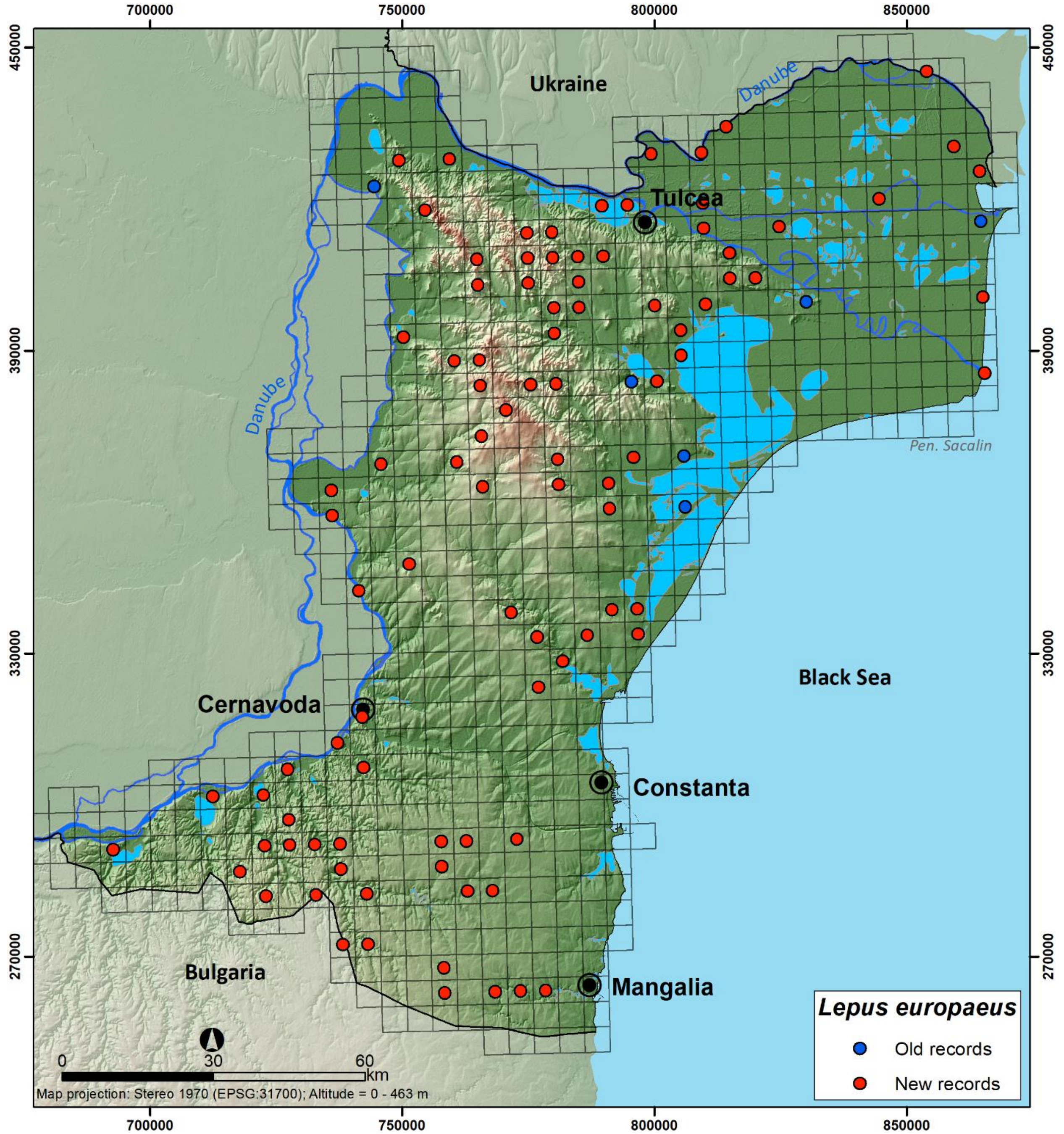

700000
750000
800000
850000
450000
390000
330000
270000
Ukraine
Danube
Tulcea
Pen. Sacalin
Black Sea
Cernavoda
Constanta
Mangalia
Bulgaria
Lepus europaeus
Old records
New records
0
30
60
km
Map projection: Stereo 1970 (EPSG:31700); Altitude = 0 - 463 m

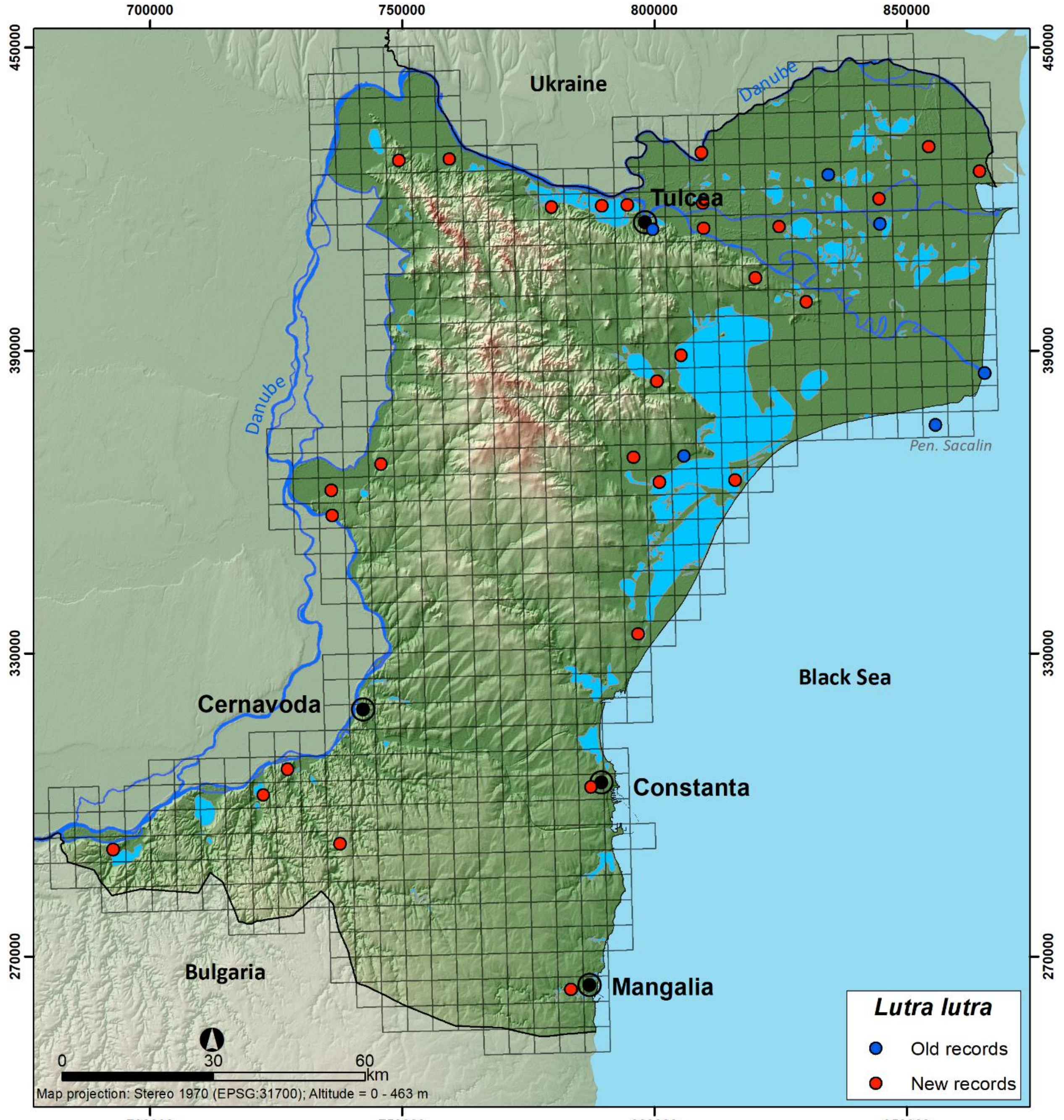
700000
750000
800000
850000
450000
390000
330000
270000
Ukraine
Danube
Tulcea
Danube
Pen. Sacalin
Black Sea
Cernavoda
Constanta
Bulgaria
Mangalia
Lutra lutra
Old records
New records
0
30
60
km
Map projection: Stereo 1970 (EPSG:31700); Altitude = 0 - 463 m

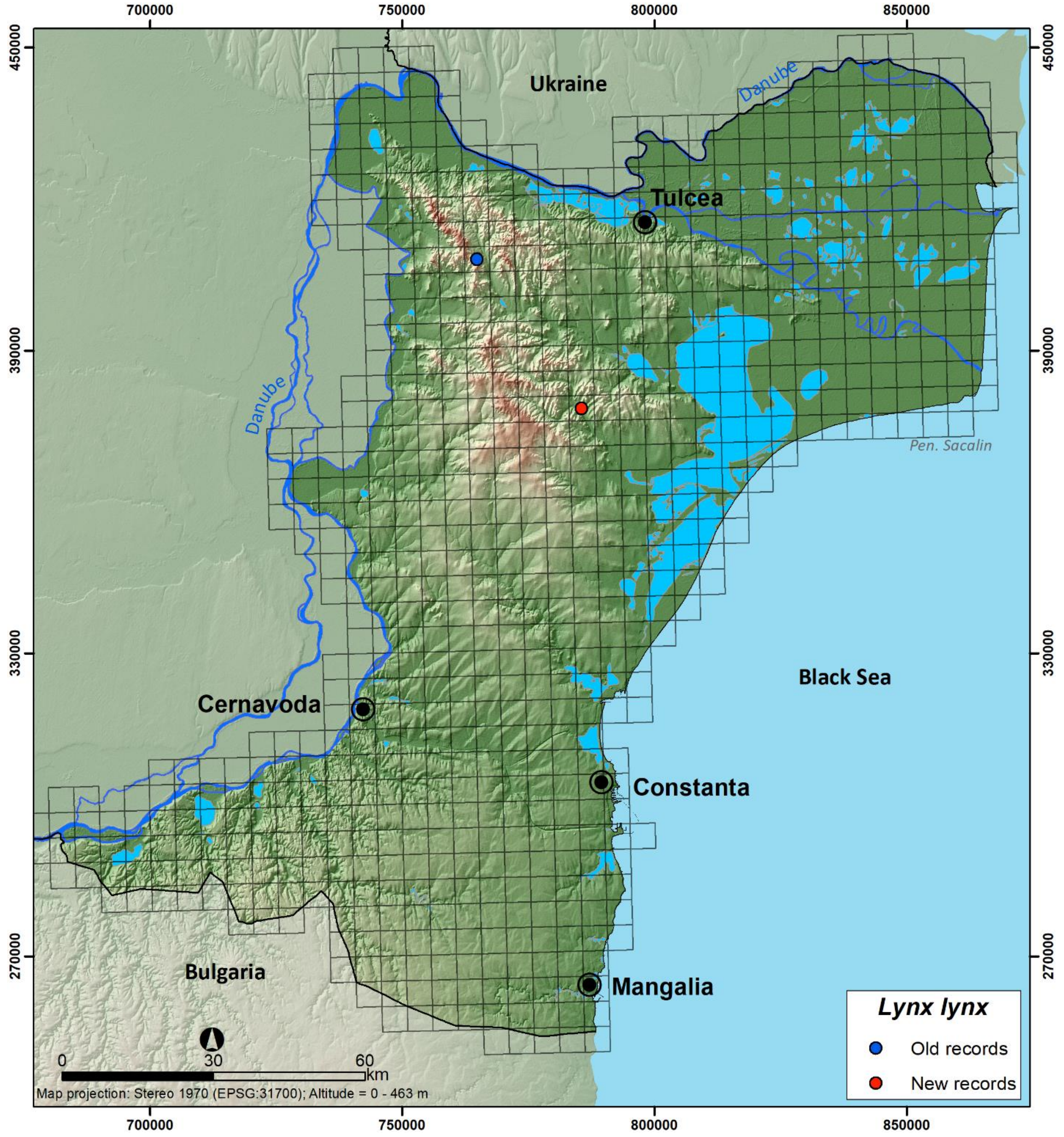
700000
750000
800000
850000
450000
390000
330000
270000
Ukraine
Danube
Tulcea
Danube
Pen. Sacalin
Black Sea
Cernavoda
Constanta
Bulgaria
Mangalia
0
30
60
km
Map projection: Stereo 1970 (EPSG:31700); Altitude = 0 - 463 m
Lynx lynx
Old records
New records

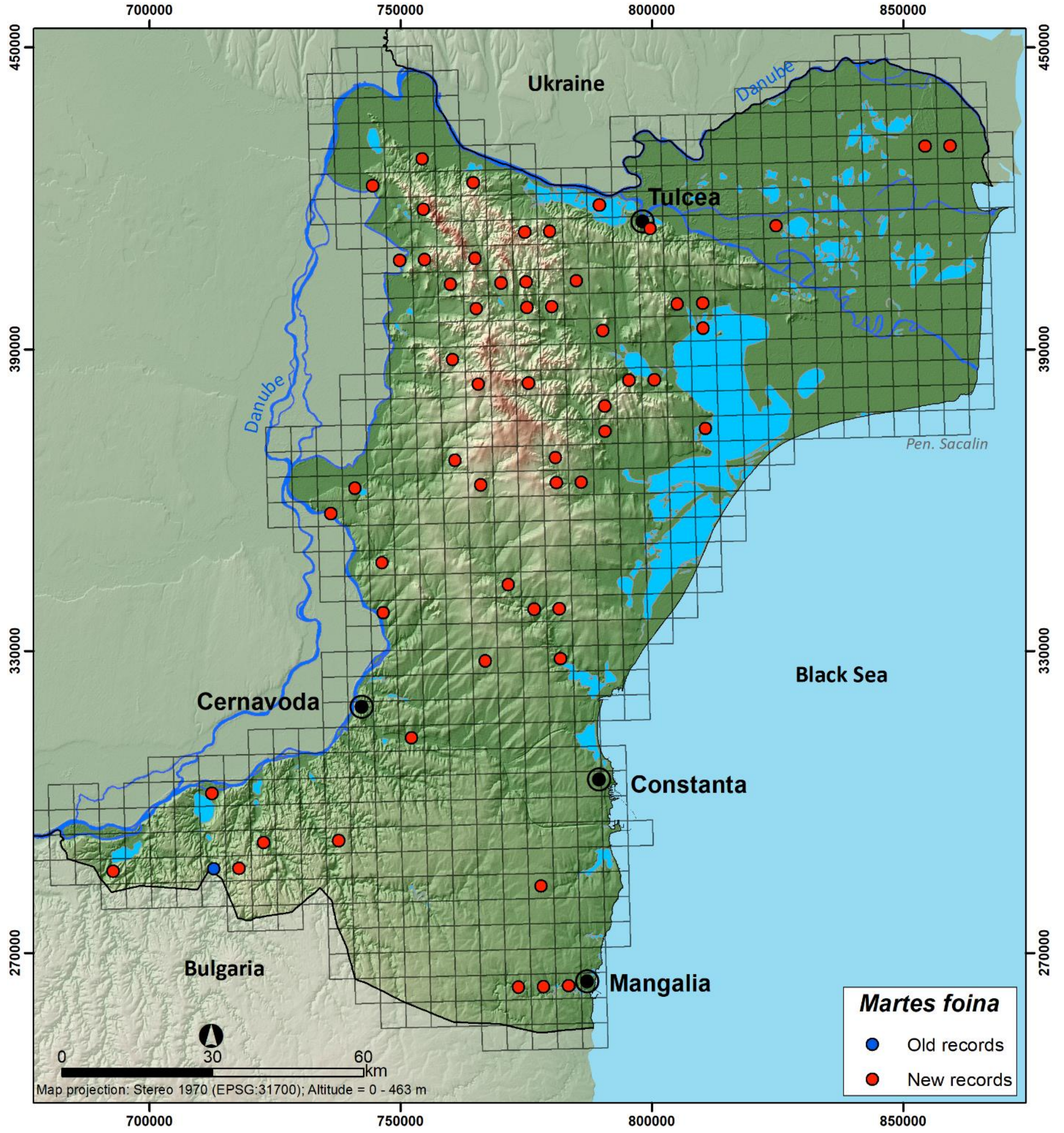

700000
750000
800000
850000
450000
390000
330000
270000
Ukraine
Danube
Tulcea
Pen. Sacalin
Black Sea
Cernavoda
Constanta
Mangalia
Bulgaria
Martes foina
Old records
New records
0
30
60
km
Map projection: Stereo 1970 (EPSG:31700); Altitude = 0 - 463 m

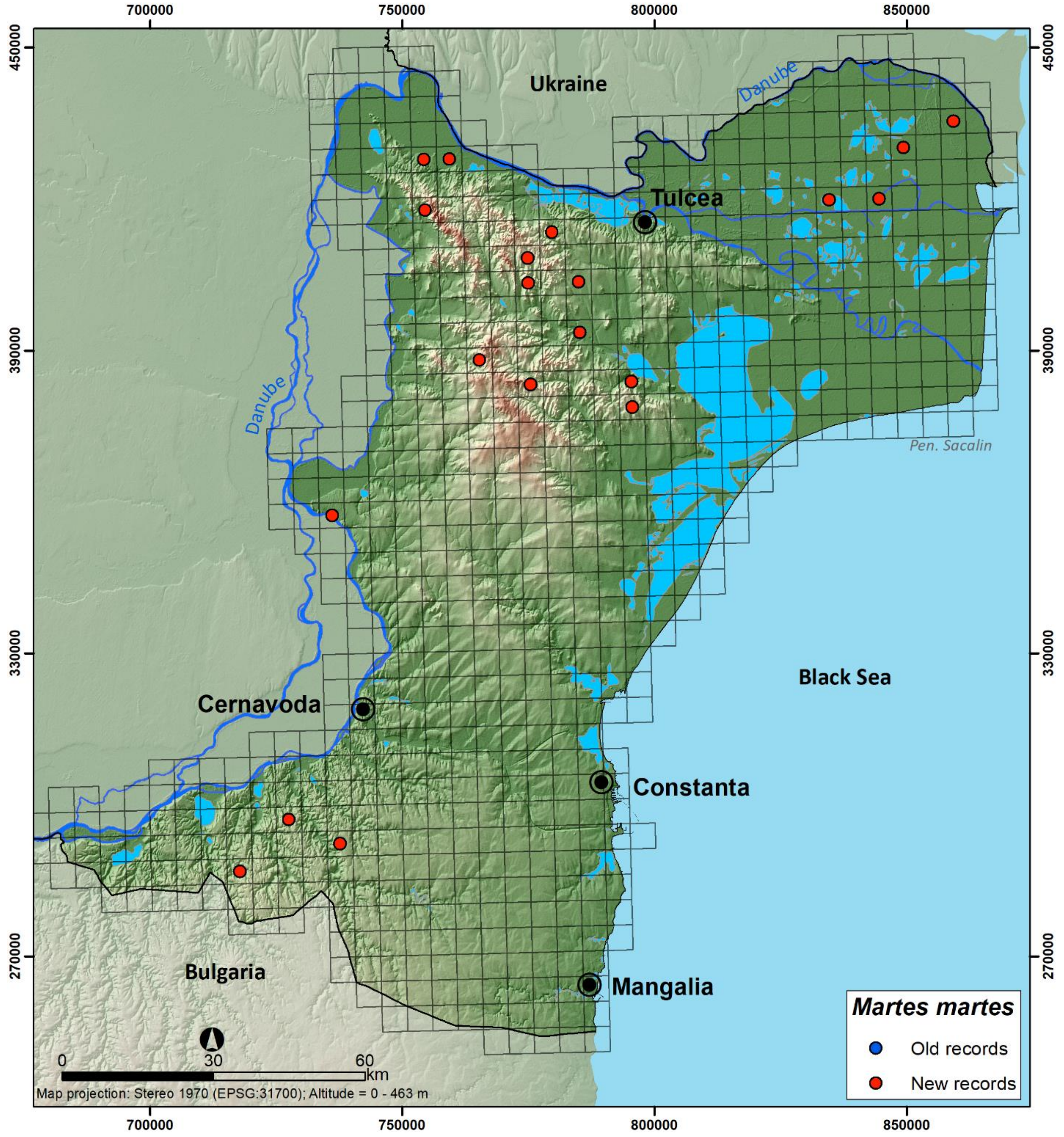
700000
750000
800000
850000
450000
390000
330000
270000
Ukraine
Danube
Tulcea
Pen. Sacalin
Black Sea
Cernavoda
Constanta
Bulgaria
Mangalia
Martes martes
Old records
New records
0
30
60
km
Map projection: Stereo 1970 (EPSG:31700); Altitude = 0 - 463 m

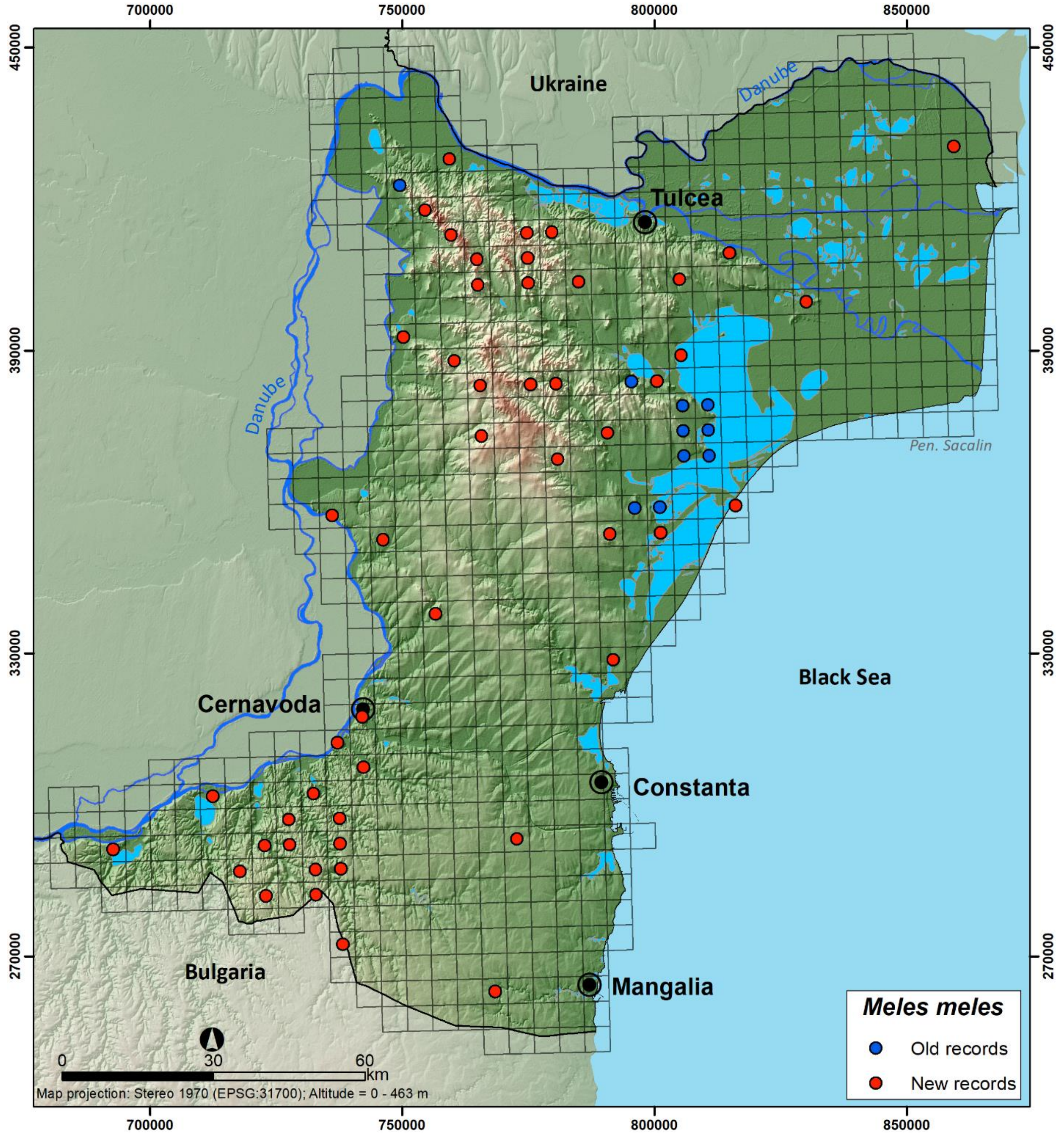
700000
750000
800000
850000
450000
390000
330000
270000
Ukraine
Danube
Tulcea
Pen. Sacalin
Cernavoda
Black Sea
Constanta
Bulgaria
Mangalia
Meles meles
Old records
New records
0
30
60
km
Map projection: Stereo 1970 (EPSG:31700); Altitude = 0 - 463 m

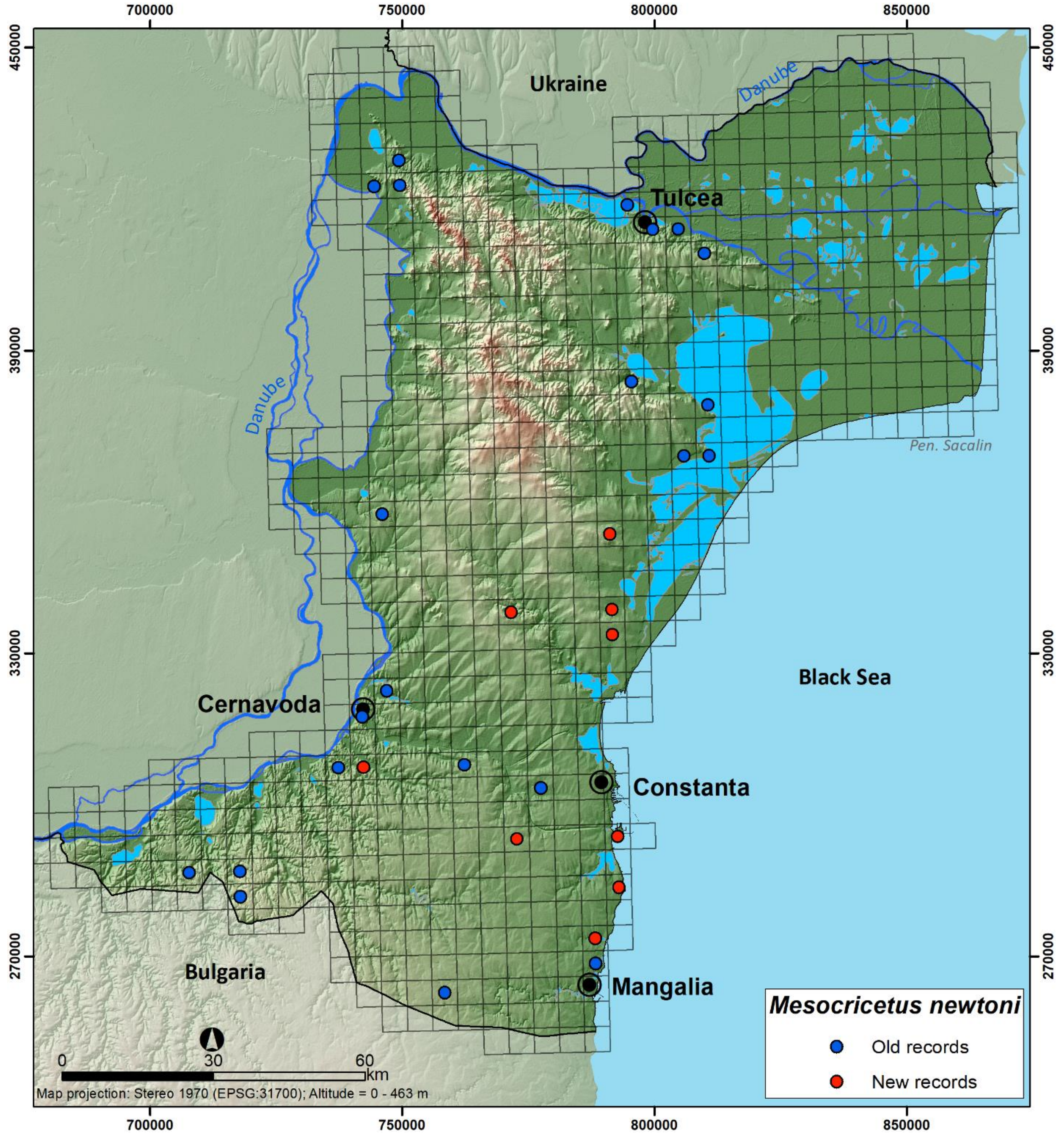

700000
750000
800000
850000
450000
390000
330000
270000
Ukraine
Danube
Tulcea
Pen. Sacalin
Cernavoda
Black Sea
Constanta
Bulgaria
Mangalia
Mesocricetus newtoni
Old records
New records
0
30
60
km
Map projection: Stereo 1970 (EPSG:31700); Altitude = 0 - 463 m

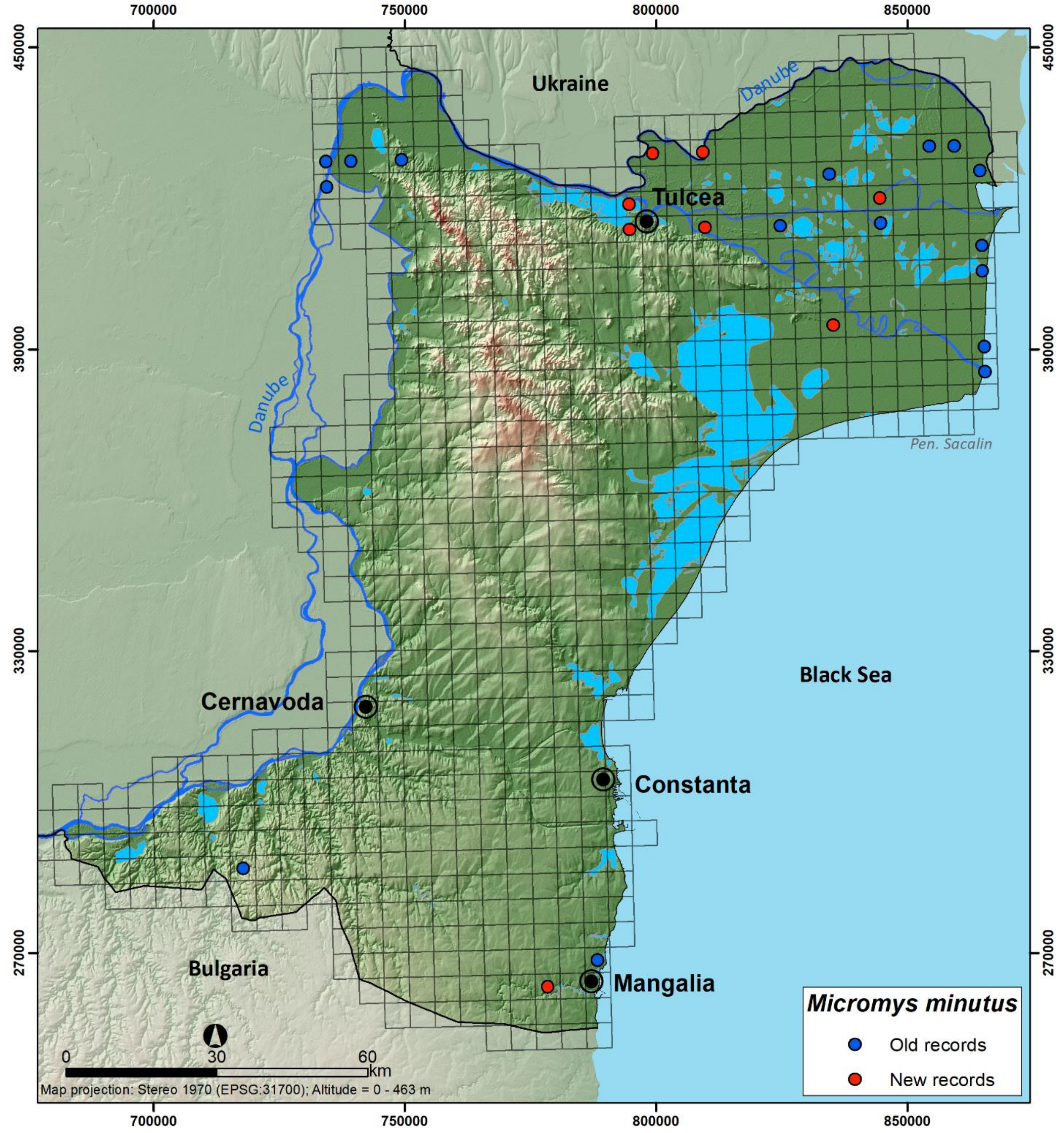

700000
750000
800000
850000
450000
390000
330000
270000
Ukraine
Danube
Tulcea
Pen. Sacalin
Cernavoda
Constanta
Black Sea
Bulgaria
Mangalia
Micromys minutus
Old records
New records
0
30
60
km
Map projection: Stereo 1970 (EPSG:31700); Altitude = 0 - 463 m

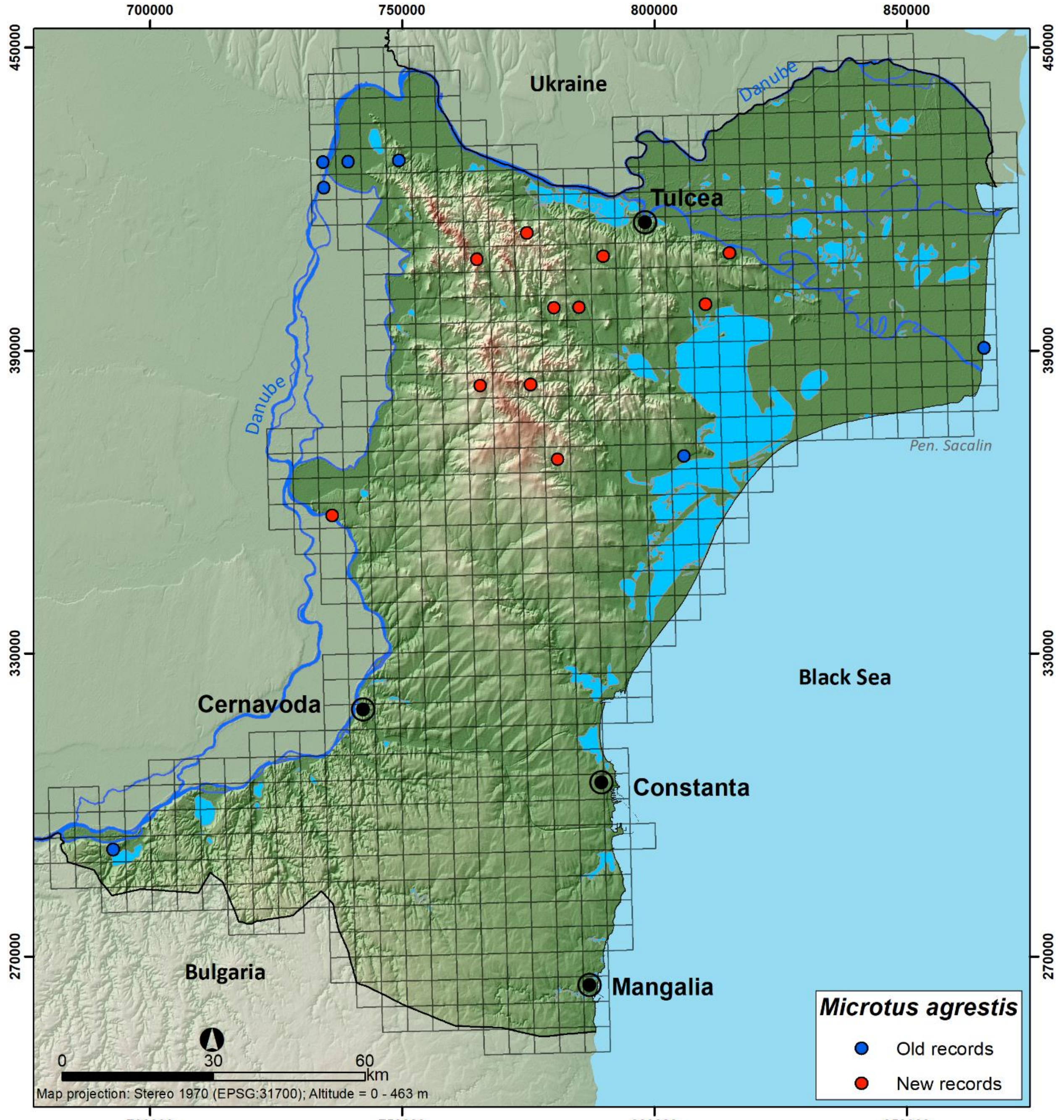
700000
750000
800000
850000
450000
390000
330000
270000
Ukraine
Danube
Tulcea
Danube
Pen. Sacalin
Black Sea
Cernavoda
Constanta
Bulgaria
Mangalia
Microtus agrestis
Old records
New records
0
30
60
km
Map projection: Stereo 1970 (EPSG:31700); Altitude = 0 - 463 m

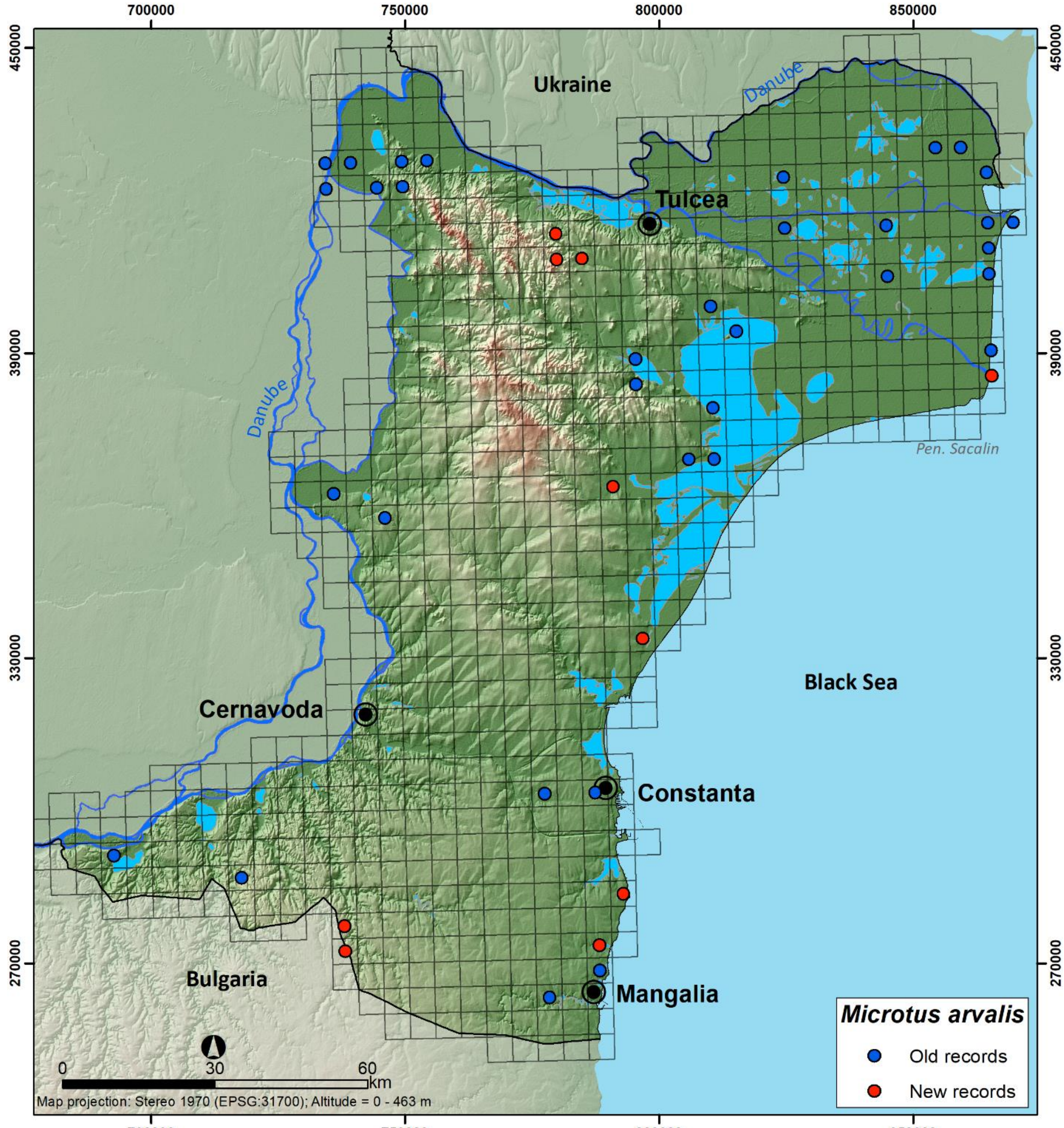
700000
750000
800000
850000
450000
390000
330000
270000
Ukraine
Danube
Tulcea
Pen. Sacalin
Cernavoda
Constanta
Black Sea
Bulgaria
Mangalia
Microtus arvalis
Old records
New records
0
30
60
km
Map projection: Stereo 1970 (EPSG:31700); Altitude = 0 - 463 m

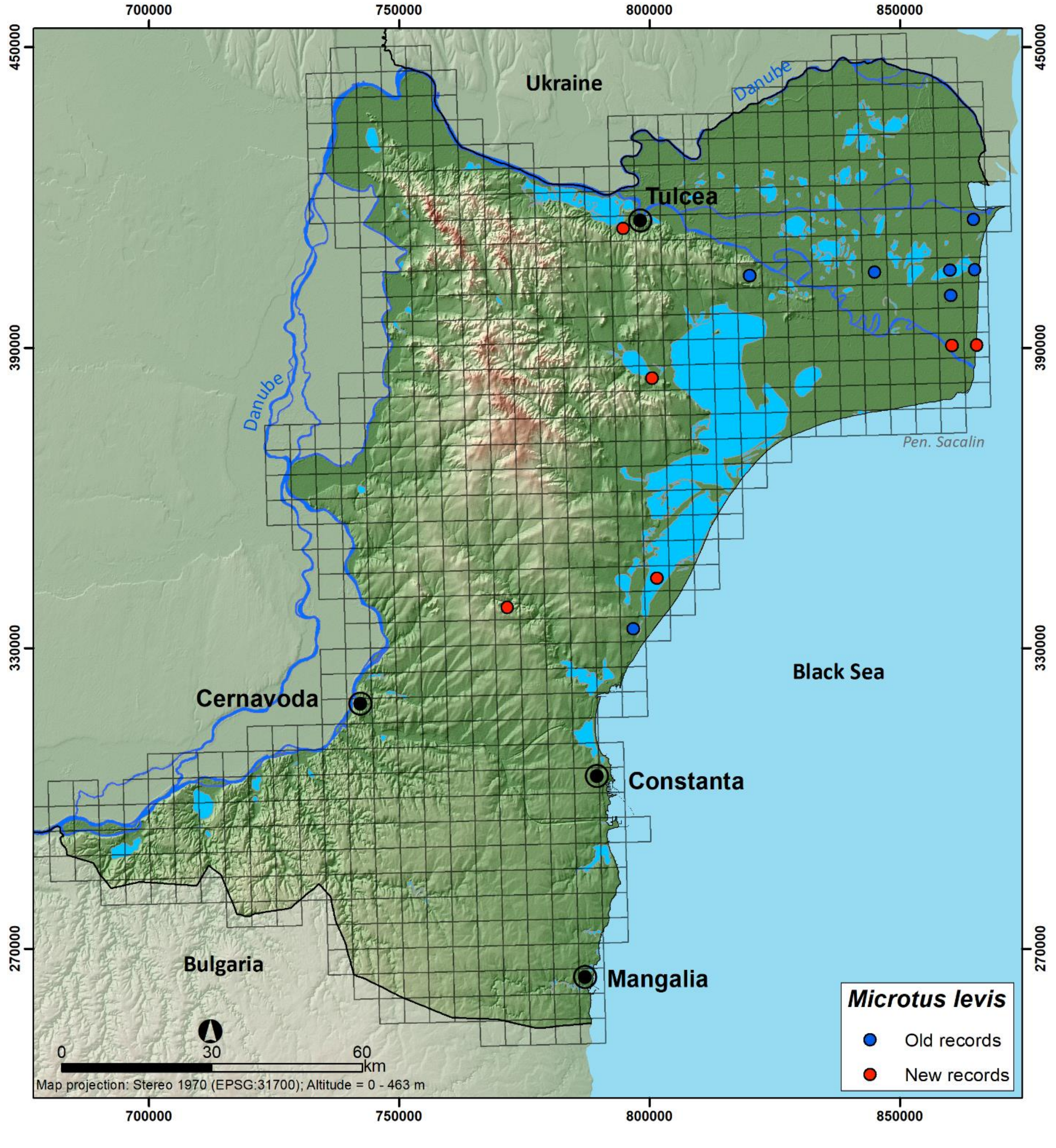
700000
750000
800000
850000
450000
390000
330000
270000
Ukraine
Danube
Tulcea
Danube
Pen. Sacalin
Black Sea
Cernavoda
Constanta
Bulgaria
Mangalia
Microtus levis
Old records
New records
0
30
60
km
Map projection: Stereo 1970 (EPSG:31700); Altitude = 0 - 463 m

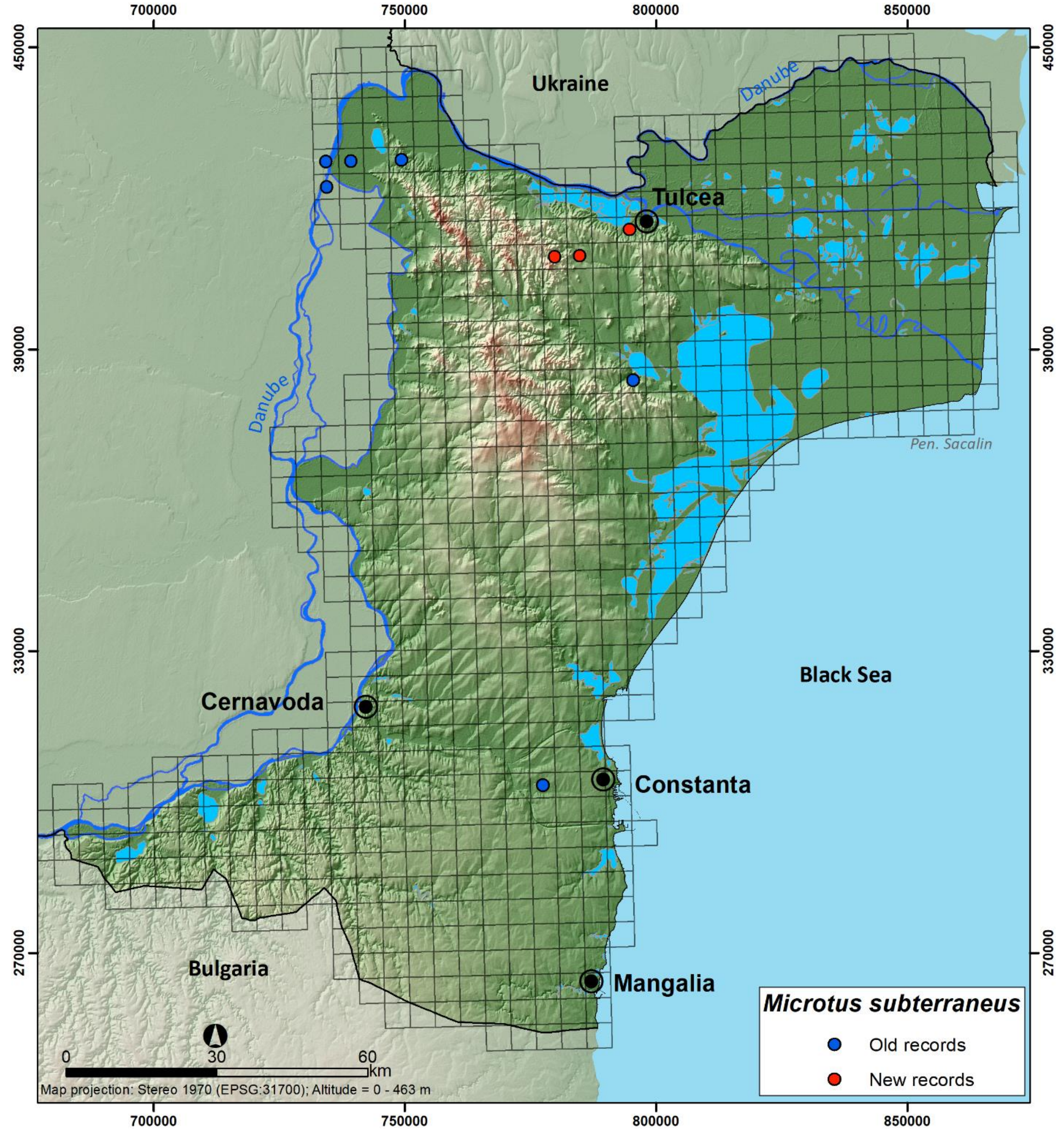

700000
750000
800000
850000
450000
390000
330000
270000
Ukraine
Danube
Tulcea
Pen. Sacalin
Cernavoda
Constanta
Black Sea
Bulgaria
Mangalia
Microtus subterraneus
Old records
New records
0
30
60
km
Map projection: Stereo 1970 (EPSG:31700); Altitude = 0 - 463 m

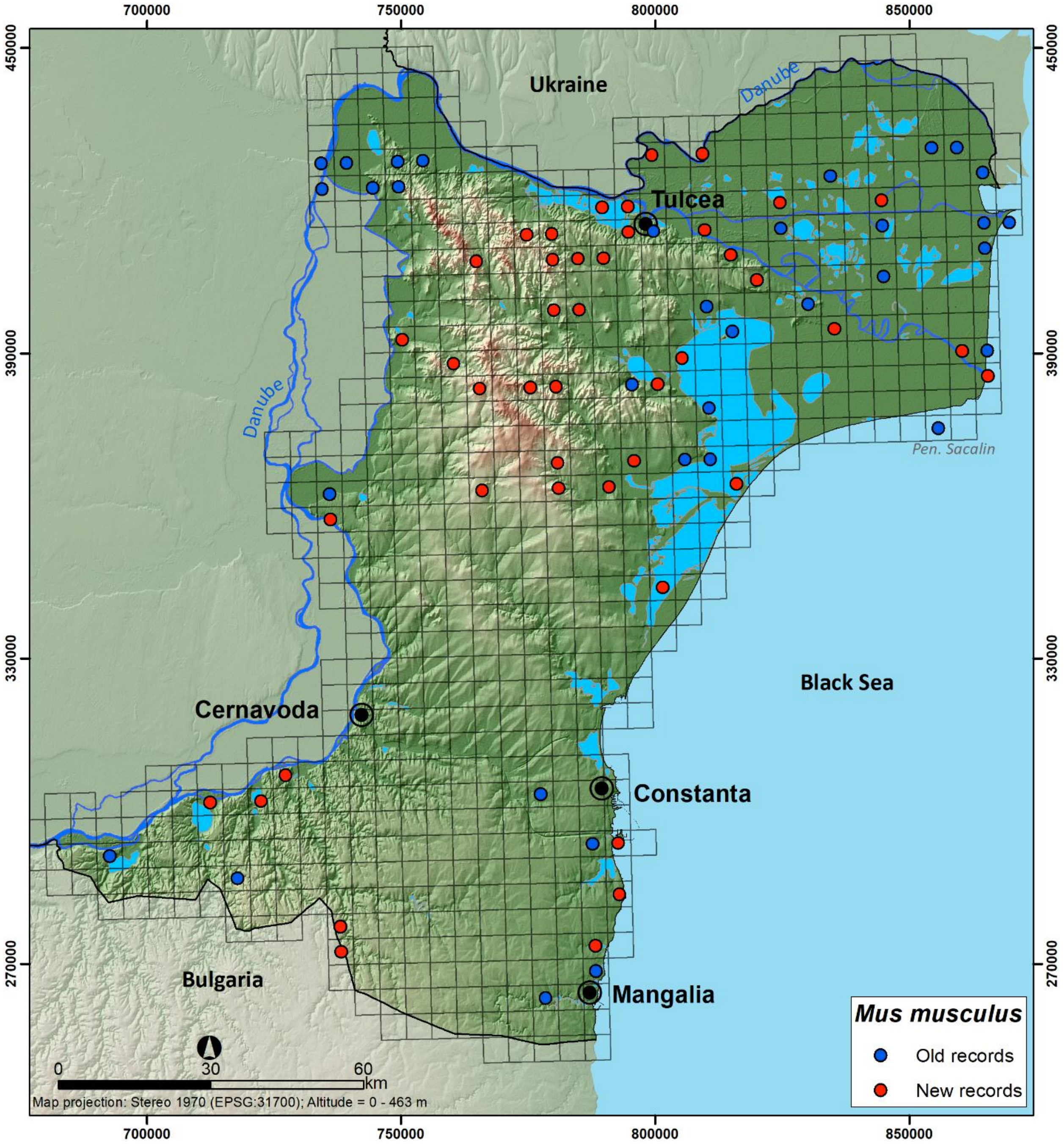
700000
750000
800000
850000
450000
390000
330000
270000
Ukraine
Danube
Tulcea
Pen. Sacalin
Black Sea
Cernavoda
Constanta
Bulgaria
Mangalia
Mus musculus
Old records
New records
0
30
60
km
Map projection: Stereo 1970 (EPSG:31700); Altitude = 0 - 463 m

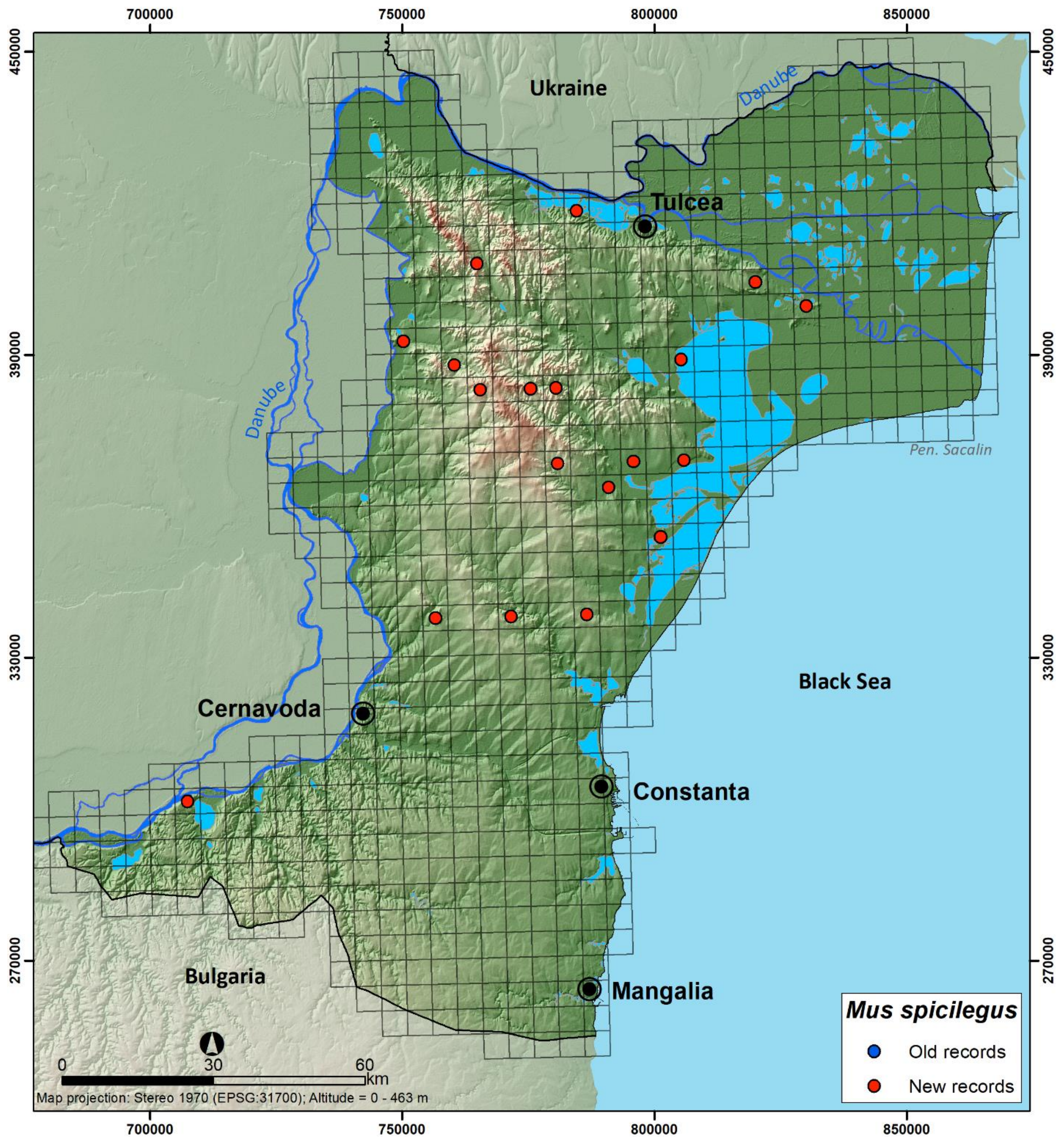
700000
750000
800000
850000
450000
390000
330000
270000
Ukraine
Danube
Tulcea
Pen. Sacalin
Black Sea
Cernavoda
Constanta
Mangalia
Bulgaria
Mus spicilegus
Old records
New records
0
30
60
km
Map projection: Stereo 1970 (EPSG:31700); Altitude = 0 - 463 m

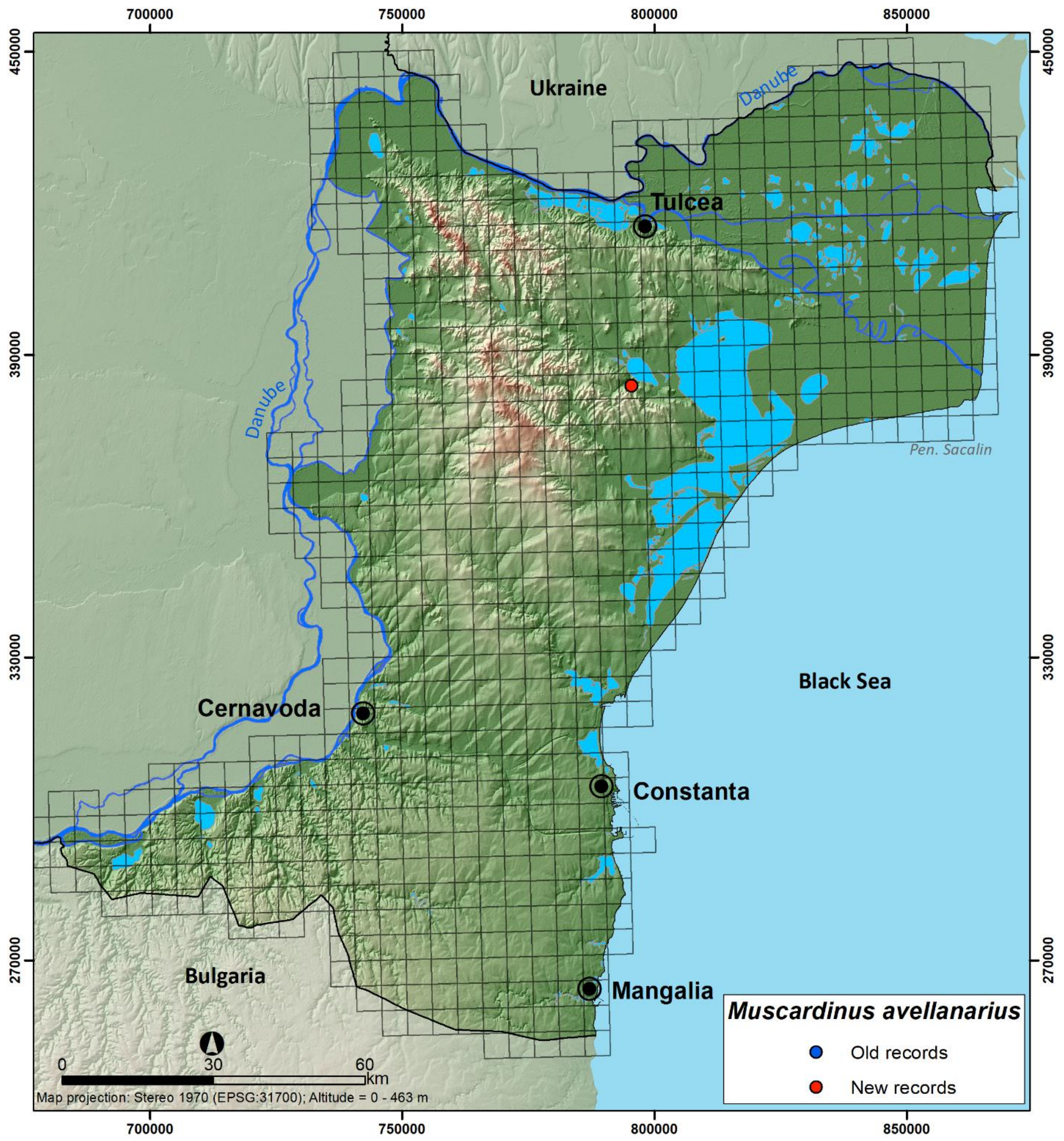

700000
750000
800000
850000
450000
390000
330000
270000
Ukraine
Danube
Tulcea
Danube
Pen. Sacalin
Cernavoda
Black Sea
Constanta
Bulgaria
Mangalia
Muscardinus avellanarius
Old records
New records
0
30
60
km
Map projection: Stereo 1970 (EPSG:31700); Altitude = 0 - 463 m

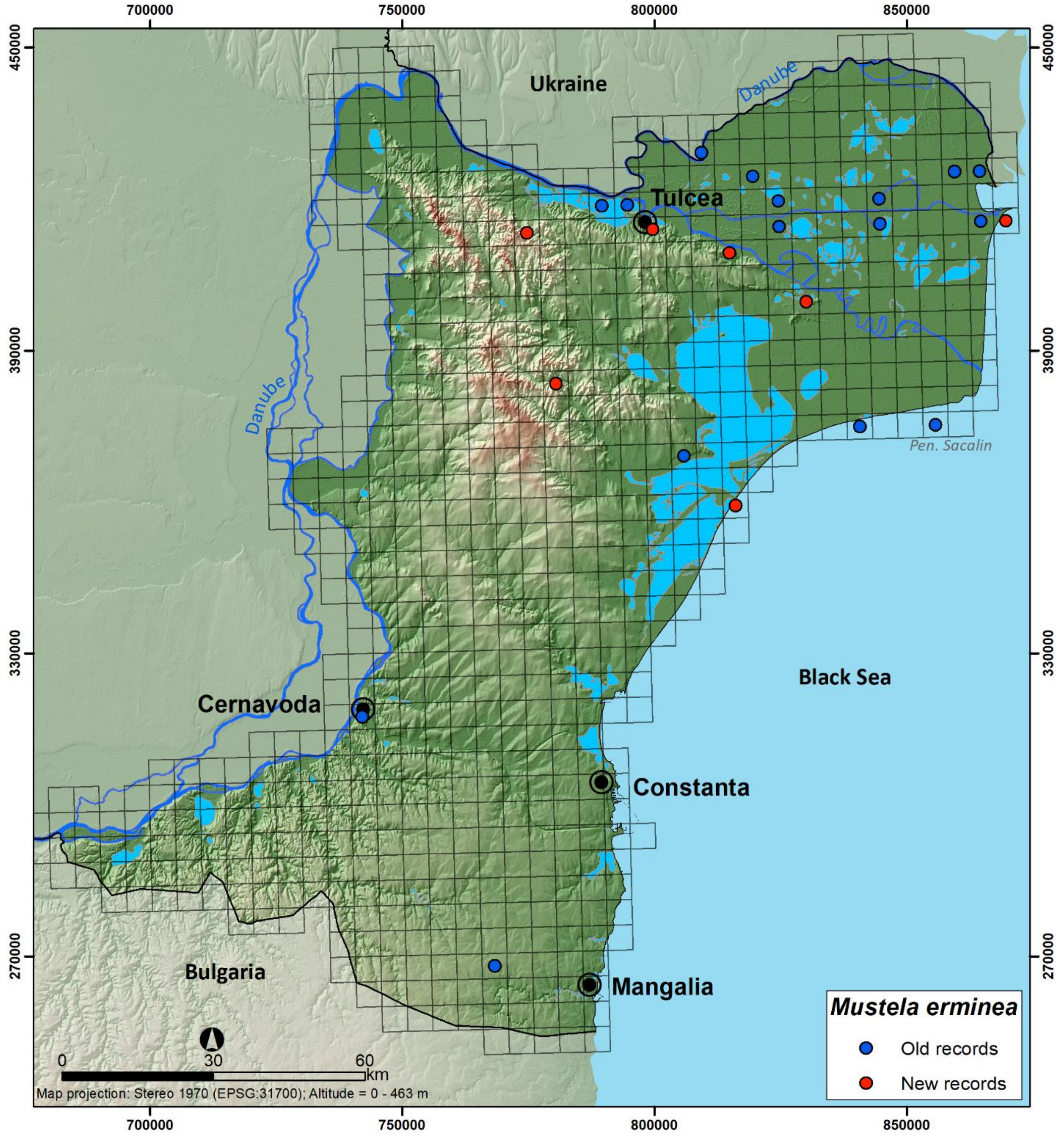
700000
750000
800000
850000
450000
390000
330000
270000
Ukraine
Danube
Tulcea
Danube
Pen. Sacalin
Black Sea
Cernavoda
Constanta
Bulgaria
Mangalia
0
30
60
km
Map projection: Stereo 1970 (EPSG:31700); Altitude = 0 - 463 m
Mustela erminea
Old records
New records

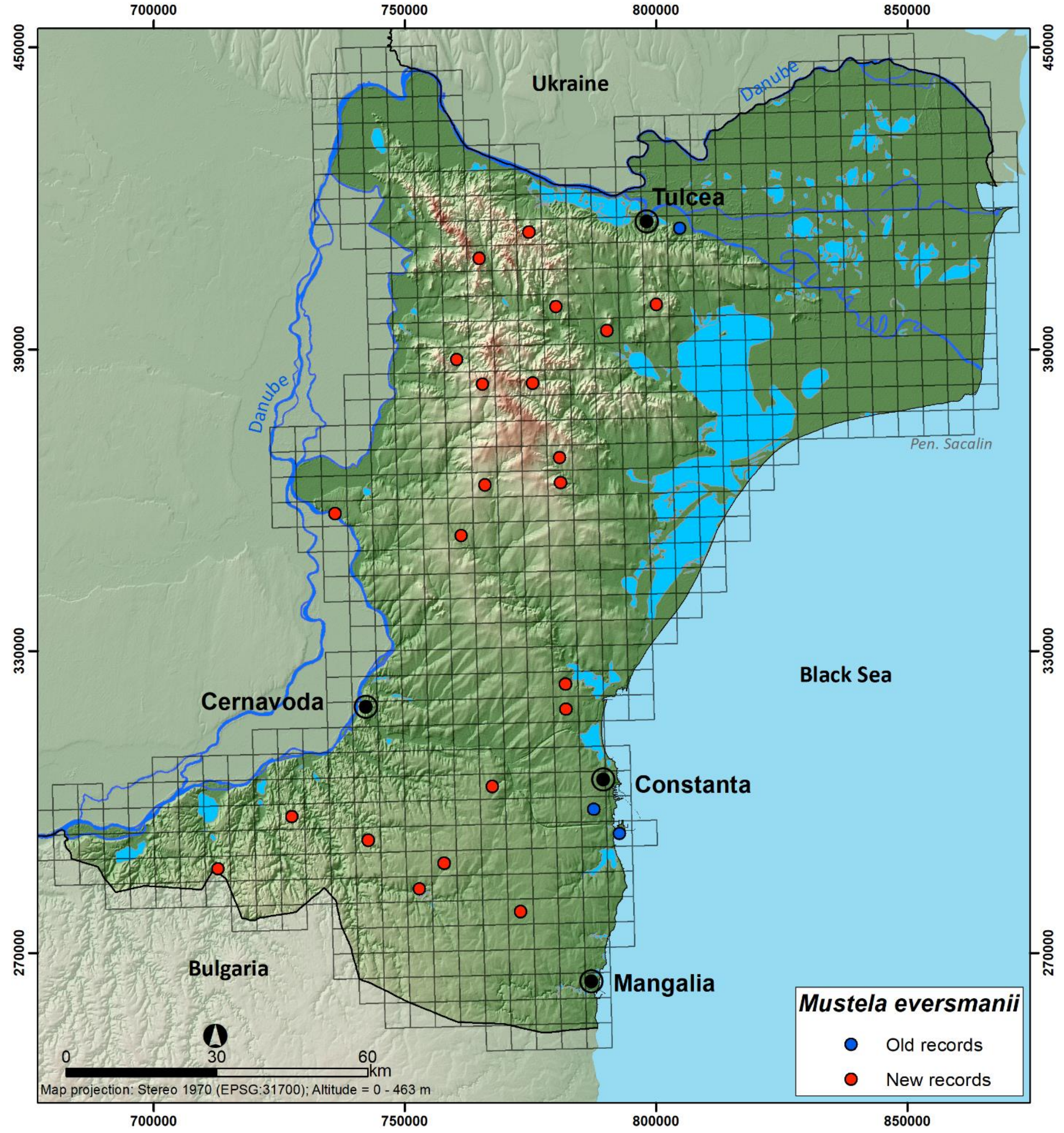

700000
750000
800000
850000
450000
390000
330000
270000
Ukraine
Danube
Tulcea
Danube
Pen. Sacalin
Black Sea
Cernavoda
Constanta
Bulgaria
Mangalia
Mustela eversmanii
Old records
New records
0
30
60
km
Map projection: Stereo 1970 (EPSG:31700); Altitude = 0 - 463 m

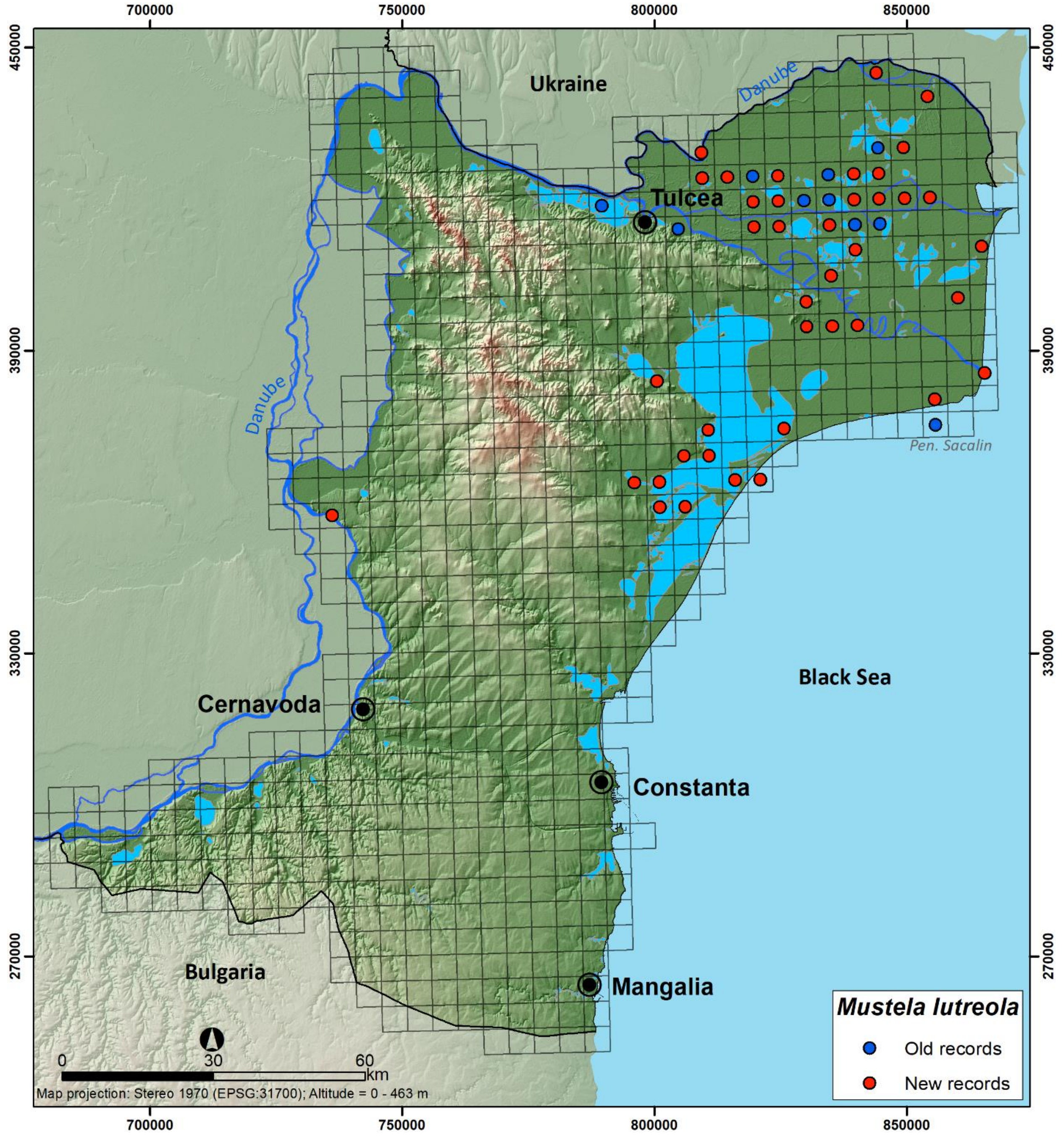
700000
750000
800000
850000
450000
390000
330000
270000
Ukraine
Danube
Tulcea
Pen. Sacalin
Black Sea
Cernavoda
Constanta
Mangalia
Bulgaria
0
30
60
km
Map projection: Stereo 1970 (EPSG:31700); Altitude = 0 - 463 m
Mustela lutreola
Old records
New records

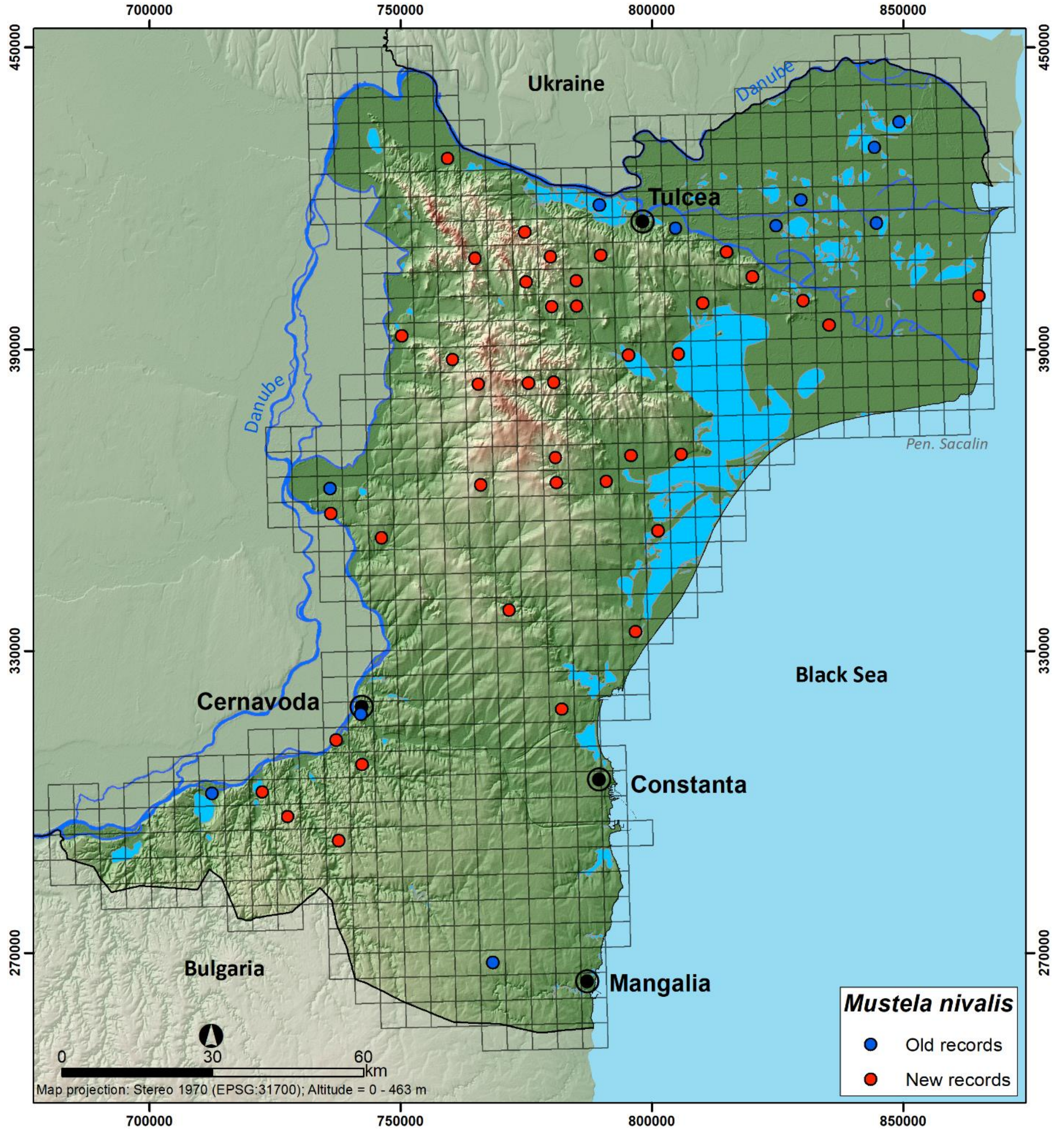
700000
750000
800000
850000
450000
390000
330000
270000
Ukraine
Danube
Tulcea
Pen. Sacalin
Cernavoda
Constanta
Black Sea
Bulgaria
Mangalia
0
30
60
km
Map projection: Stereo 1970 (EPSG:31700); Altitude = 0 - 463 m
Mustela nivalis
Old records
New records

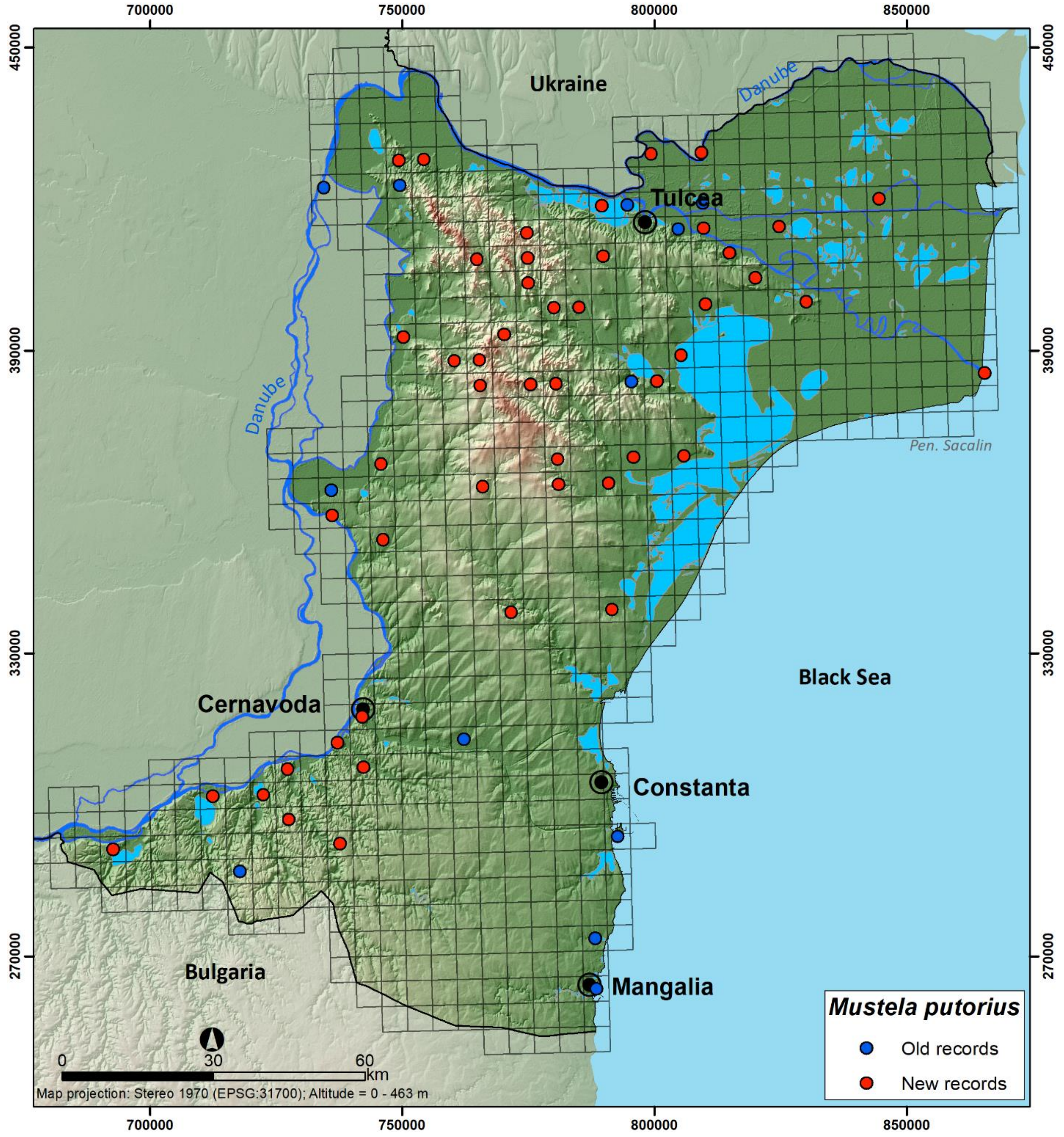
700000
750000
800000
850000
450000
390000
330000
270000
Ukraine
Danube
Tulcea
Danube
Pen. Sacalin
Black Sea
Cernavoda
Constanta
Bulgaria
Mangalia
Mustela putorius
Old records
New records
0
30
60
km
Map projection: Stereo 1970 (EPSG:31700); Altitude = 0 - 463 m

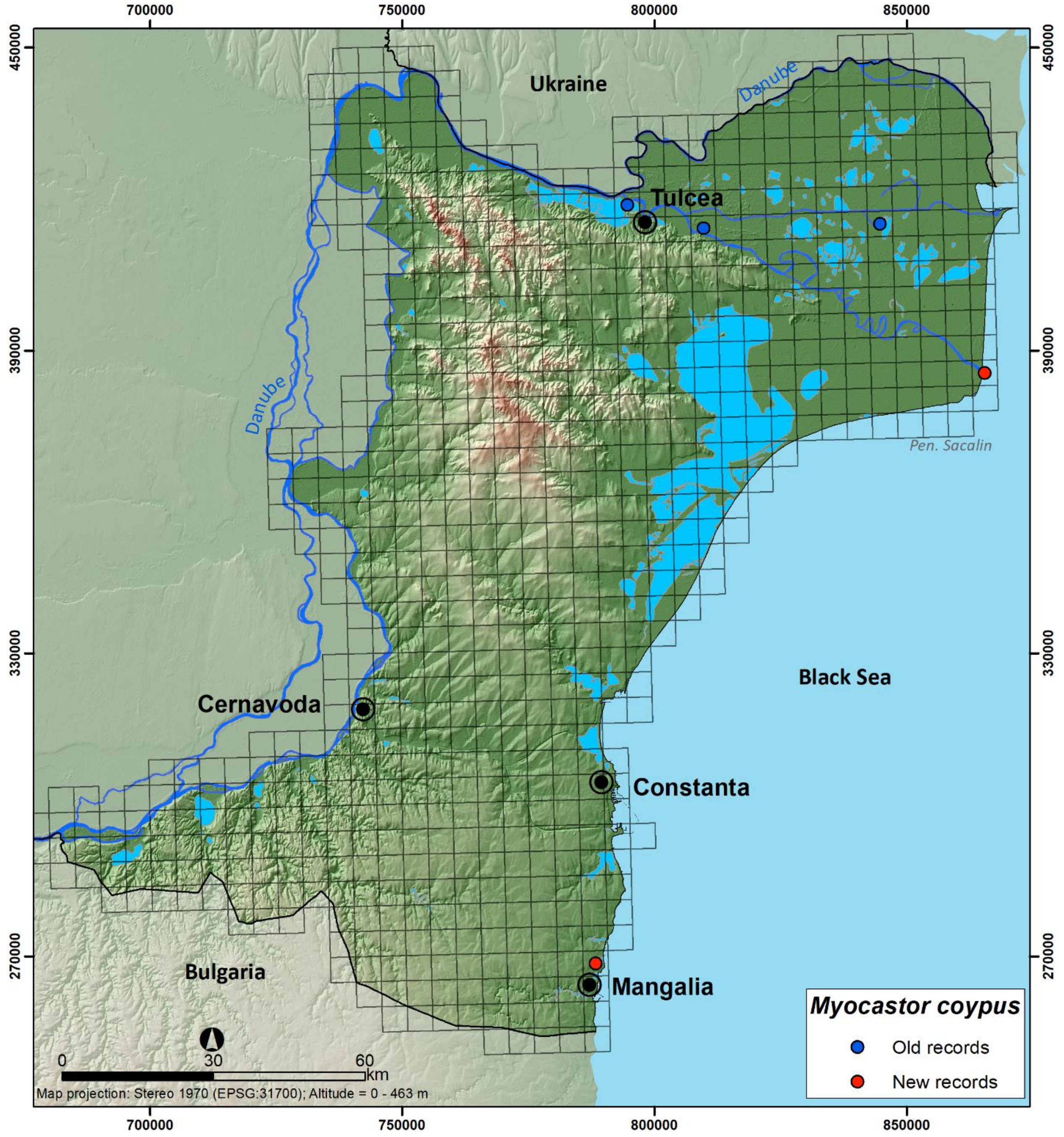
700000
750000
800000
850000
450000
390000
330000
270000
Ukraine
Danube
Tulcea
Pen. Sacalin
Black Sea
Cernavoda
Constanta
Bulgaria
Mangalia
Myocastor coypus
Old records
New records
0
30
60
km
Map projection: Stereo 1970 (EPSG:31700); Altitude = 0 - 463 m

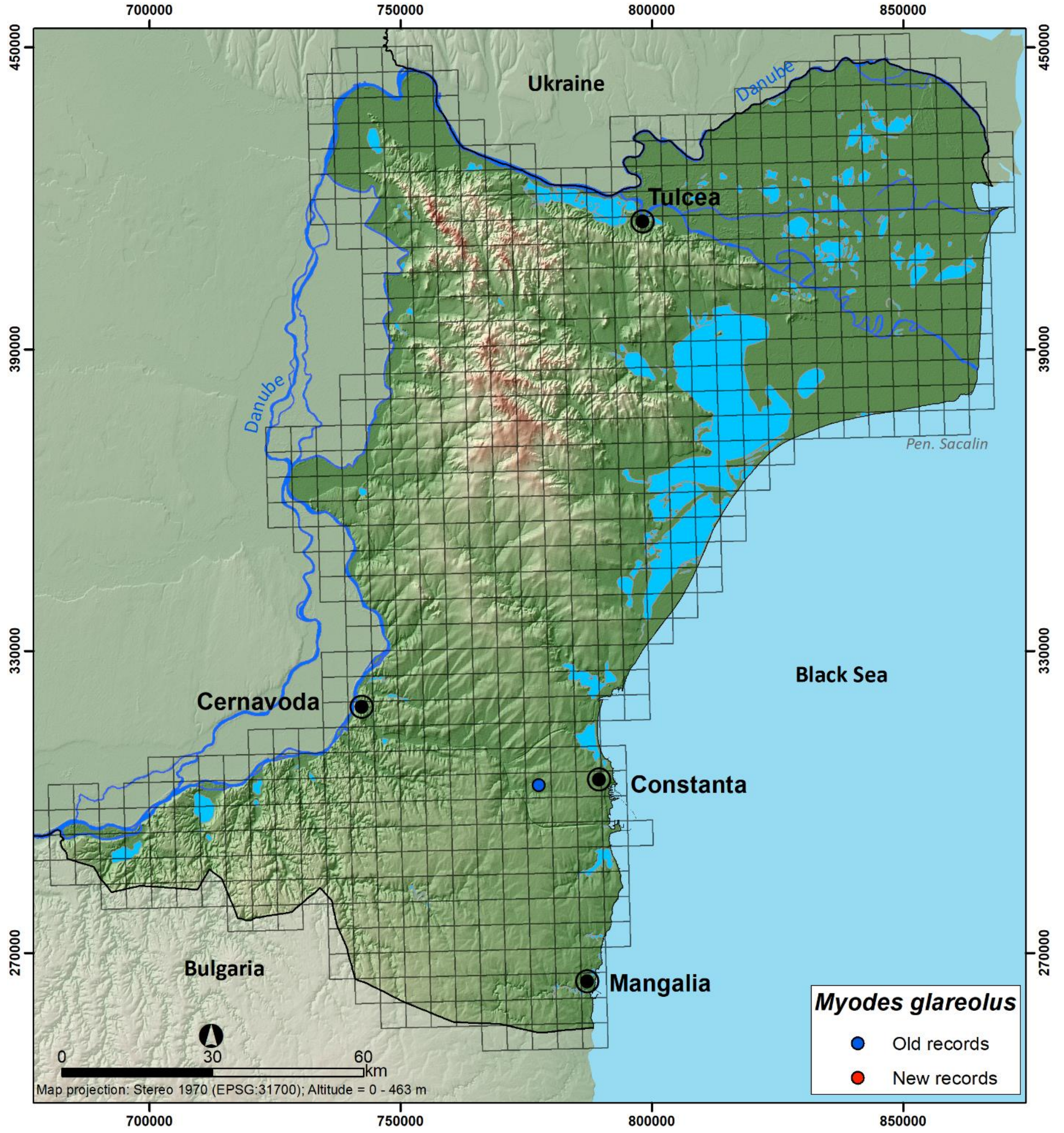
700000
750000
800000
850000
450000
390000
330000
270000
Ukraine
Danube
Tulcea
Danube
Pen. Sacalin
Black Sea
Cernavoda
Constanta
Bulgaria
Mangalia
Myodes glareolus
Old records
New records
0
30
60
km
Map projection: Stereo 1970 (EPSG:31700); Altitude = 0 - 463 m

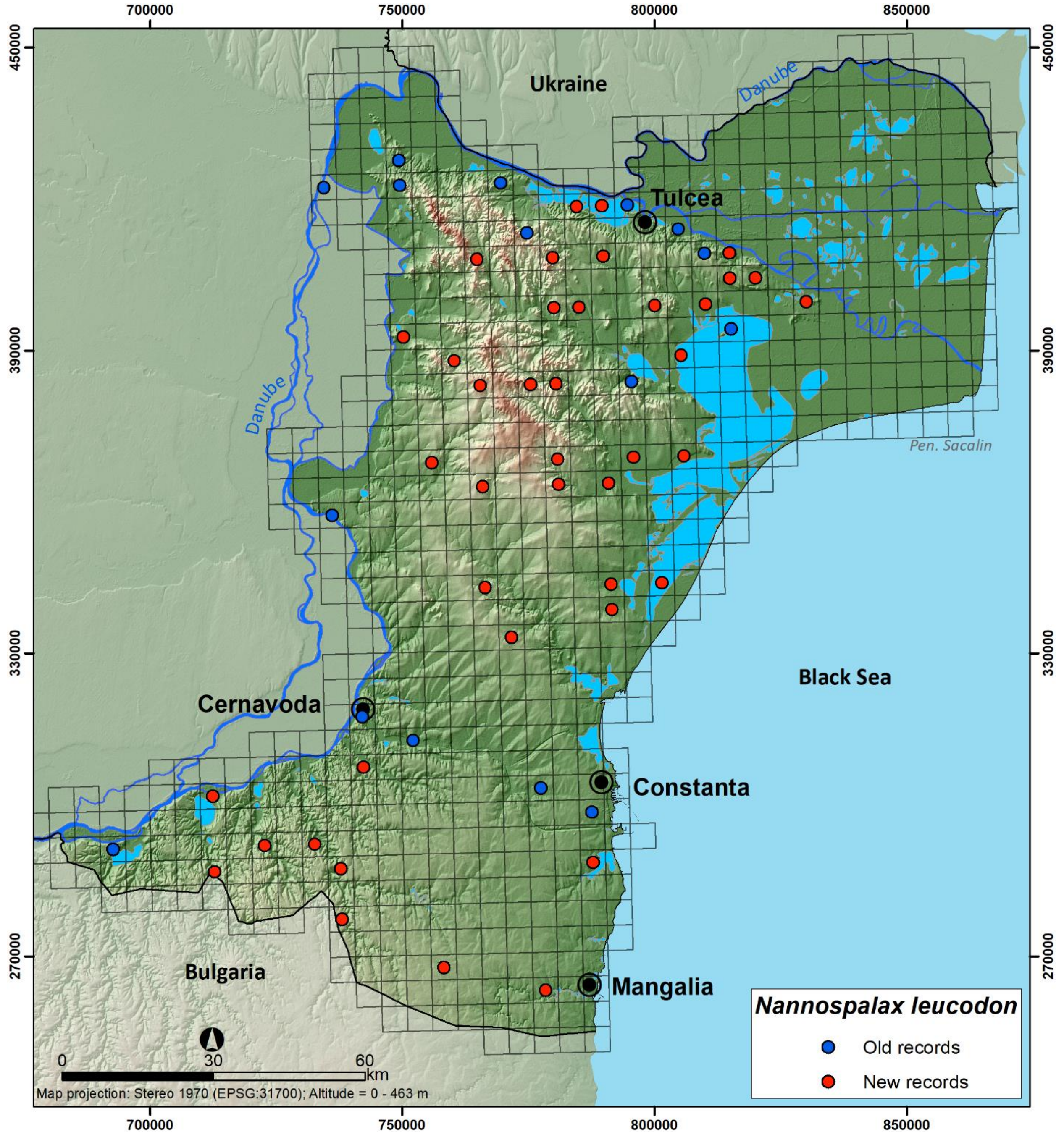

700000
750000
800000
850000
450000
390000
330000
270000
Ukraine
Danube
Tulcea
Pen. Sacalin
Black Sea
Cernavoda
Constanta
Mangalia
Bulgaria
0
30
60
km
Map projection: Stereo 1970 (EPSG:31700); Altitude = 0 - 463 m
Nannospalax leucodon
Old records
New records

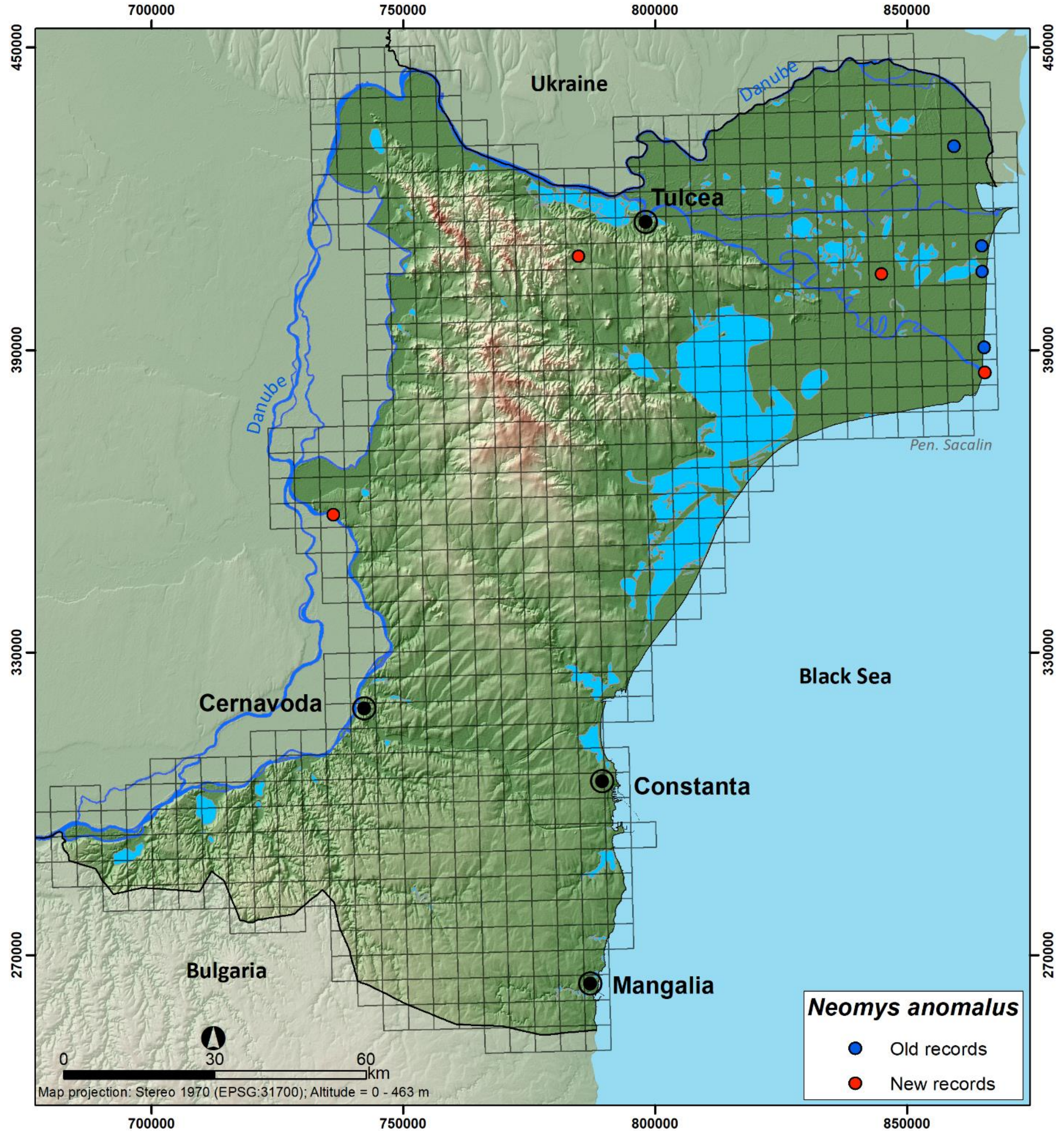
700000
750000
800000
850000
450000
390000
330000
270000
Ukraine
Danube
Tulcea
Pen. Sacalin
Cernavoda
Constanta
Black Sea
Bulgaria
Mangalia
Neomys anomalus
Old records
New records
0
30
60
km
Map projection: Stereo 1970 (EPSG:31700); Altitude = 0 - 463 m

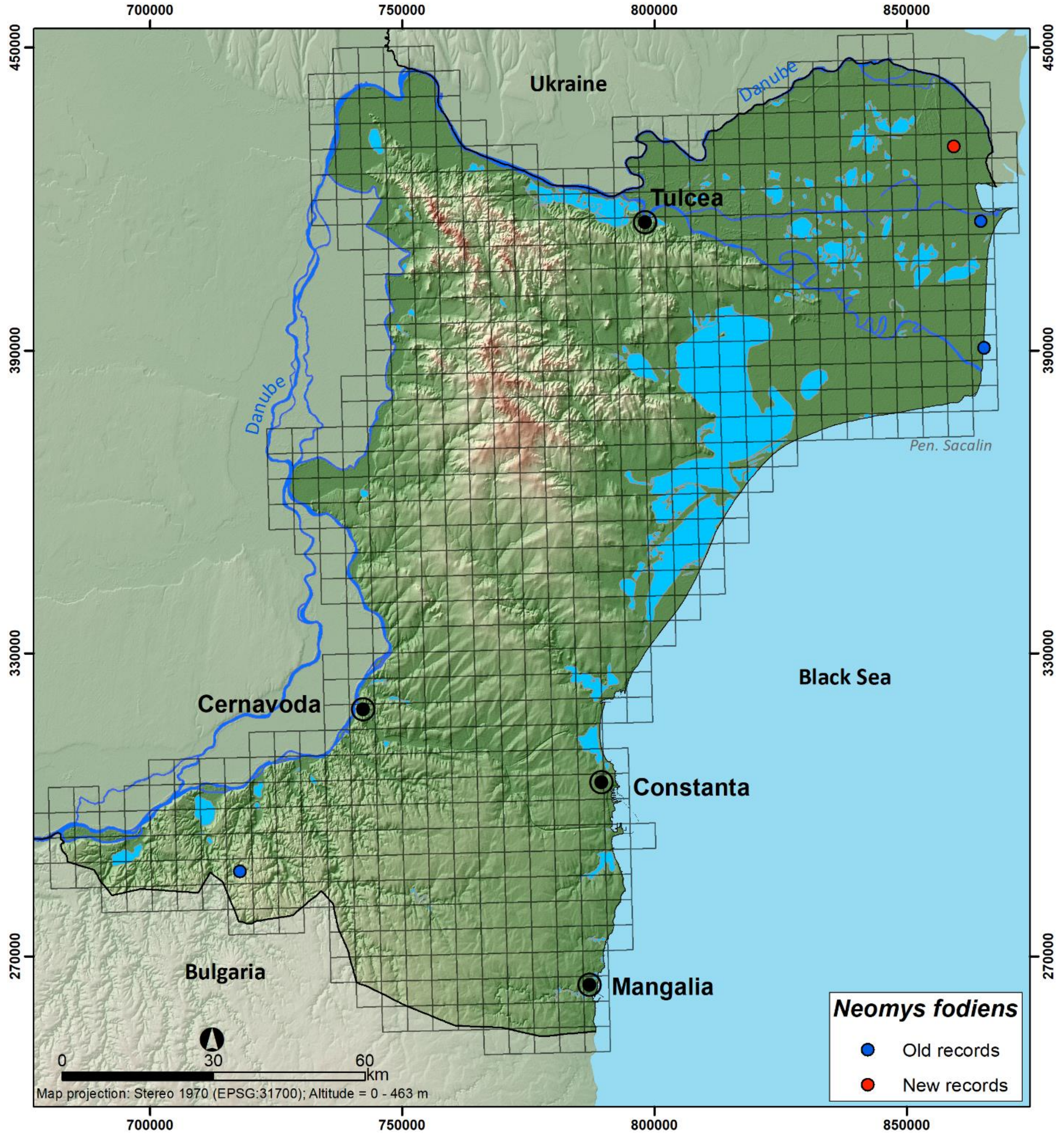
700000
750000
800000
850000
450000
390000
330000
270000
Ukraine
Danube
Tulcea
Pen. Sacalin
Danube
Black Sea
Cernavoda
Constanta
Bulgaria
Mangalia
Neomys fodiens
Old records
New records
0
30
60
km
Map projection: Stereo 1970 (EPSG:31700); Altitude = 0 - 463 m

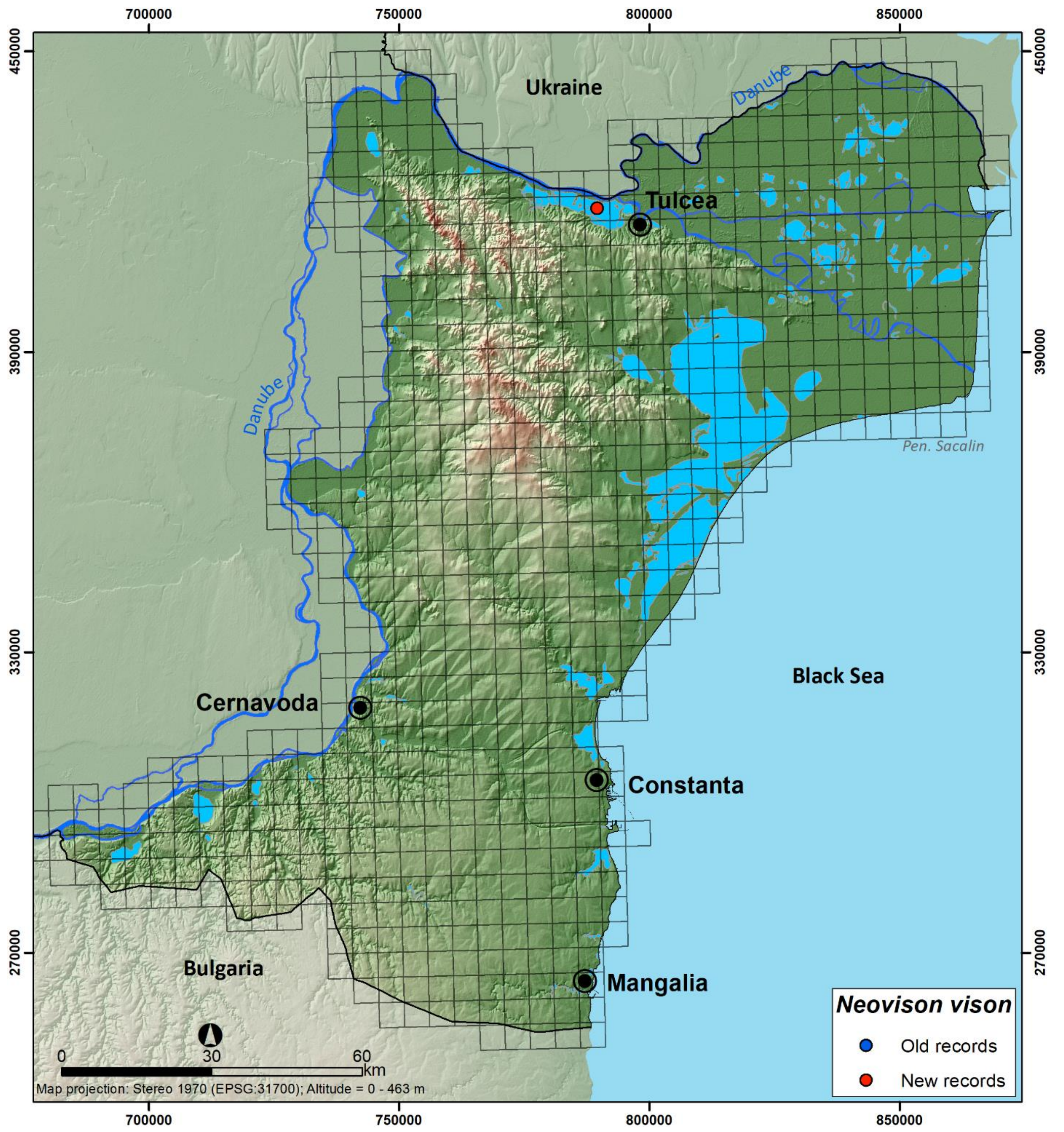

700000
750000
800000
850000
450000
390000
330000
270000
Ukraine
Danube
Tulcea
Danube
Pen. Sacalin
Black Sea
Cernavoda
Constanta
Bulgaria
Mangalia
Neovison vison
Old records
New records
0
30
60
km
Map projection: Stereo 1970 (EPSG:31700); Altitude = 0 - 463 m

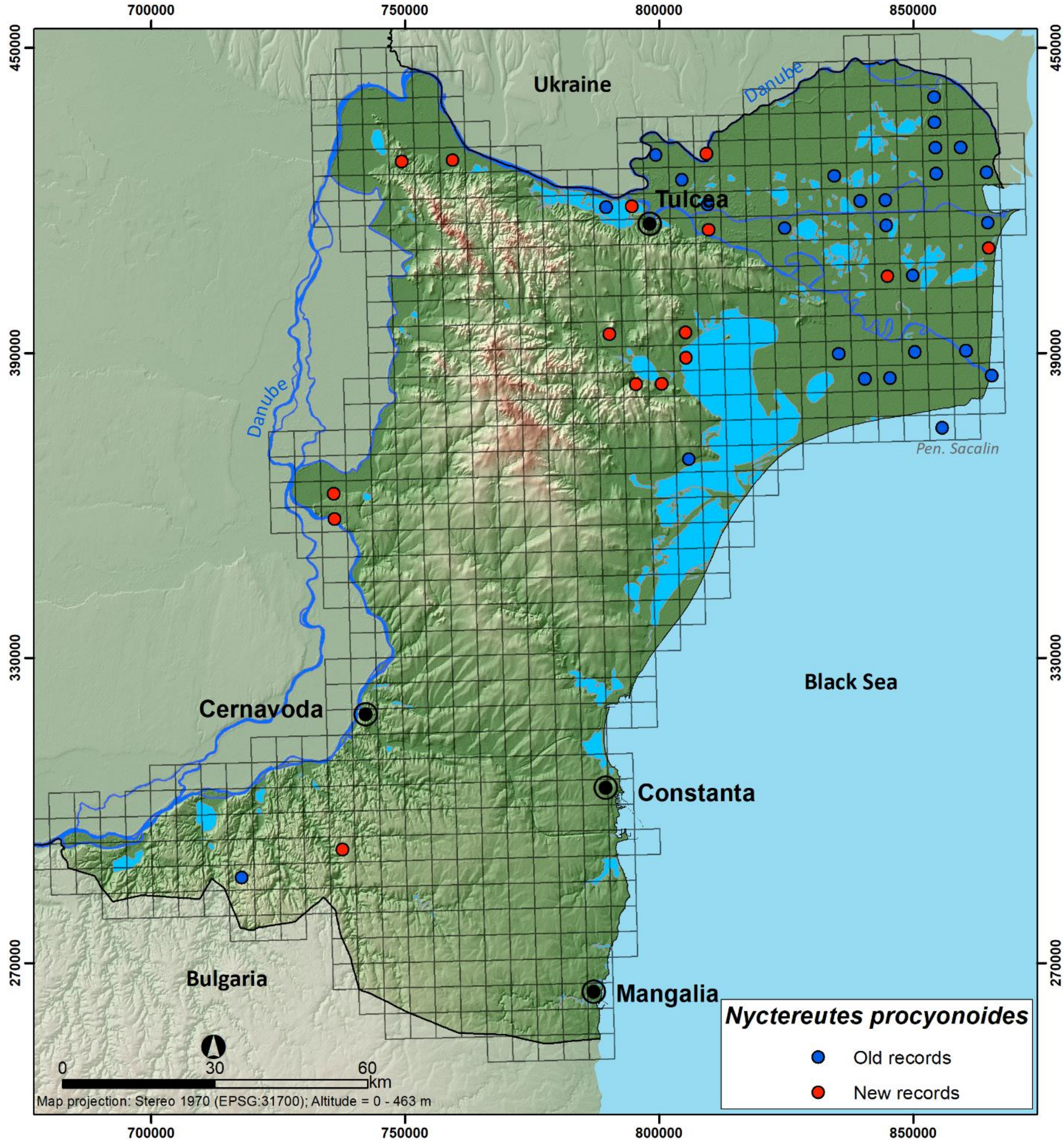
700000
750000
800000
850000
450000
390000
330000
270000
Ukraine
Danube
Tulcea
Pen. Sacalin
Cernavoda
Constanta
Black Sea
Bulgaria
Mangalia
Nyctereutes procyonoides
Old records
New records
0
30
60
km
Map projection: Stereo 1970 (EPSG:31700); Altitude = 0 - 463 m

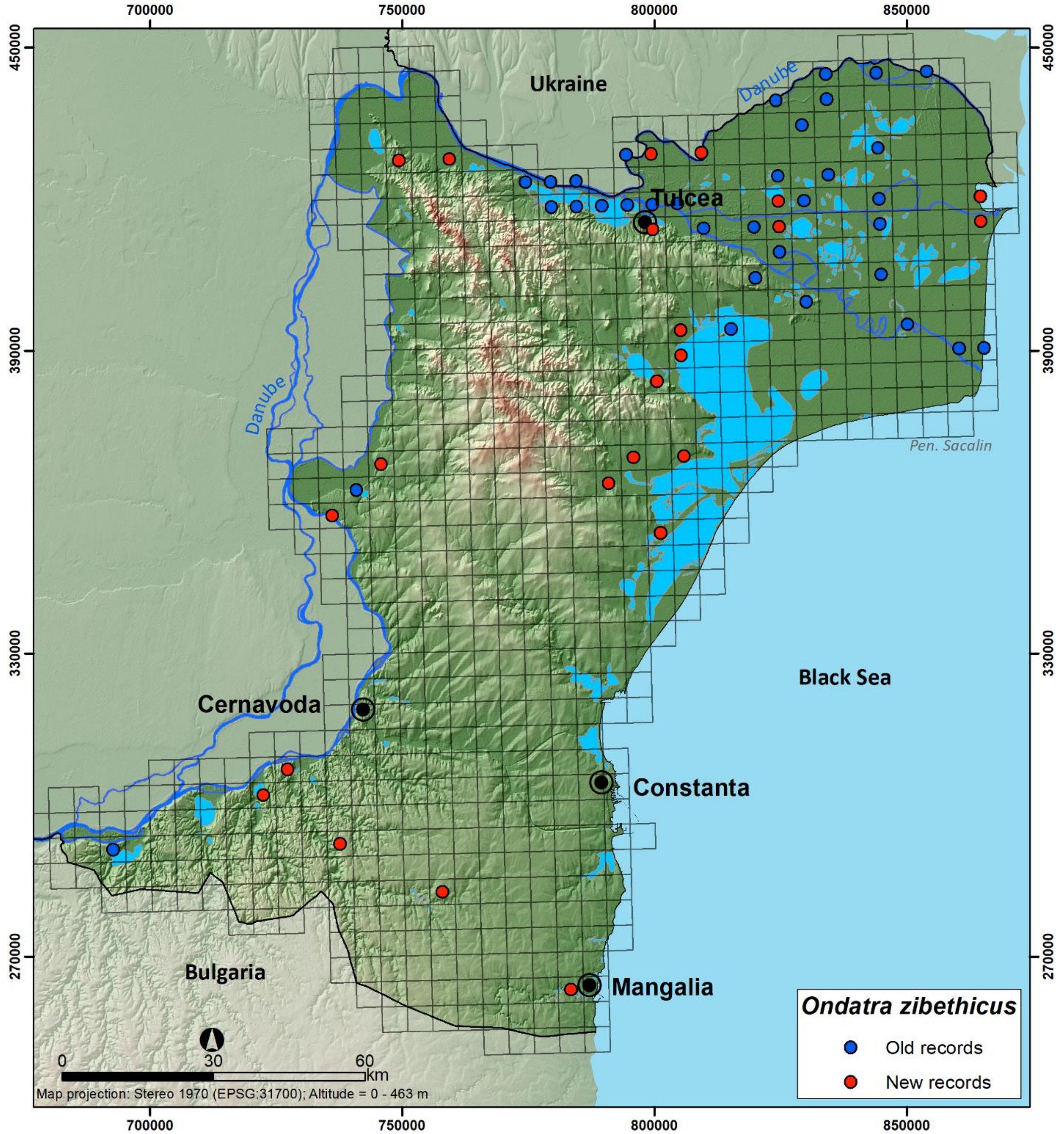
Ukraine
Danube
Tulcea
Cernavoda
Constanta
Mangalia
Bulgaria
Black Sea
Pen. Sacalin
700000
750000
800000
850000
450000
390000
330000
270000
0
30
60
km
Map projection: Stereo 1970 (EPSG:31700); Altitude = 0 - 463 m
Ondatra zibethicus
Old records
New records

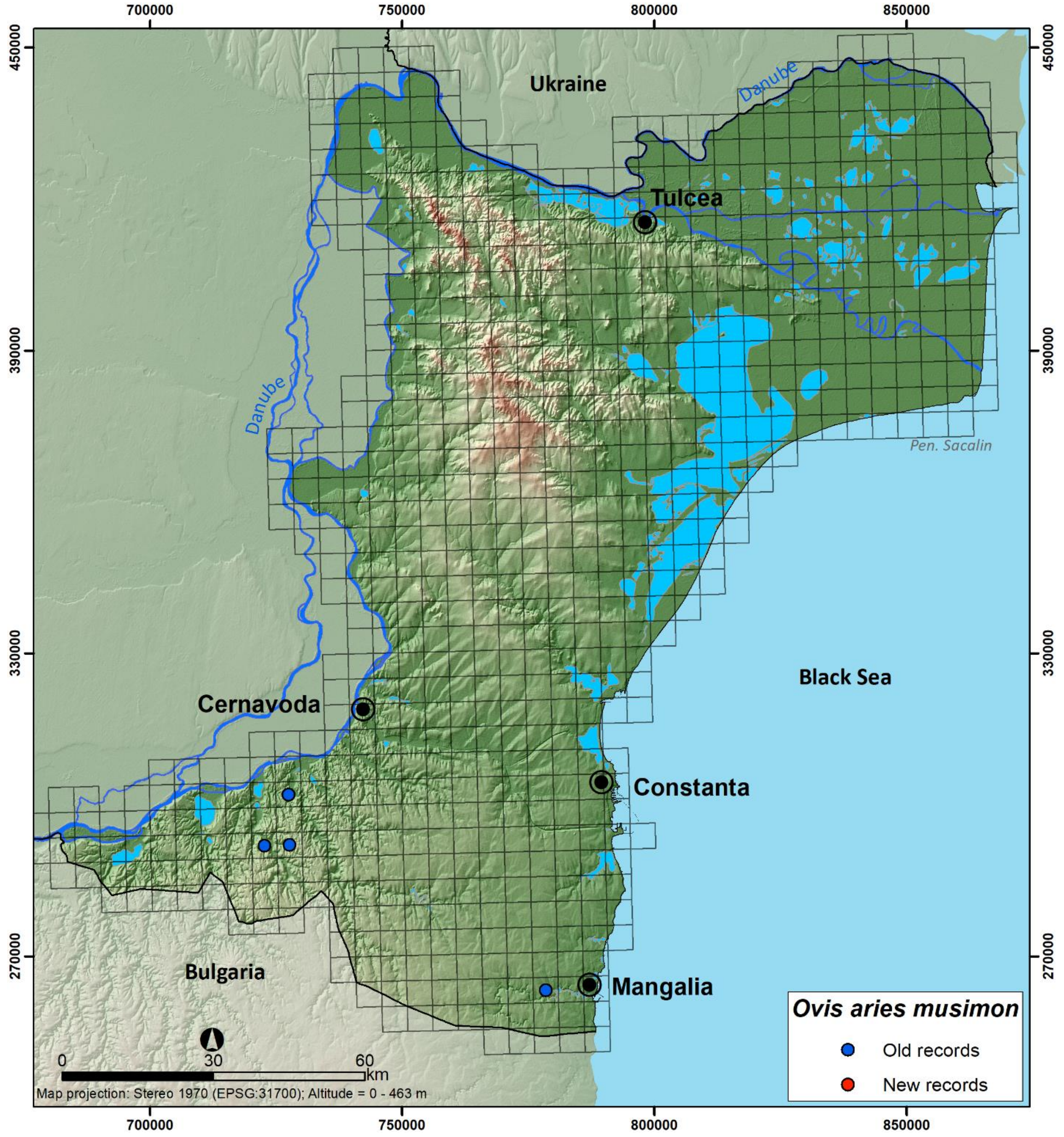
700000
750000
800000
850000
450000
390000
330000
270000
Ukraine
Danube
Tulcea
Danube
Pen. Sacalin
Black Sea
Cernavoda
Constanta
Bulgaria
Mangalia
Ovis aries musimon
Old records
New records
0
30
60
km
Map projection: Stereo 1970 (EPSG:31700); Altitude = 0 - 463 m

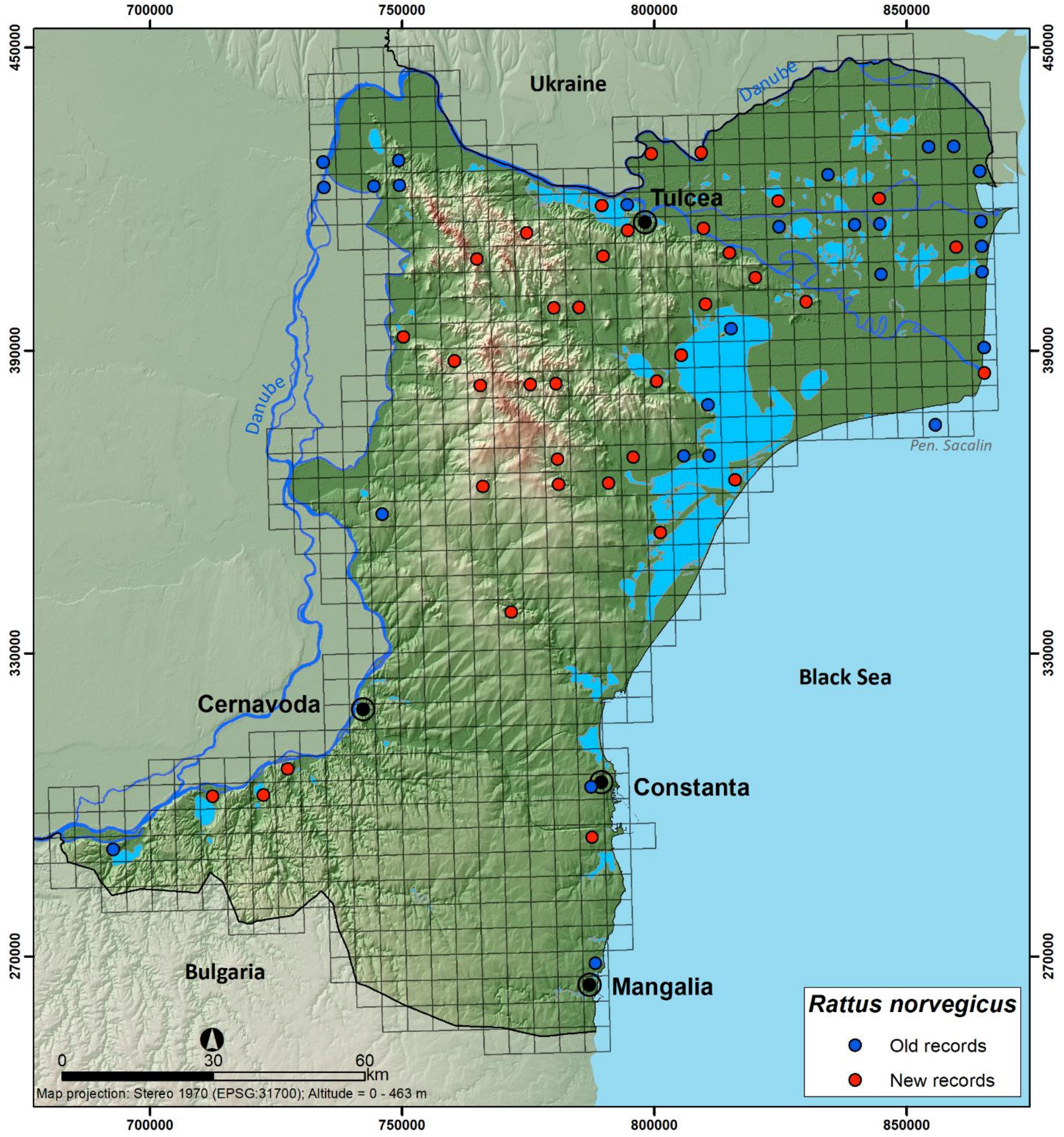
700000
750000
800000
850000
450000
390000
330000
270000
Ukraine
Danube
Tulcea
Pen. Sacalin
Black Sea
Cernavoda
Constanta
Mangalia
Bulgaria
Rattus norvegicus
Old records
New records
0
30
60
km
Map projection: Stereo 1970 (EPSG:31700); Altitude = 0 - 463 m

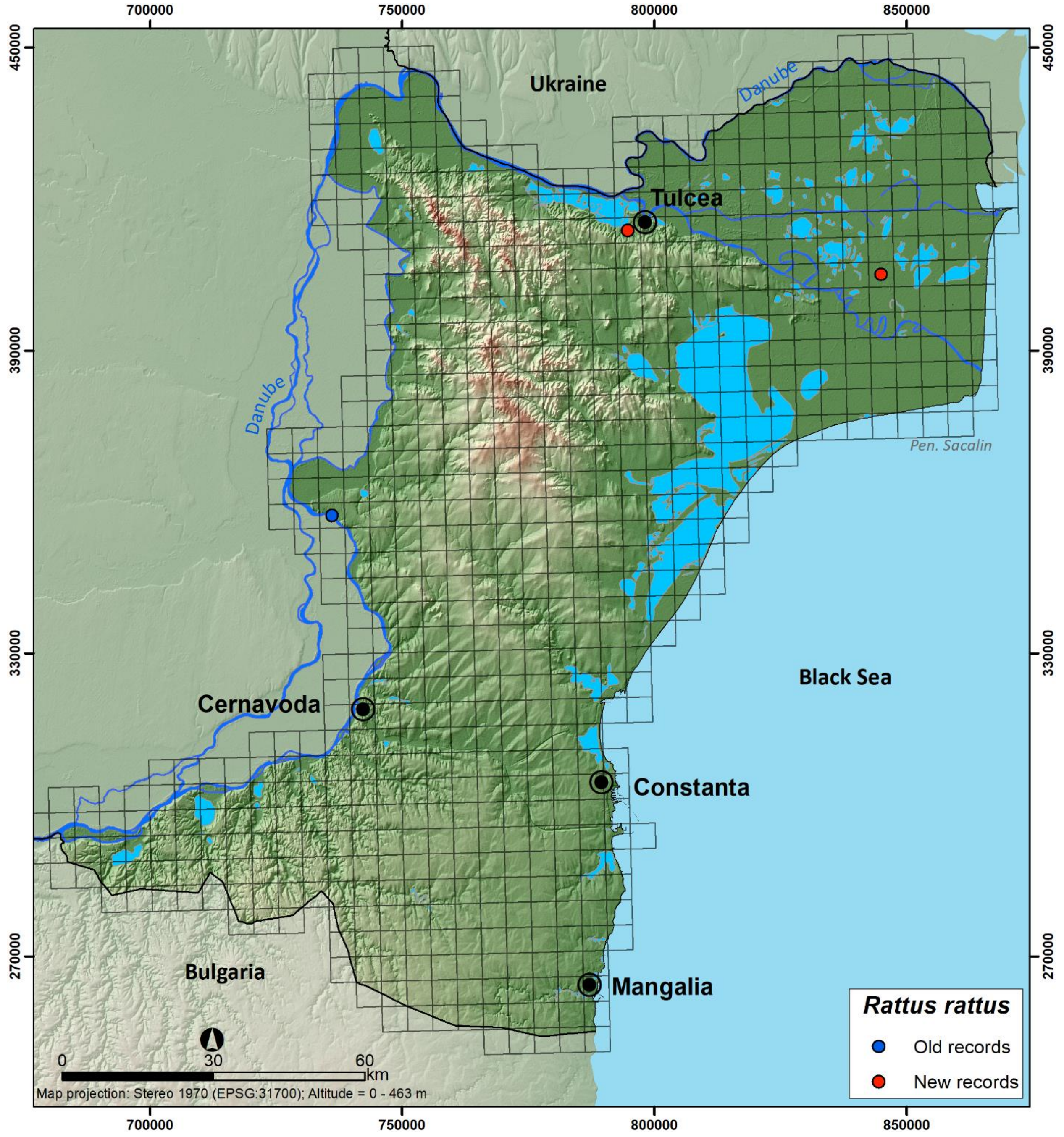
700000
750000
800000
850000
450000
390000
330000
270000
Ukraine
Danube
Tulcea
Danube
Pen. Sacalin
Black Sea
Cernavoda
Constanta
Bulgaria
Mangalia
Rattus rattus
Old records
New records
0
30
60
km
Map projection: Stereo 1970 (EPSG:31700); Altitude = 0 - 463 m

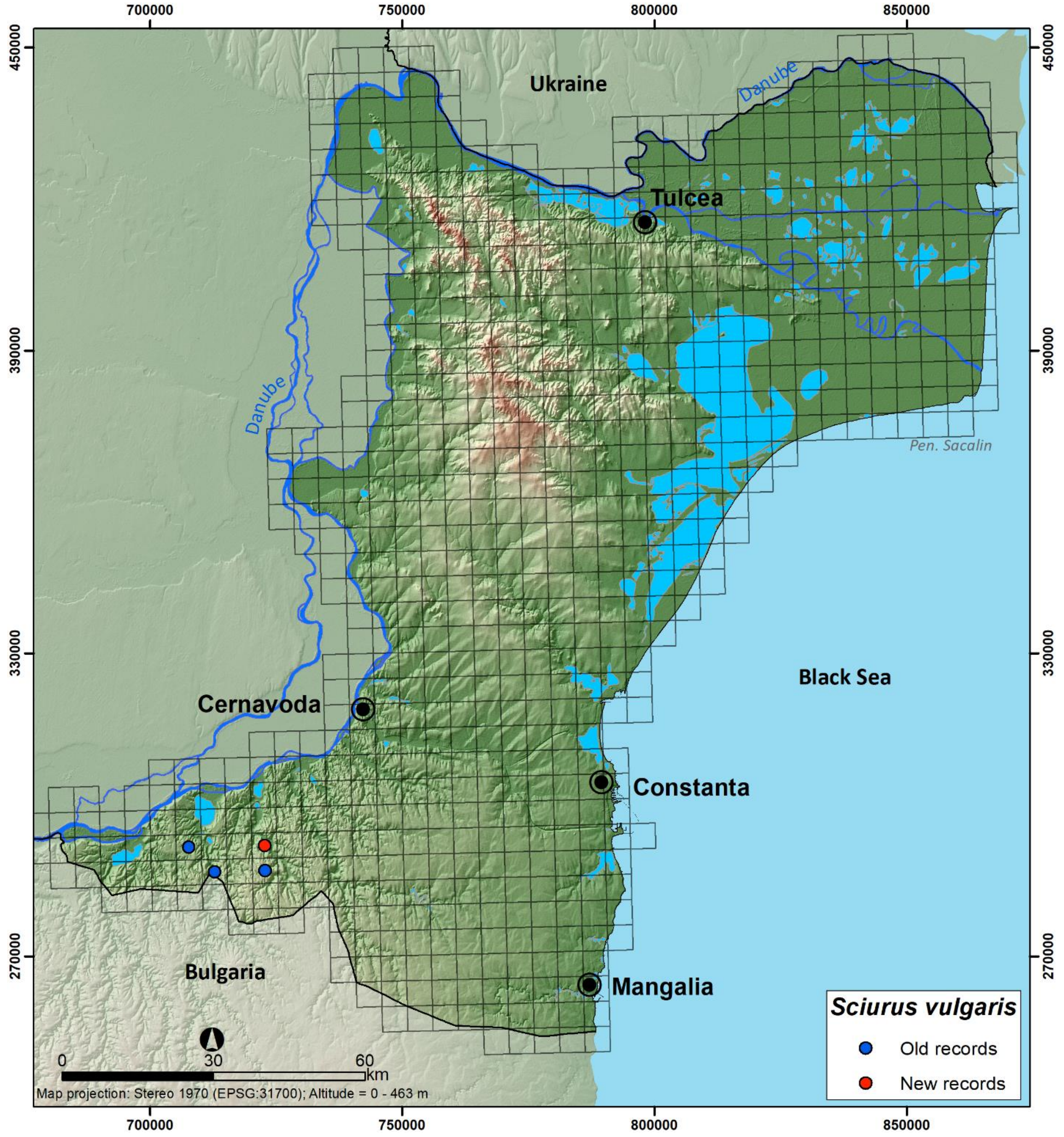

700000
750000
800000
850000
450000
390000
330000
270000
Ukraine
Danube
Tulcea
Danube
Pen. Sacalin
Cernavoda
Constanta
Black Sea
Bulgaria
Mangalia
Sciurus vulgaris
Old records
New records
0
30
60
km
Map projection: Stereo 1970 (EPSG:31700); Altitude = 0 - 463 m

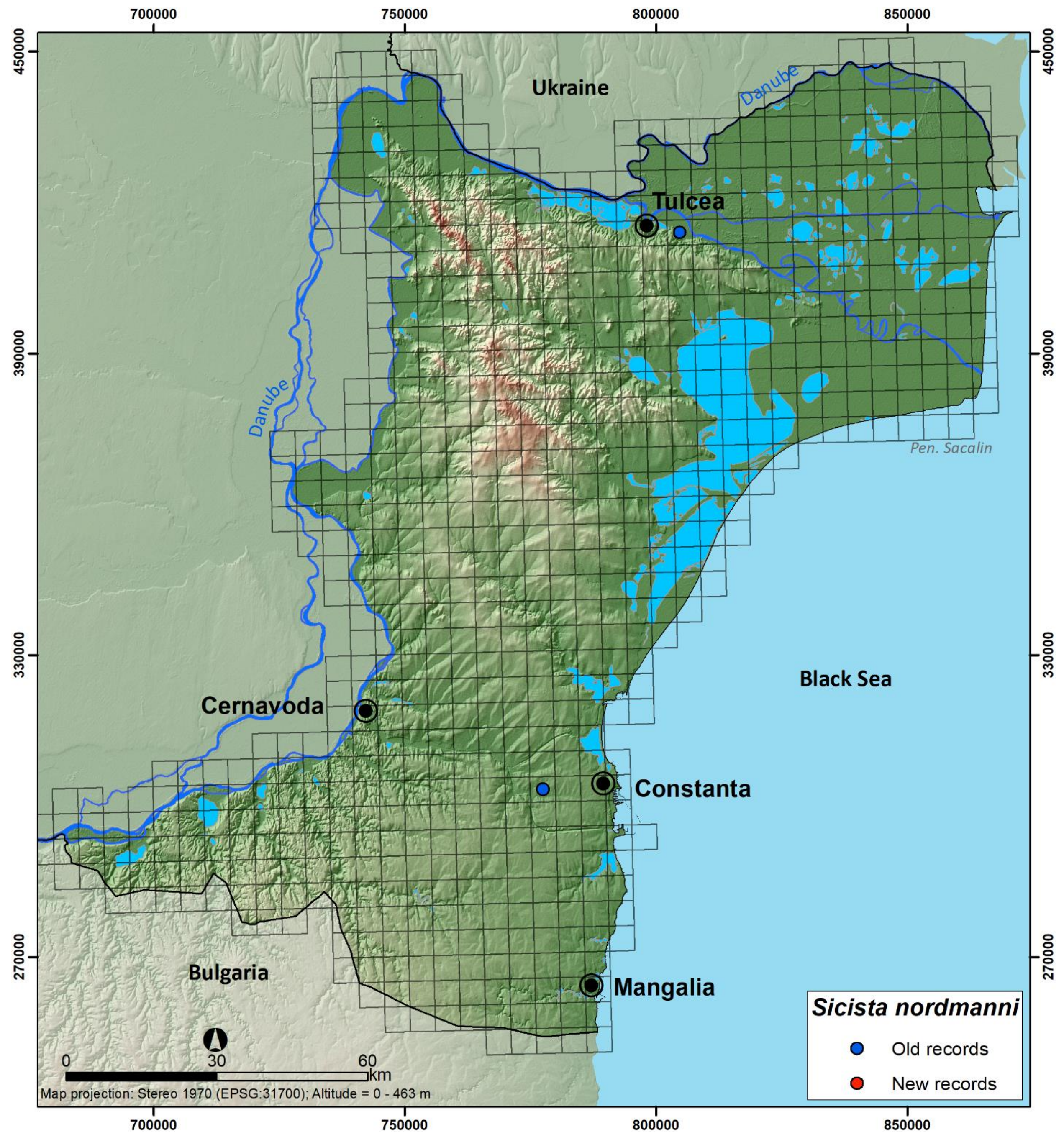
700000
750000
800000
850000
450000
390000
330000
270000
Ukraine
Danube
Tulcea
Danube
Pen. Sacalin
Black Sea
Cernavoda
Constanta
Bulgaria
Mangalia
0
30
60
km
Map projection: Stereo 1970 (EPSG:31700); Altitude = 0 - 463 m
Sicista nordmanni
Old records
New records

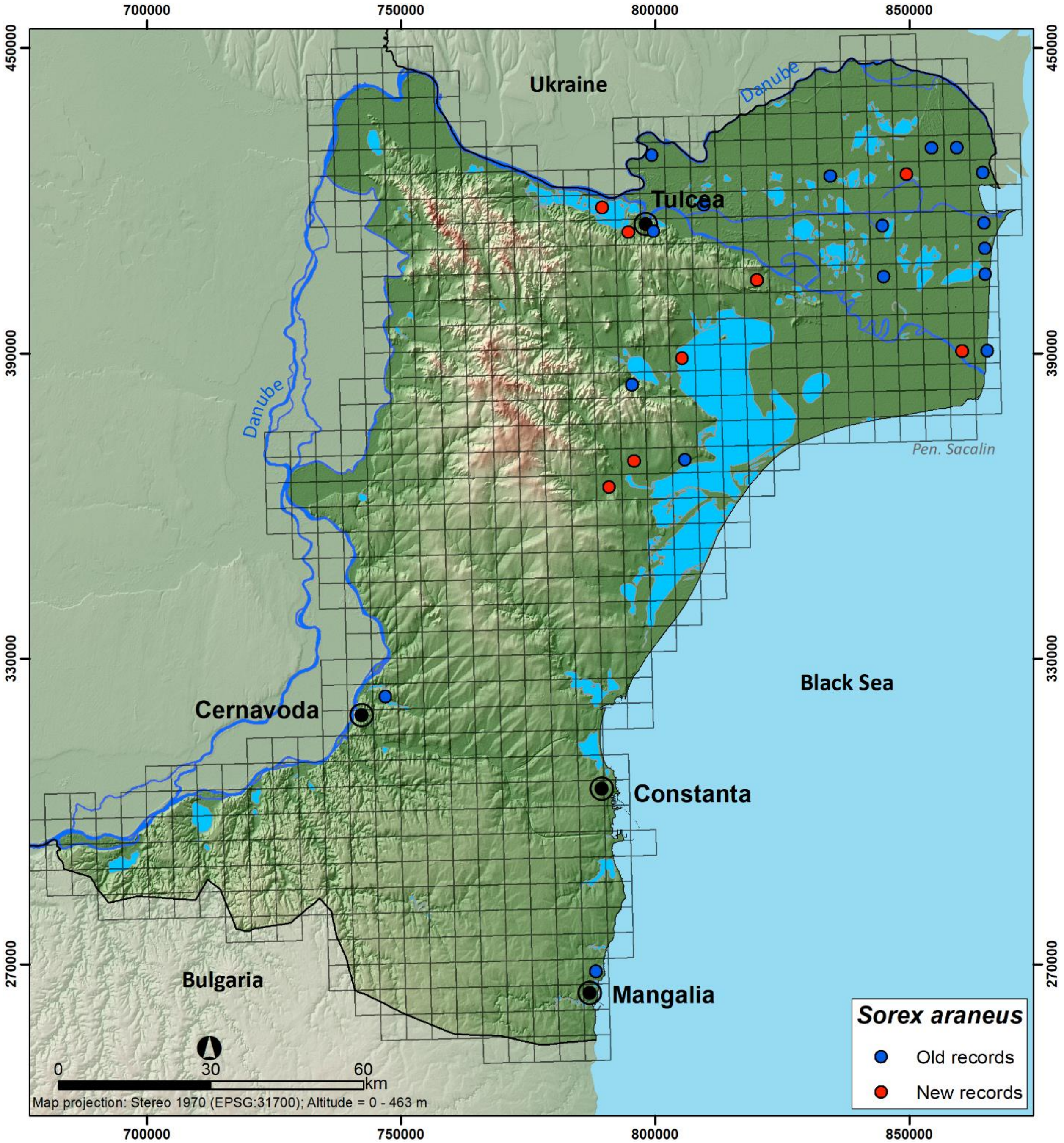

700000
750000
800000
850000
450000
390000
330000
270000
Ukraine
Danube
Tulcea
Pen. Sacalin
Black Sea
Cernavoda
Constanta
Mangalia
Bulgaria
Sorex araneus
Old records
New records
0
30
60
km
Map projection: Stereo 1970 (EPSG:31700); Altitude = 0 - 463 m

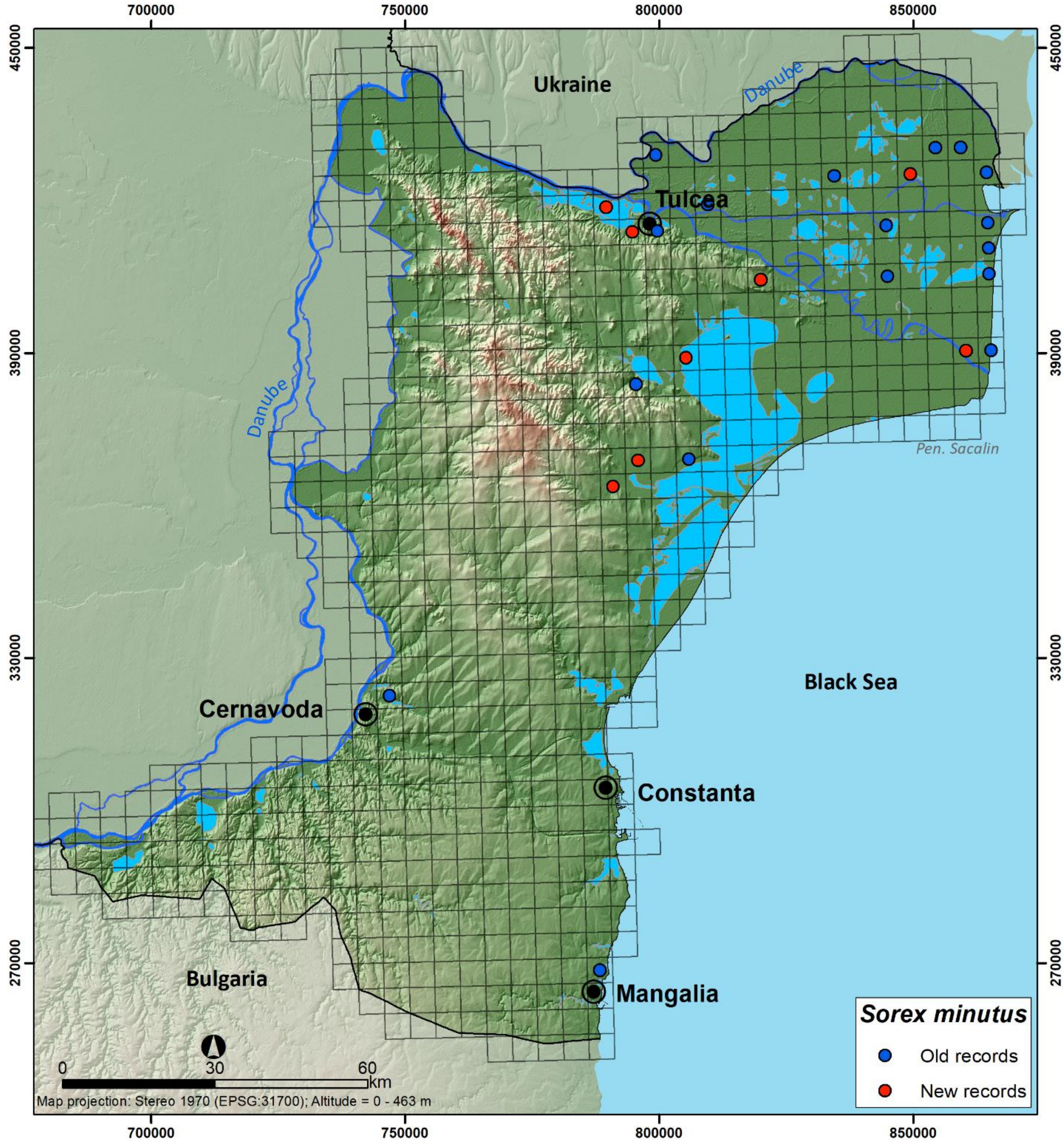
700000
750000
800000
850000
450000
390000
330000
270000
Ukraine
Danube
Tulcea
Pen. Sacalin
Black Sea
Cernavoda
Constanta
Bulgaria
Mangalia
Sorex minutus
Old records
New records
0
30
60
km
Map projection: Stereo 1970 (EPSG:31700); Altitude = 0 - 463 m

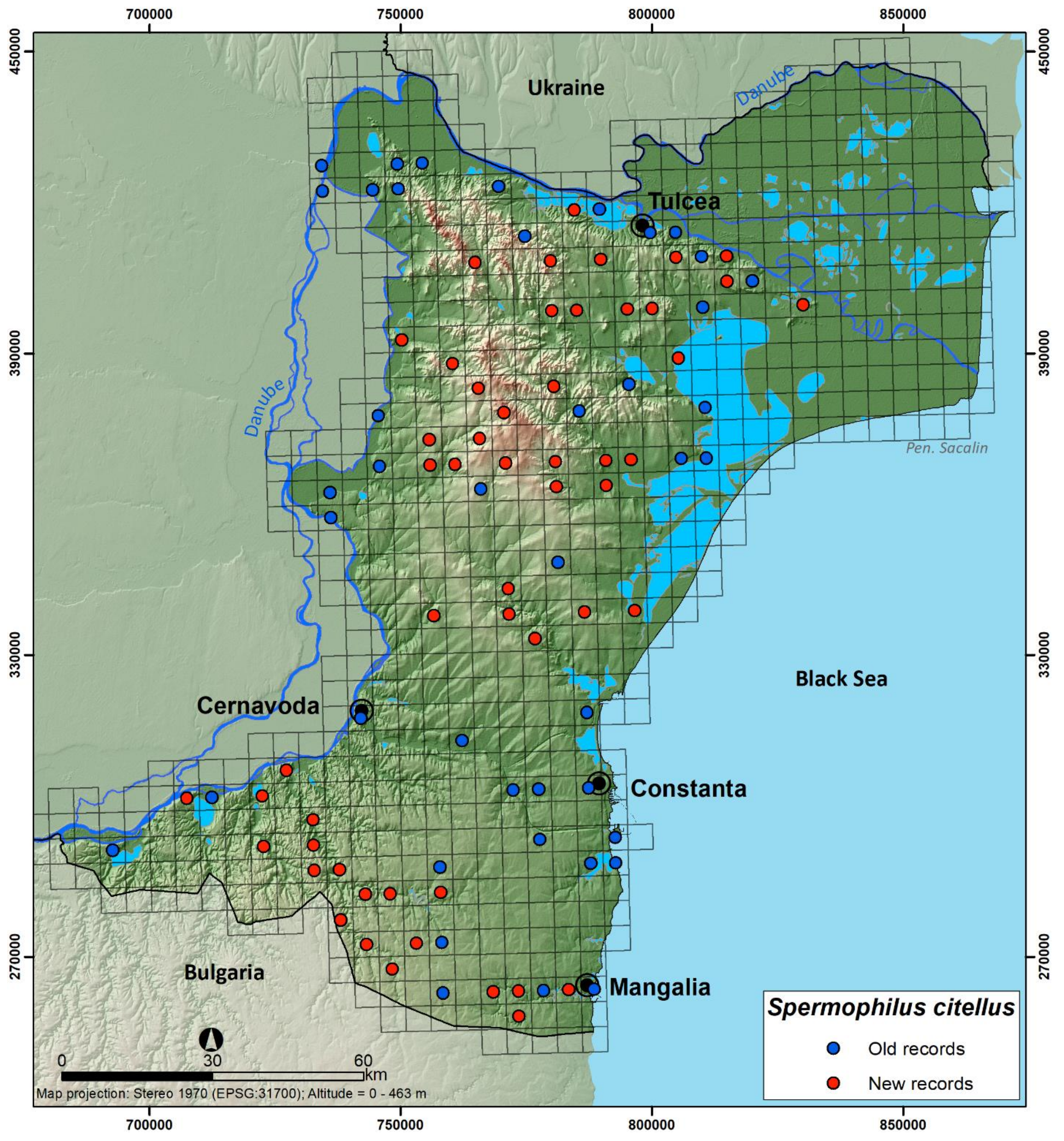
700000
750000
800000
850000
450000
390000
330000
270000
Ukraine
Danube
Tulcea
Danube
Pen. Sacalin
Black Sea
Cernavoda
Constanta
Bulgaria
Mangalia
Spermophilus citellus
Old records
New records
0
30
60
km
Map projection: Stereo 1970 (EPSG:31700); Altitude = 0 - 463 m

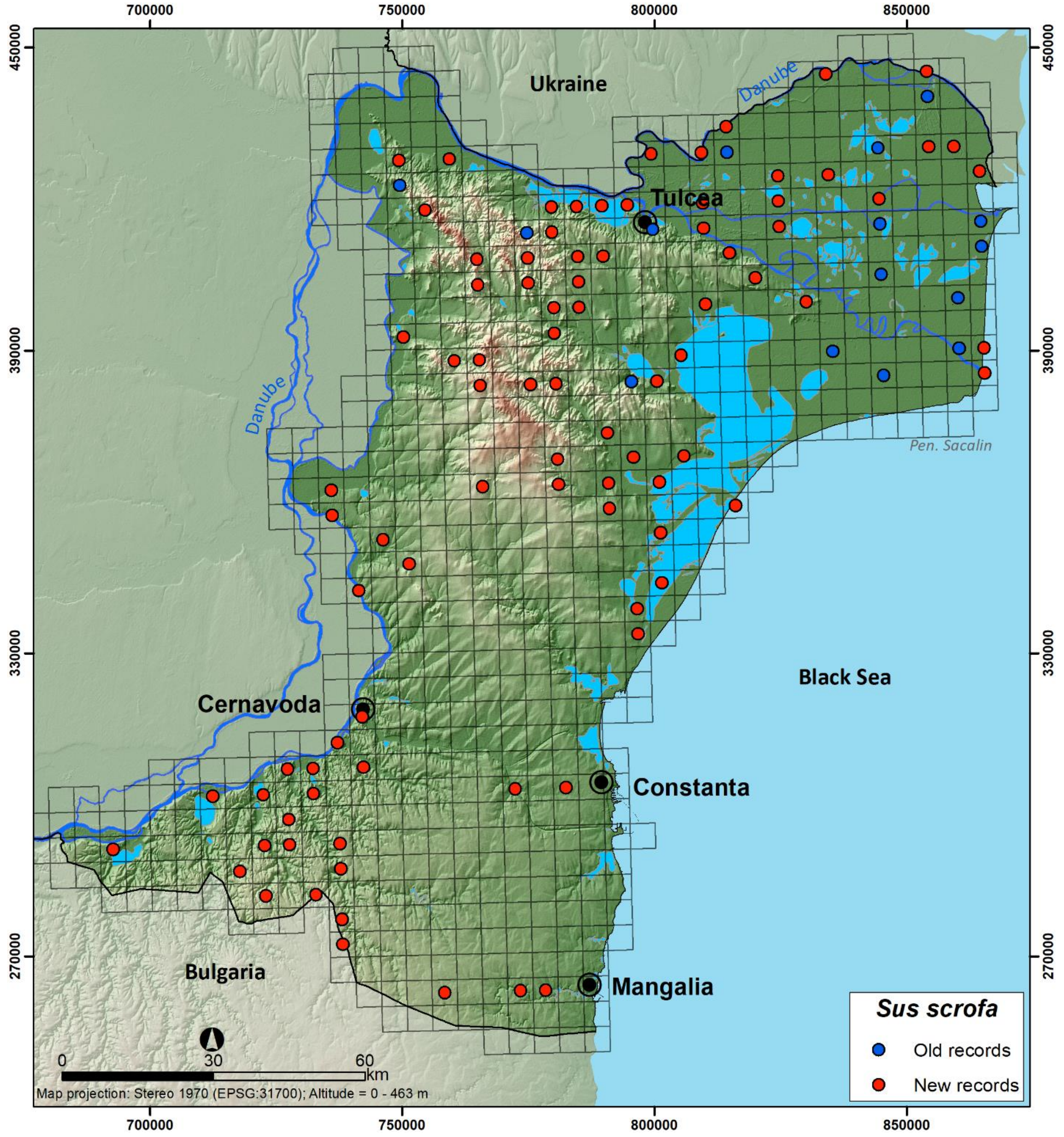
700000
750000
800000
850000
450000
390000
330000
270000
Ukraine
Danube
Tulcea
Pen. Sacalin
Black Sea
Cernavoda
Constanta
Mangalia
Bulgaria
Sus scrofa
Old records
New records
0
30
60
km
Map projection: Stereo 1970 (EPSG:31700); Altitude = 0 - 463 m

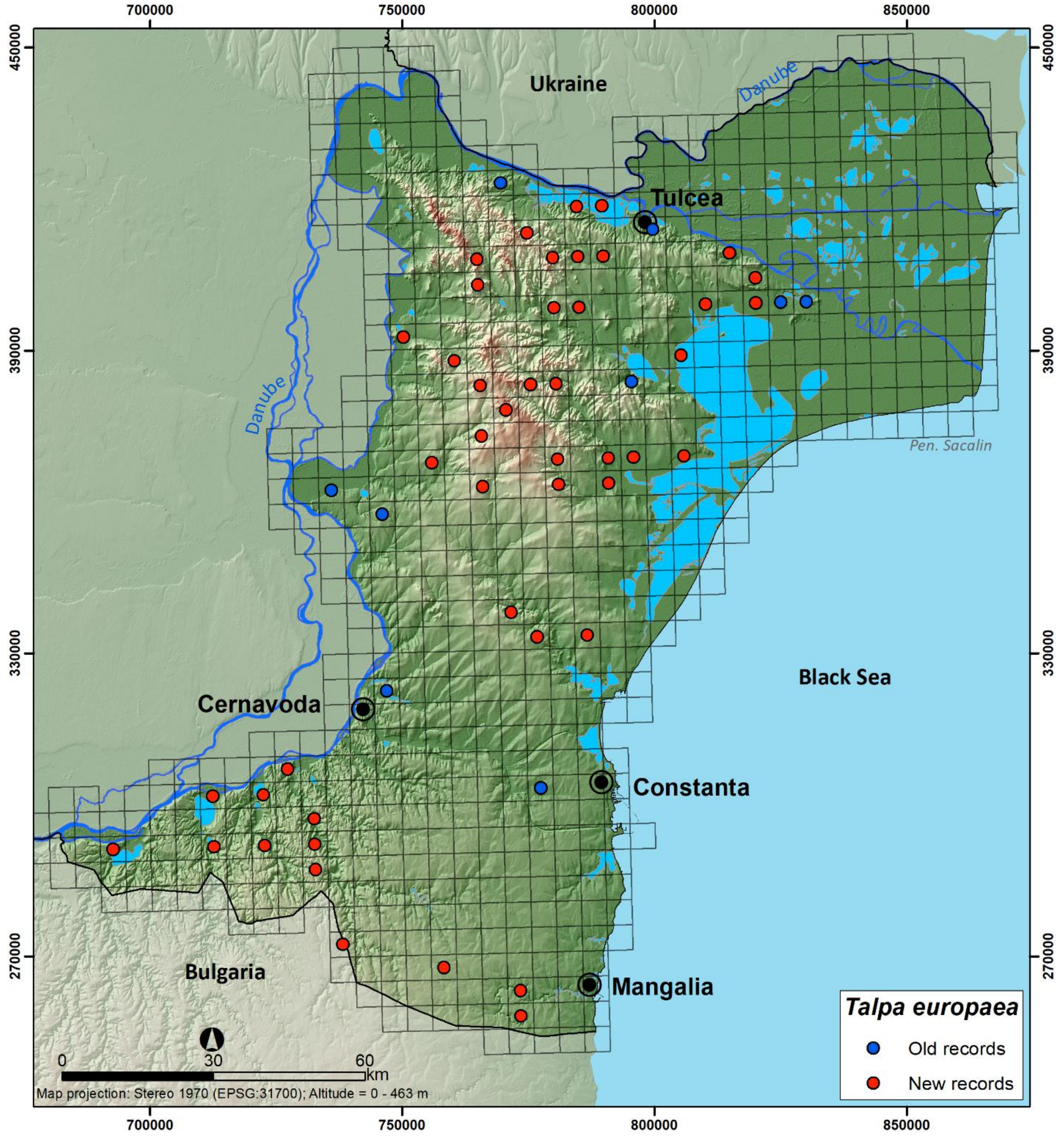
700000
750000
800000
850000
450000
390000
330000
270000
Ukraine
Danube
Tulcea
Pen. Sacalin
Black Sea
Cernavoda
Constanta
Bulgaria
Mangalia
0
30
60
km
Map projection: Stereo 1970 (EPSG:31700); Altitude = 0 - 463 m
Talpa europaea
Old records
New records

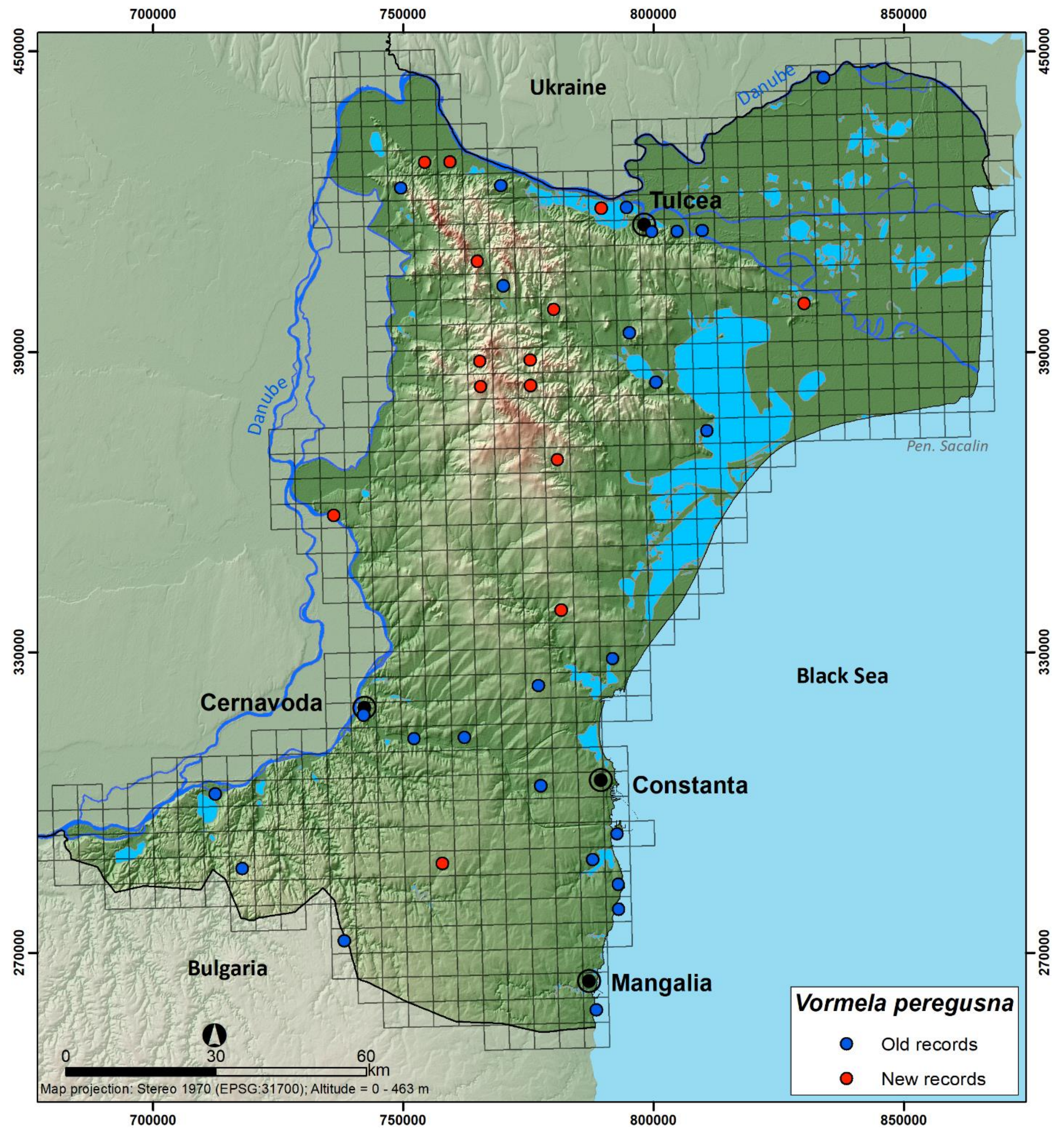
Ukraine
Danube
Tulcea
Danube
Pen. Sacalin
Black Sea
Cernavoda
Constanta
Bulgaria
Mangalia
Vormela peregusna
Old records
New records
0
30
60
km
Map projection: Stereo 1970 (EPSG:31700); Altitude = 0 - 463 m
700000
750000
800000
850000
450000
390000
330000
270000

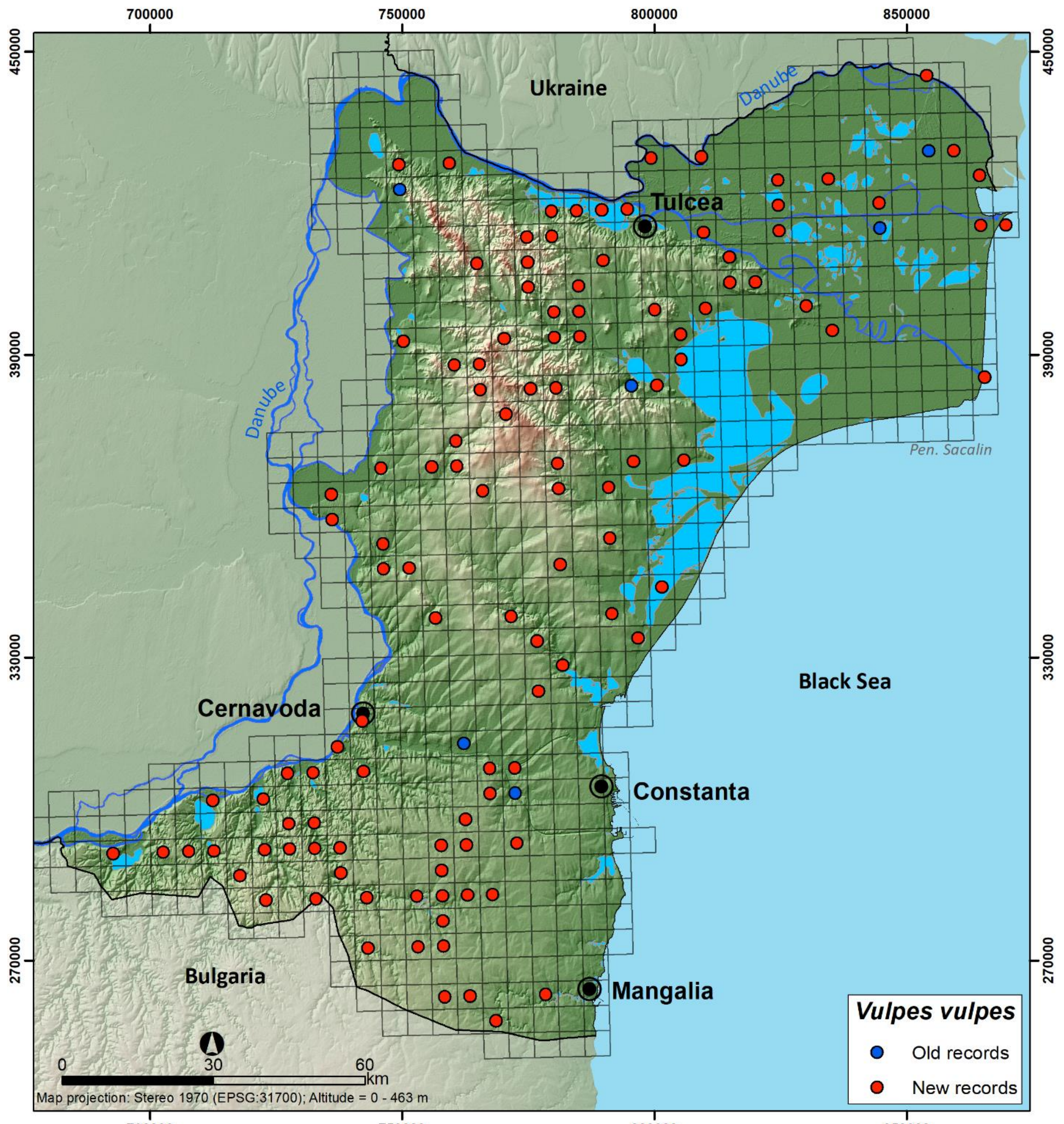
700000
750000
800000
850000
450000
390000
330000
270000
Ukraine
Danube
Tulcea
Pen. Sacalin
Black Sea
Cernavoda
Constanta
Mangalia
Bulgaria
Vulpes vulpes
Old records
New records
0
30
60
km
Map projection: Stereo 1970 (EPSG:31700); Altitude = 0 - 463 m
