# Supplementary material for: Conservation priorities for terrestrial mammals in Dobrogea Region, Romania
Source: Zookeys. 2018 Oct 23;(792):133–58. doi: 10.3897/zookeys.792.25314 (PMC6215976; doi:10.3897/zookeys.792.25314)
Supplement: Supplementary material 3 — Methodology used to identify high-priority Natura 2000 sites with Zonation v4 [file zookeys-792-133-s003.pdf]

Iulia V. Miu, Gabriel B. Chișamera, Viorel D. Popescu, Ruben Iosif, Andreea Nita, Steluta Manolache, Viorel D. Gavril, Ioana Cobzaru, Laurentiu Rozylowicz (2018) Conservation priorities for terrestrial mammals in Dobrogea Region, Romania. Zookeys

Appendix 3

Methodology used to identify high-priority Natura 2000 sites with Zonation v4.

Please see Lehtomäki & Moilanen, 2013 for a detailed description of the Zonation software and workflow

Step 1 - Zonation algorithm. The Zonation algorithm used for this analysis processes iterations to eliminate spatial units (e.g., grid cells) that have the lowest total marginal loss in biodiversity. Meanwhile, it accounts for the distribution of total and remaining characteristics of the analysis. At each iteration, the characteristics are normalized by maintaining the size of their category, which means that if a characteristic becomes less frequent during the cell removal process, its relative significance increases. Repeated normalization of the category size leads to maintaining a balance between all the characteristics of all iterations. At the end, Zonation has heuristically ranked the entire landscape according to its conservation priority.

Step 2 - Zonation input layers. The Zonation algorithm uses multiple biodiversity input features such as species distribution data, habitat types, ecosystem services, costs for protection; (Moilanen et al. 2014). We used layers for presence/absence data of species distribution for each of the 14 species listed in Annexes II and IV of the Habitats Directive, at a 5×5 km resolution.

Step 3 - Hierarchic mask. Zonation uses broad-scale grids like mask layers specifying prior land uses, such as the present protected area network. It is a raster file where integer values are assigned to cells. Cells with lower mask levels (small numbers) are removed before cells with higher mask levels (large numbers), thus making a forced multi-level hierarchy possible in the analysis. In our case, we used a hierarchic mask of Natura 2000 network in Dobrogea, because we wanted to identify high-priority Natura 2000 sites.

Step 4 - Additive benefit function. Zonation provides four cell removal rules (Core-area Zonation, Additive benefit function, Target-based planning and Generalized benefit function).

For our case study, we used Additive benefit function, an algorithm which treats conservation value as additive across biodiversity features. This function implicitly takes into account all features in a given cell according to their importance, instead of using the one feature that has the highest value (see page 33 in Zonation v4 manual). For this reason, we considered the Additive benefit function a suitable algorithm to integrate the mammal species distribution listed in Annexes II and IV of the Habitats Directive according to their individual importance into a single prioritization model.

Step 5 - Quantitative assessment of the prioritization maps. Zonation outputs are hierarchies of cells with conservation ranking between 0 (lowest value) and 1 (top priorities). The 0 – 1 ranks have a uniform distribution, thus the top spatial conservation priorities (e.g., top 20% of landscape) have a Zonation ranking > 0.80). The uniform distribution of the conservation ranking has the advantage of providing flexibility when identifying top areas for conservation. We considered as high-priority areas for conservation, all pixels falling in the top 20% of the predicted priority ranks, a proportion which maximizes mammal species representation at the region level.

Literature cited

Lehtomäki J, Moilanen A (2013) Methods and workflow for spatial conservation prioritization using Zonation. Environmental Modelling & Software 47: 128–137. doi: 10.1016/j.envsoft.2013.05.001

Moilanen A, Pouzols FM, Meller L, Veach V, Arponen A, Leppänen J, Kujala H (2014) Spatial conservation planning methods and software ZONATION. User Manual. 288 pp. doi: 10.1017/CBO9781107415324.004
